# On the incompatibility of lithium–O_2_ battery technology with CO_2_ †
†Electronic supplementary information (ESI) available: Full experimental, crystallographic and spectroscopic data. CCDC 1512972. For ESI and crystallographic data in CIF or other electronic format see DOI: 10.1039/c7sc01230f



**DOI:** 10.1039/c7sc01230f

**Published:** 2017-06-20

**Authors:** Shiyu Zhang, Matthew J. Nava, Gary K. Chow, Nazario Lopez, Gang Wu, David R. Britt, Daniel G. Nocera, Christopher C. Cummins

**Affiliations:** a Department of Chemistry , Massachusetts Institute of Technology , 77 Massachusetts Avenue , Cambridge , MA 02139-4307 , USA . Email: ccummins@mit.edu ; Tel: +1 617 253 5332; b Department of Chemistry , University of California , Davis, One Shields Avenue , Davis , CA 95616 , USA; c Department of Chemistry , Queen’s University , 90 Bader Lane , Kingston , Ontario K7L3N6 , Canada; d Department of Chemistry and Chemical Biology , Harvard University , 12 Oxford Street , Cambridge , MA 02138 , USA

## Abstract

When solubilized in a hexacarboxamide cryptand anion receptor, the peroxide dianion reacts rapidly with CO_2_ in polar aprotic organic media to produce hydroperoxycarbonate (HOOCO_2_^–^) and peroxydicarbonate (^–^O_2_COOCO_2_^–^). Peroxydicarbonate is subject to thermal fragmentation into two equivalents of the highly reactive carbonate radical anion, which promotes hydrogen atom abstraction reactions responsible for the oxidative degradation of organic solvents. The activation and conversion of the peroxide dianion by CO_2_ is general. Exposure of solid lithium peroxide (Li_2_O_2_) to CO_2_ in polar aprotic organic media results in aggressive oxidation. These findings indicate that CO_2_ must not be introduced in conditions relevant to typical lithium–O_2_ cell configurations, as production of HOOCO_2_^–^ and ^–^O_2_COOCO_2_^–^ during lithium–O_2_ cell cycling will lead to cell degradation *via* oxidation of organic electrolytes and other vulnerable cell components.

## Abstract 2

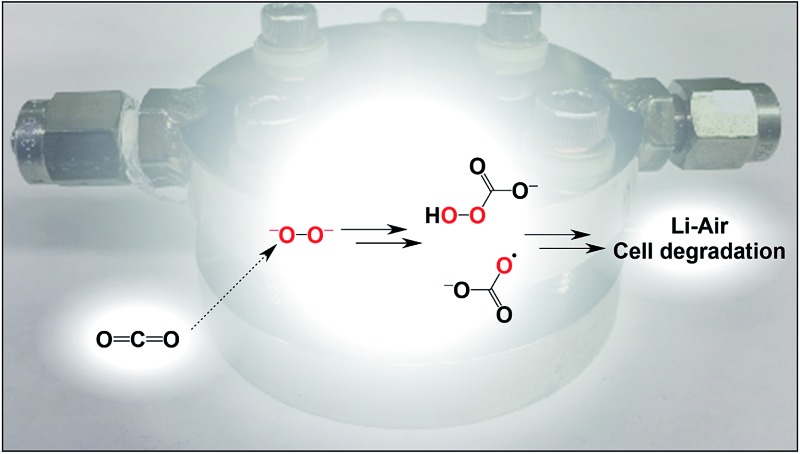
The peroxide dianion reacts with CO_2_ in polar aprotic organic media to afford the hydroperoxycarbonate and carbonate radical anions. These highly reactive species, if formed in lithium–O_2_ cells, can lead to cell degradation *via* oxidation of the electrolyte and electrode.

## Introduction

The two-electron reduction of molecular oxygen to the peroxide dianion is an attractive cathode redox couple for developing rechargeable lithium–O_2_ batteries.^1^ Lithium carbonate (Li_2_CO_3_) formation is deleterious to battery performance because it passivates electrodes and causes a drastic reduction in the round trip efficiency of discharge–charge cycles.^2,3^ Carbonate formation is typically ascribed to oxidative degradation of organic electrolytes^4–6^ and carbon electrodes^7^ by superoxide^8,9^ and singlet oxygen.^10^ Although peroxide is often considered to be a strong oxidant in aqueous media, salts of its dianion (O_2_^2–^) are poor oxidizers in organic media due to their extremely low solubility and so, for this reason, the possible role of peroxide in furnishing carbonate is underappreciated.^11^ The presence of carbonate-derived CO_2_ during the recharge cycle of lithium–O_2_ batteries^2^ prompted us to consider the possibility that carbonate formation may be a consequence of peroxide combination with carbon dioxide; this would likely confer increased solubility and yield powerful oxidizers. To address this topic, we utilized an anion-receptor solubilized form of the peroxide dianion^12^ to elucidate the molecular level details of its reaction with carbon dioxide. As reported herein, we observed the formation of strongly oxidizing peroxy(di)-carbonate intermediates and studied their reaction with organic solvents to produce carbonate. In a complementary line of investigation, we showed that carbon dioxide activation of insoluble Li_2_O_2_ similarly engenders solvent oxidation with the concomitant production of carbonate. Our findings shed light on the identity and behavior of the hot oxidants generated upon the facile and quantitative combination of O_2_^2–^ with CO_2_ *via* direct spectroscopic detection and exploratory reaction chemistry.

## Results and discussion

### Reaction of O_2_^2–^ with CO_2_ using an anion receptor

**Fig. 1:**
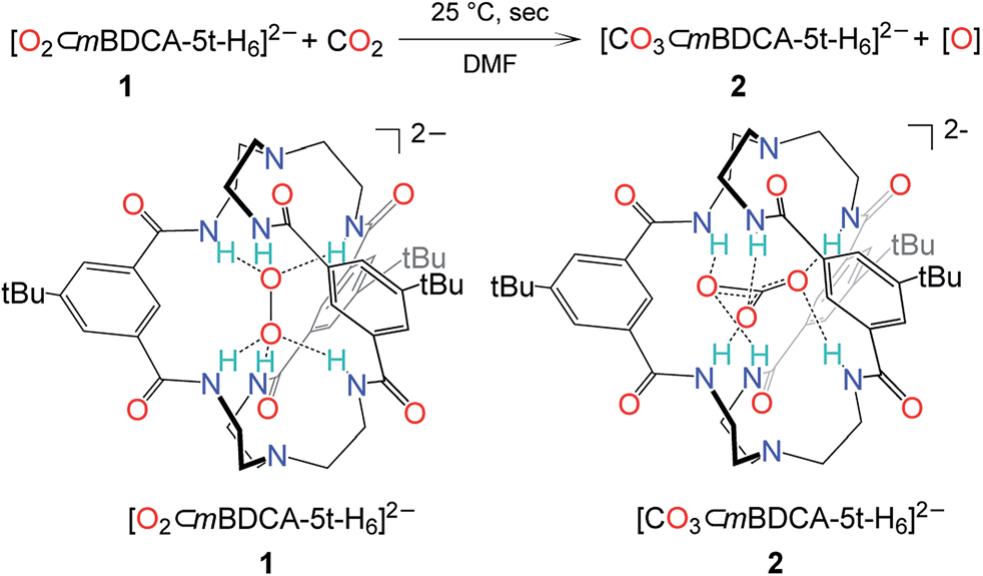
The reaction scheme of peroxide cryptate **1** with CO_2_ and a line drawing of [O_2_⊂*m*BDCA-5t-H_6_]^2–^ and [CO_3_⊂*m*BDCA-5t-H_6_]^2–^.

Despite the drastic and deleterious effect that CO_2_ has upon the performance of a cycling lithium–oxygen battery, our understanding of the chemical entities responsible for this effect is poor and based primarily upon computational studies or observation of terminal reaction products.^2,8^ To examine the effect of CO_2_ on the oxidative power of peroxide, an anion receptor complex^13^ of the peroxide dianion, [O_2_⊂*m*BDCA-5t-H_6_]^2–^ (**1**, Fig. 1),^12^ was employed as a soluble source of peroxide dianion. The anion receptor *m*BDCA-5t-H_6_ encapsulates the peroxide dianion *via* six N–H···O hydrogen bonds. Since its discovery, this cryptate has enabled exploration of the reactivity of the peroxide dianion with small molecules in polar organic media without the complicating influence of acidic protons.^12,14^ Despite being a simple molecule, the peroxide dianion has yielded rich and previously unknown chemistry, including metal-free oxidation of carbon monoxide (CO) generating carbonate, which is encapsulated by the anion receptor as [CO_3_⊂*m*BDCA-5t-H_6_]^2–^ (**2**, Fig. 1).^14^

**Fig. 2:**
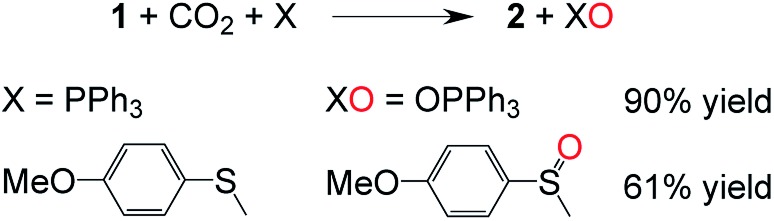
Addition of CO_2_ to **1** in the presence of an oxygen-atom acceptor.

While the conversion of **1** to **2** under CO (1 atm, 40 °C) takes two hours to go to completion, exposing a dimethylformamide-*d*_7_ (DMF-*d*_7_) solution of **1** to CO_2_ (1 atm, 25 °C) resulted in the essentially instantaneous formation of carbonate cryptate [CO_3_⊂*m*BDCA-5t-H_6_]^2–^ as indicated by ^1^H NMR spectroscopy. Formation of O_2_ gas was not observed by gas chromatography (GC) analysis of the reactor headspace gases,^15^ suggesting the possibility of oxygen incorporation into the solvent molecules. To probe the fate of the “missing oxygen atom” according to the equation at the top of Fig. 1, the reaction of CO_2_ and **1** was next performed in the presence of oxygen atom acceptors. While **1** on its own is unreactive towards PPh_3_ and methoxythioanisole at 25 °C, exposing a mixture of **1** and an organic oxygen-atom acceptor to CO_2_ (1 atm, 25 °C) resulted in the rapid formation of triphenylphosphine oxide (90%, Fig. 2) or 1-(methylsulfinyl)-4-methoxybenzene (61%, Fig. 2), respectively.

**Fig. 3:**
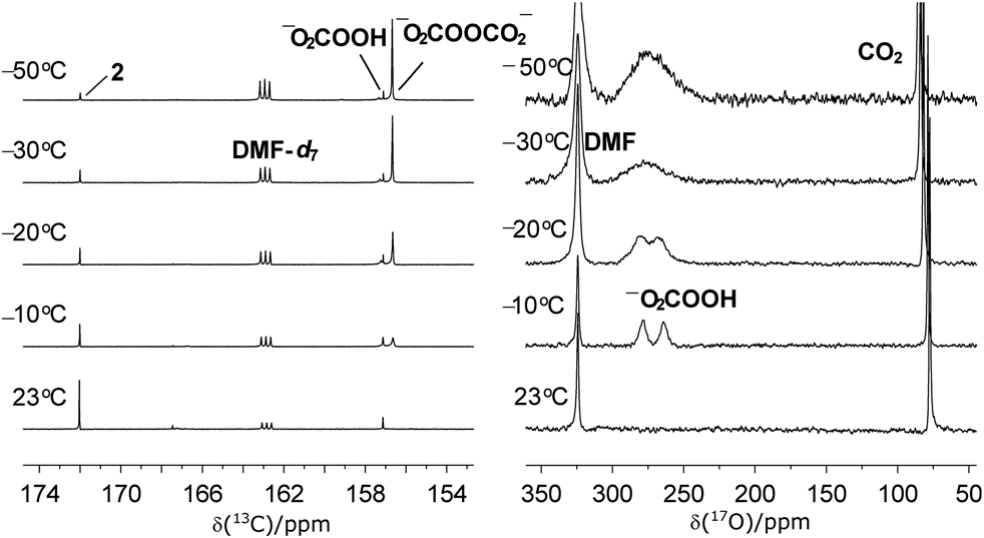
Variable temperature ^13^C NMR (left) and ^17^O NMR (right) analysis of the reaction between ^13^CO_2_ and **1**.

Aiming to establish the chemical identity of the oxidant(s) generated upon exposure of peroxide cryptate **1** to CO_2_, we followed the reaction by variable temperature ^13^C NMR spectroscopy. A strong new signal at *δ* = 156.9 ppm, together with one minor species resonating at *δ* = 157.4 ppm, was observed at –50 °C (Fig. 3). We first considered peroxycarbonate (^–^OOCO_2_^–^,Fig. 3) and hydroperoxycarbonate (HOOCO_2_^–^, Fig. 3) as candidates to correspond to the observed ^13^C NMR signals, since hydroperoxycarbonate is known to be active for sulfide oxidation.^16,17^ The salt [PPN][HOO^13^CO_2_] (PPN = bis(triphenylphosphine)iminium), which was generated *in situ* from H_2_O_2_ and bicarbonate [PPN][H^13^CO_3_] (*δ* = 160.0 ppm),^18–20^ showed a single ^13^C resonance at *δ* = 157.5 ppm, confirming the identity of the minor intermediate as HOOCO_2_^–^.

**Fig. 4:**
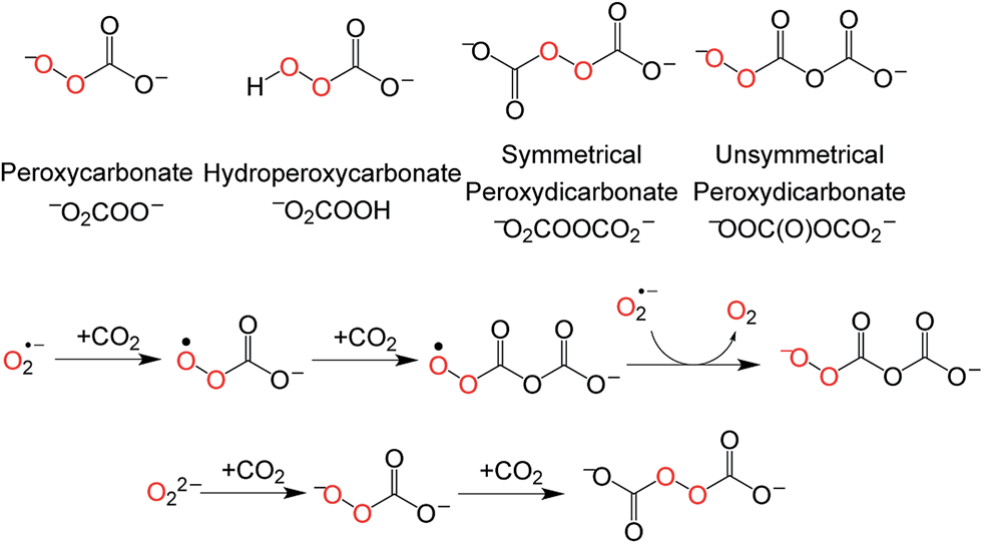
Possible intermediates during the conversion of **1** and CO_2_ to **2** (top) and formation of symmetric and unsymmetric peroxydicarbonate (bottom).

Moreover, ^13^C Gauge-Independent Atomic Orbital (GIAO) NMR calculations of the chemical shifts of potential candidates were performed.^15^ From a range of potential chemical species (Fig. 4), symmetric peroxydicarbonate (^–^O_2_COOCO_2_^–^) emerged as the most plausible assignment for the major product at *δ* = 156.9 ppm, having the best match between the observed and calculated ^13^C NMR chemical shift.^15^ In an effort to independently generate ^–^O_2_COOCO_2_^–^, an experiment was carried out in which excess ^13^CO_2_ was added to a frozen mixture of potassium *tert*-butoxide and bis(trimethylsilyl) peroxide giving rise to a single new ^13^C NMR resonance at *δ* = 155.5 ppm (–40 °C), tentatively supporting our identification of the major **1** + CO_2_ product as symmetric peroxydicarbonate. Differences in the medium and reaction conditions may account for the observed chemical shift difference (155.5 ppm here *versus* 156.9 ppm, above). Similarly, superoxide (O_2_˙^–^) has been documented to absorb two equivalents of CO_2_, generating unsymmetrical peroxydicarbonate (Fig. 4) as a precipitate.^21^ In our hands, the low solubility of this unsymmetrical peroxydicarbonate material precluded its characterization by solution ^13^C NMR studies under conditions we employed successfully for *in situ* characterization of ^–^O_2_COOCO_2_^–^ and HOOCO_2_^–^. This establishes that different oxidants are generated upon addition of CO_2_ to superoxide as compared with the peroxide dianion (Fig. 4).

**Fig. 5:**
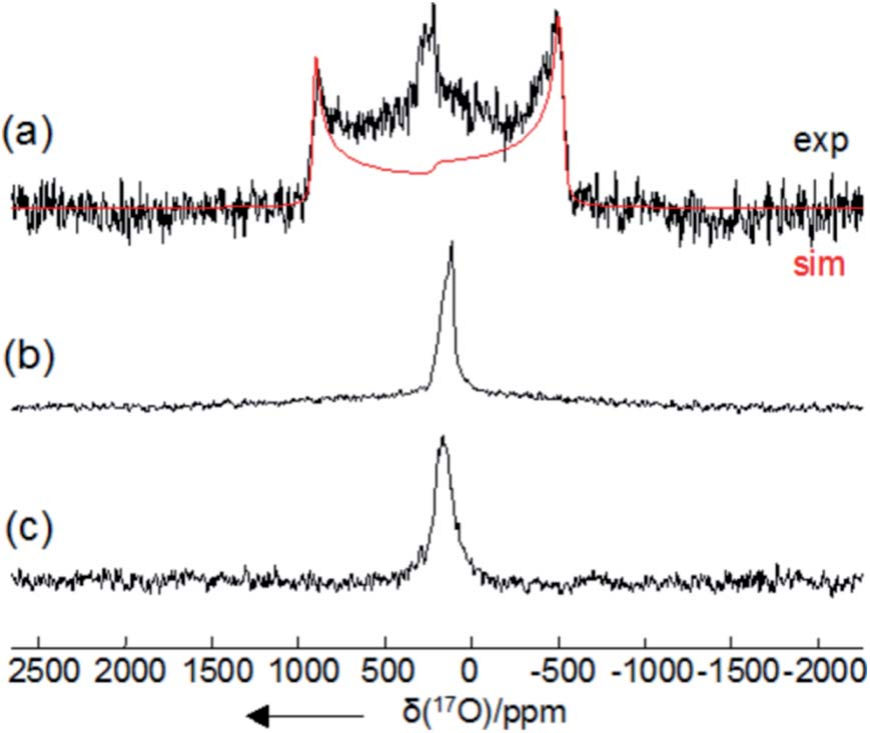
Experimental (black trace) and simulated (red trace) solid-state ^17^O NMR spectra of (a) **1**-^17^O_2_, (b) **2**-CO^17^O_2_, and (c) product resulting from the treatment of solid **1**-^17^O_2_ with CO_2_. All solid-state ^17^O NMR experiments were performed on a Bruker Avance-600 (14.1 T) spectrometer under static conditions. A Hahn echo sequence was used for recording the static spectra to eliminate the acoustic ringing from the probe. A 4 mm Bruker MAS probe was used without sample spinning. The effective 90° pulse was of a duration of 1.7 μs. High power ^1^H decoupling (70 kHz) was applied in all static experiments. A liquid H_2_O sample was used for both RF power calibration and ^17^O chemical shift referencing (*δ* = 0 ppm).

Further support for the formation of HOOCO_2_^–^ and ^–^O_2_COOCO_2_^–^ upon interaction of CO_2_ with peroxide sources was provided by variable temperature ^17^O NMR spectroscopy. Due to the fast relaxation times of ^17^O nuclei, observation of the ^17^O resonance for mid-size molecules such as **1**-^17^O_2_ and **2**-CO^17^O_2_ was expected to be challenging in solution.^22^ Indeed, ^17^O NMR measurements of independently prepared peroxide cryptate **1**-^17^O_2_ and carbonate cryptate **2**-CO^17^O_2_ (70%, ^17^O-enriched) showed no resonances between *δ* = –1100 and +1800 ppm (H_2_O used as a reference, *δ* = 0 ppm) in DMF. However, solid-state ^17^O NMR measurements for **1**-^17^O_2_ and **2**-CO^17^O_2_ were successful, as reported previously in the case of **2**-CO^17^O_2_,^14^ and in the present work for **1**-^17^O_2_, providing the benchmark ^17^O NMR chemical shifts (*δ* = 260 ppm for **1**-^17^O_2_ and 170 ppm for **2**-CO^17^O_2_) (Fig. 5, Table 1). As seen in Fig. 3, 70% ^17^O-enriched samples of HOOCO_2_^–^ and ^–^O_2_COOCO_2_^–^ generated in DMF solution at –78 °C from the reaction of **1**-^17^O_2_ and ^13^CO_2_ resulted in a broad ^17^O NMR resonance at *δ* = 275.3 ppm, assigned as overlapping signals of HOOCO_2_^–^ and ^–^O_2_COOCO_2_^–^. Upon gradual warming of the sample to –10 °C, the intensity of the signal decayed; the signal ultimately resolved into two peaks with equal intensities at *δ* = 278.7 and 264.0 ppm, distinct from those observed for **1**-^17^O_2_ and **2**-CO^17^O_2_. The two peaks observed are attributed to HOOCO_2_^–^ which contains two chemically inequivalent ^17^O atoms (*δ* = 278.7 ppm for H**O**OCO_2_^–^ and 264.0 ppm for HO**O**CO_2_^–^), in contrast to the situation for ^–^O_2_COOCO_2_^–^ in which the peroxy oxygen atoms are related by symmetry. The appearance of the relatively sharp ^17^O NMR signals assigned to HOOCO_2_^–^ coupled with the concurrent observation of monodeprotonated cryptand ([*m*BDCA-5t-H_5_]^–^)^14^ by ^1^H NMR spectroscopy strongly suggests that HOOCO_2_^–^ is not strongly sequestered inside the anion receptor. The observed ^17^O NMR chemical shifts are in accordance with expectations arising from ^17^O NMR absolute shielding calculations and compare well with data for benchmark organic compounds containing the peroxy functional group.^23^

**Table 1:** Experimental solid-state ^17^O NMR and ADF computational results on [K_2_(DMF)_3_][^17^O_2_⊂*m*BDCA-5t-H_6_], [K_2_(DMF)_3_][C^17^O_3_⊂*m*BDCA-5t-H_6_], and related compounds

Compound		*δ*_iso_ ^*a*^/ppm	*δ*_11_/ppm	*δ*_22_/ppm	*δ*_33_/ppm	*C*_Q_/MHz	*η*_Q_/MHz
**1**	Exp	260	335	335	110	–16.6	0.0
	ADF	308	388	388	148	–17.5	0.000
**2**	Exp	170	266	194	50	7.5	0.7
	ADF	223	335	222	112	7.03	0.95
O_2_^2–^	ADF	221	398	398	–13.3	–18.66	0.000
H_2_O_2_	Exp^*b*^	180	—	—	—	–16.31	0.687
	ADF	182	383	211	–48	–16.81	0.969
Li_2_O_2_	Exp^*c*^	227	352	352	–23	–18.66	0.00

^*a*^The uncertainties in the experimental data are: *δ*_iso_ ± 2 ppm; *δ*_*ii*_ ± 10 ppm; *C*_Q_ ± 0.2 MHz; *η*_Q_ ± 0.1.

^*b*^See ref. 24.

^*c*^See ref. 25.

### The mechanism of CO_2_/peroxide driven oxidation

**Fig. 6:**
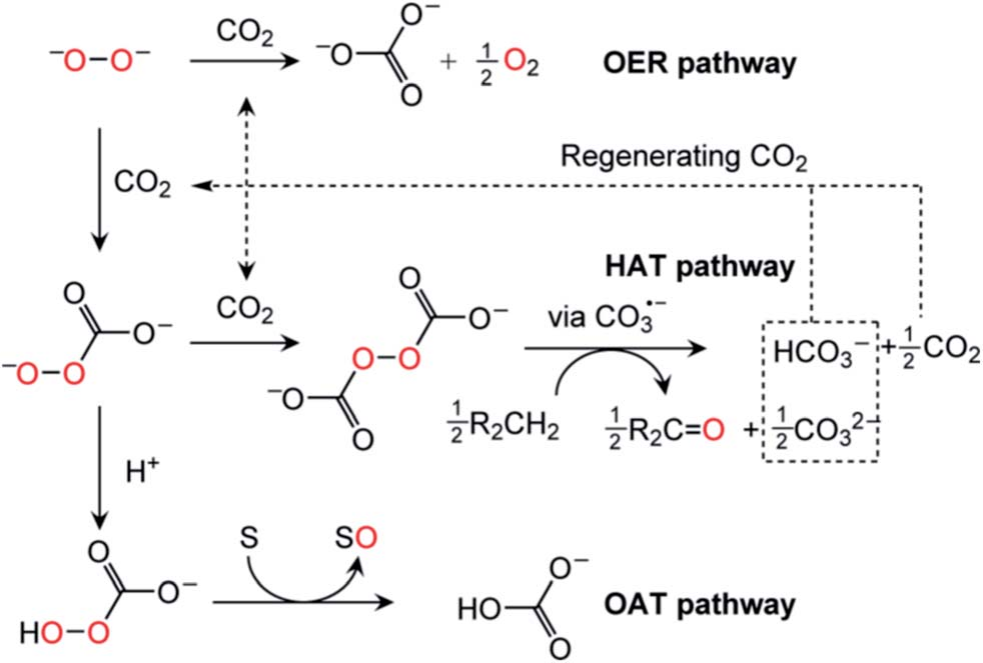
Proposed mechanistic pathways for CO_2_-mediated solvent decomposition in lithium–O_2_ batteries. OER is an “oxygen evolving reaction”, HAT is a “hydrogen atom transfer” oxidative process involving hydrogen atom abstraction by carbonate radical anion, and OAT is “oxygen atom transfer” to a substrate, S.

Having thereby established the identity of the active oxidants generated from the combination of O_2_^2–^ and CO_2_ as HOOCO_2_^–^ and ^–^O_2_COOCO_2_^–^, we next turned our attention to the mechanism of CO_2_/peroxide driven oxidation. The reaction of ^18^O-labeled **1** and CO_2_ was performed in the presence of an oxidizable substrate. Exposure of a mixture of **1**-^18^O_2_ and PPh_3_ to CO_2_ furnished ^18^OPPh_3_ as the oxidized product based on GCMS analysis.^15^ The obtained ^18^O isotope labeling data precluded the possibility of O–O bond cleavage prior to the oxygen atom transfer (OAT) reaction, as such a process would yield isotopic scrambling and result in a mixture of ^16^OPPh_3_ and ^18^OPPh_3_. Therefore, H^18^O_2_CO_2_ – with its peroxy unit intact as it was derived from the peroxide dianion – is implicated as the active species for the OAT conversion of PPh_3_ to OPPh_3_ (Fig. 6, OAT pathway). In contrast, addition of CO_2_ to a solution of **1**-^18^O_2_ in the presence of the hydrogen atom donor 9,10-dihydroanthracene (DHA) led to a statistical mixture of anthraquinone products with ^16^O and ^18^O incorporation.^15^ The observed isotope scrambling was likely due to a sequence of H-atom abstraction/radical recombination reactions. By analogy to the behavior of organic peroxydicarbonates,^26^ symmetrical peroxydicarbonate would be expected to undergo O–O bond homolysis generating two equivalents of the reactive carbonate radical CO_3_˙^–^ (Fig. 6, hydrogen atom transfer (HAT) pathway).^27^ Quantum chemical calculations indicate that homolytic cleavage of the O–O bond in ^–^O_2_COOCO_2_^–^ is only mildly endergonic (reaction free energy +14 kcal mol^–1^). This species thus has an unusually weak O–O bond.

Homolytic cleavage of the O–O bond and generation of CO_3_˙^–^ appears to be favorable for two reasons: (i) repulsion of the negative charge due to poor solvation in organic solvents resulting in coulombic explosion^28^ and (ii) resonance stabilization of the unpaired electron of the carbonate radical anion over the carbonate π system. Carbonate radicals have been generated previously *via* laser photolysis of aqueous persulfate in the presence of bicarbonate.^29^ Carbonate radicals have been implicated in guanine oxidation^27^ and are also believed to be formed upon treatment of peroxynitrite (ONOO^–^) with CO_2_, and in that case generate nitrogen dioxide as a byproduct.^27,30,31^ Furthermore, in the manganese-catalyzed oxidation of amino acids by H_2_O_2_, the formation of reactive oxygen species only occurred when HCO_3_^–^ buffer was used.^32,33^ Carbonate radicals generated in lithium–oxygen batteries can then engage in HAT reactions with solvents containing weak C–H bonds, driven by the high O–H bond strength (BDE ≅ 107 kcal mol^–1^) of the bicarbonate that is formed.^27^ Accordingly, we suggest that for stability under lithium–O_2_ cell cycling conditions, an organic solvent/electrolyte should have no C–H bonds of BDE ≅ 107 kcal mol^–1^ or less.

**Fig. 7:**
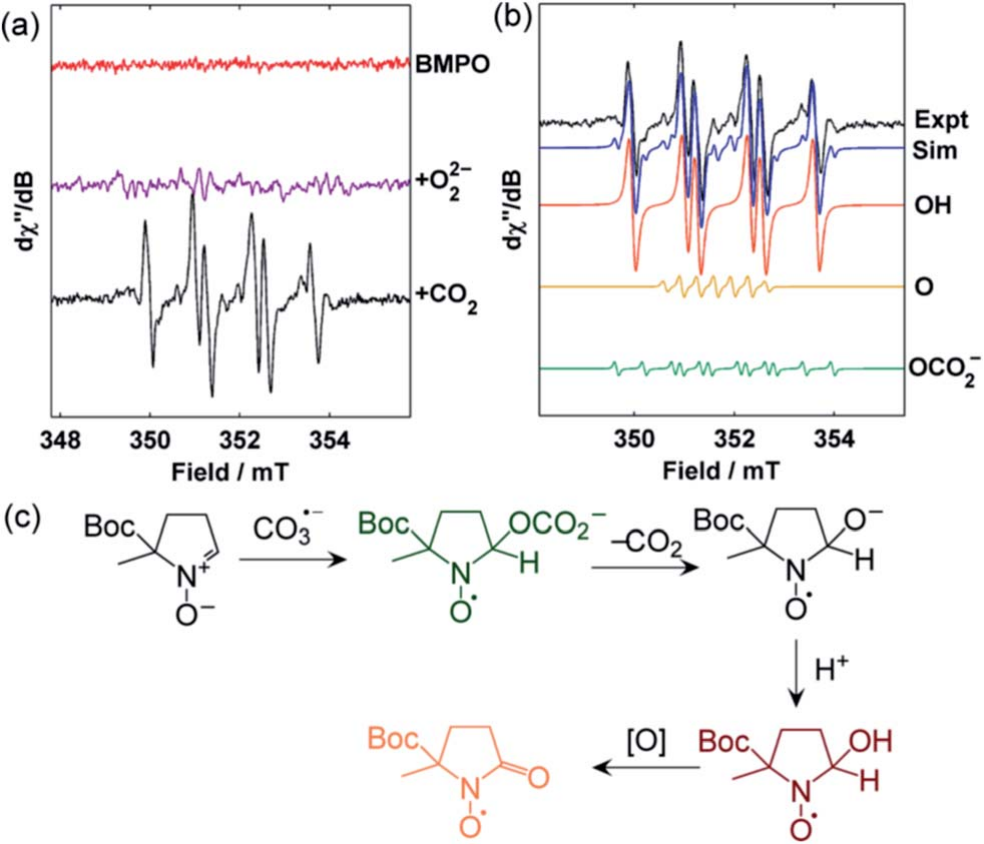
(a) X-Band EPR spectra of: pristine BMPO in DMF (red), BMPO + **1** without adding CO_2_ in DMF (purple), and exposure of BMPO + **1** to CO_2_ in DMF (black). (b) Simulation of the EPR spectra of BMPO + **1** + CO_2_ in DMF by linear combination of the contribution from: [BMPO–OCO_2_]˙^–^ (green), [BMPO–OH]˙ (red), and [BMPO–O]˙ (yellow). (c) Formation of [BMPO–O]˙ from [BMPO–OH]˙ and an oxidant “[O]”.

To experimentally confirm the generation of CO_3_˙^–^, **1** was treated with CO_2_ in the presence of the spin trap 5-*tert*-butoxycarbonyl-5-methyl-1-pyrroline-N-oxide (BMPO), and the reaction was monitored by EPR spectroscopy.^34^ Upon addition of the CO_2_, signals for the hydroxyl adduct [BMPO–OH]˙ together with small quantities (*ca.* 5%) of an unidentified spin-trap adduct suspected to be [BMPO–OCO_2_]˙^–^ were observed within seconds (Fig. 7). Formation of [BMPO–OH]˙ is proposed to occur *via* a rapid reaction between the chemically generated CO_3_˙^–^ and BMPO, initially yielding [BMPO–OCO_2_]˙^–^, followed by decarboxylation and protonation. The proton source under these conditions could be the anion receptor *m*BDCA-5t-H_6_ (Fig. 7C). This sequence is directly along the lines proposed for the related spin trap DMPO under exposure to carbonate radicals.^35,36^ On longer timescales, [BMPO–OH]˙ was further oxidized to [BMPO–O]˙ and other unidentified decomposition products.^34^

### Activation of solid Li_2_O_2_ with CO_2_ in aprotic organic media

**Fig. 8:**
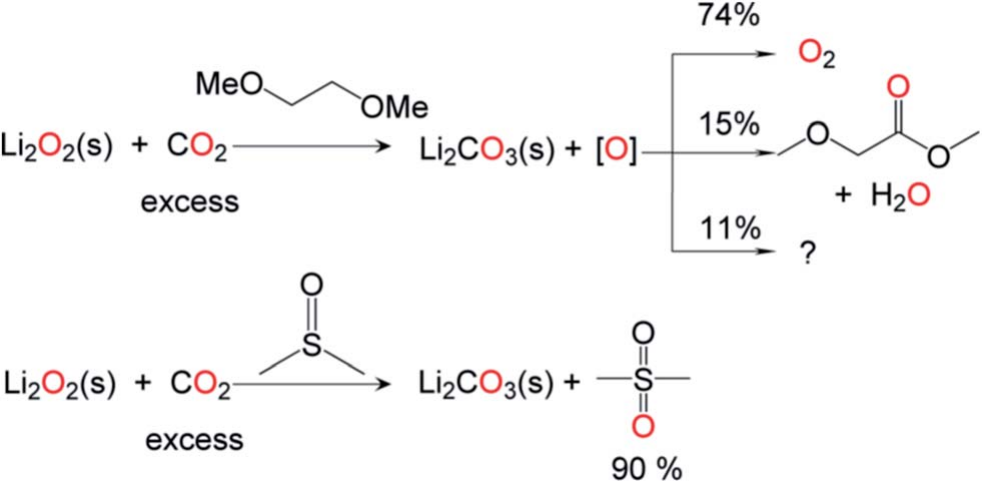
CO_2_-mediated oxidation of organic solvents by Li_2_O_2_. Addition of excess CO_2_ to solid Li_2_O_2_ in the organic solvent 1,2-dimethoxyethane (DME) generates an oxidizing equivalent “O”, which converts to O_2_ (74%) and methyl methoxyacetate (15%) with the remainder unidentified. A similar reaction performed in dimethylsulfoxide (DMSO) generated dimethylsulfone (DMSO_2_) in a 90% yield.

To examine the effect of CO_2_ on the oxidative power of Li_2_O_2_ under conditions relevant to the charging of lithium–air cells, commercially available solid Li_2_O_2_ was exposed to CO_2_ (1 atm, 25 °C, 48 h) in 1,2-dimethoxyethane (DME). In contrast to the results from the control experiments carried out similarly but in the absence of CO_2_, substantial amounts of methyl methoxyacetate were identified among the products of DME oxidation (Fig. 8). Approximately 51% of the Li_2_O_2_ was consumed, and quantitative conversion of the consumed Li_2_O_2_ to Li_2_CO_3_ (based upon lithium) was observed by ^13^C NMR spectroscopy and total inorganic carbonate (TIC) analysis.^15^ The consumed peroxide must generate an oxidizing equivalent; 74% was identified as evolved O_2_ and 15% as methyl methoxyacetate (Fig. 8), with the remainder unidentified. We also introduced solid Li_2_O_2_ into neat DMSO under a CO_2_ atmosphere (1 atm, 25 °C, 48 h), given the reported use of DMSO in lithium–O_2_ cells.^37^ More than 90% of the Li_2_O_2_ consumed participated in the conversion of DMSO to DMSO_2_ (Fig. 8). Viewed in the context of cycling lithium–O_2_ cells, the rate of CO_2_-induced solvent decomposition in bona fide lithium–air cells is perhaps lower than that observed in the current study due to the difference in CO_2_ partial pressures. Nonetheless, considering the low cycling rate and long cycling time of a typical lithium–air battery,^38^ our findings highlight that extensive oxidative degradation of the electrolyte in a cell will occur during cell cycling even when a small amount of CO_2_ is introduced or otherwise generated in the system.^39^ During cell cycling, CO_2_ is generated at the surface of lithium peroxide-impregnated carbon electrodes,^39^ leading us to speculate that the proposed chemistry (Fig. 8) should be expected to occur on a polarized electrode/electrolyte interface as well. It should be noted that while commercial Li_2_O_2_ was used in the present study, it is conceivable that the varied morphologies of electrochemically generated Li_2_O_2_ may react with CO_2_ at different rates. Due to the preponderance of conditions which result in varied Li_2_O_2_ crystallinity, size and surface structure,^40,41^ commercial Li_2_O_2_ was chosen as an ideal benchmark reactant with CO_2_.

A recent publication reported that when present in a charging lithium–air cell (>3.5 V *vs.* Li^+^/Li), the secondary amine 2,2,6,6-tetramethyl-4-piperidone (4-oxo-TEMP) was converted to the oxyl amine radical 4-oxo-TEMPO, as confirmed by EPR spectroscopy. 4-oxo-TEMP has been used in the past as a trap for singlet oxygen, leading the authors to propose that singlet oxygen was responsible for the observed conversion.^10^ An alternative explanation for the production of 4-oxo-TEMPO involves the oxidants being generated by activating Li_2_O_2_ with CO_2_, considering that the onset potential of CO_2_ formation in a typical lithium–air cell is also 3.5 V.^42^ Accordingly, we found that exposing Li_2_O_2_ to CO_2_ (1 atm, 25 °C, 24 h) in the same solvent and electrolyte as described in the literature, but without the application of an electrode potential, also resulted in the formation of 4-oxo-TEMPO. Of the oxidizing equivalents generated during the transformation of Li_2_O_2_ to Li_2_CO_3_, *ca.* 15% were incorporated into the 4-oxo-TEMPO reaction product based on EPR spin quantification. These experiments suggest that the oxidation of 4-oxo-TEMP is most likely due to CO_2_/peroxide-derived oxidants as opposed to singlet oxygen formation or at the very least that 4-oxo-TEMP is not a selective probe for singlet oxygen in Li–O_2_ cells under conditions of CO_2_ availability.

## Conclusions

While previous studies on lithium–O_2_ batteries have attributed the low cycling number and capacity fading to singlet oxygen^10^ and superoxide,^4–7^ it is now clear that CO_2_/peroxide-derived oxidants are responsible for carbonate formation by way of the active oxidants HOOCO_2_^–^ and CO_3_˙^–^ *via* ^–^O_2_COOCO_2_^–^. Since prototypical lithium–air cells (ether electrolyte, carbon cathode) lose 5–7% of their capacity to parasitic CO_2_ formation per complete cycle^39^ and have a typical cycling number of *ca.* 50, the resulting CO_2_/peroxide dianion-derived oxidants were expected to cause organic electrolyte degradation. This oxidative degradation may occur both during discharge through reaction of the peroxide dianion with CO_2_ and during recharge through electrochemical oxidation of carbonate initially generating the carbonate radical (CO_3_˙^–^). It has been established that recharging a lithium–O_2_ battery regenerates CO_2_ from Li_2_CO_3_, however identification of the mechanism and product(s) of electrochemical Li_2_CO_3_ degradation have been unclear.^2^ Our studies provide evidence for a mechanistic pathway by which carbonate radical anions, when generated, engage in C–H abstraction from the solvent (C–H bond ≅ 107 kcal mol^–1^, or less)^27^ and lead to solvent degradation and reformation of CO_2_ (Fig. 6). The regenerated CO_2_ sets in motion a decomposition cycle, therefore if even a small percentage of the total Li_2_O_2_ is converted to CO_2_, extensive oxidative degradation of the electrolyte in a cell will occur over the course of many cycles. If CO_2_ cannot be excluded from these systems then it is critical that the electrolyte and other cell components are invulnerable to reactive CO_2_/peroxide-derived oxidants if the full potential of rechargeable lithium–O_2_ battery systems is to be realized.
