# Supplementary material for: On the incompatibility of lithium–O2 battery technology with CO2
Source: Chem Sci. 2017 Jun 20;8(9):6117–22. doi: 10.1039/c7sc01230f (PMC5625616; doi:10.1039/c7sc01230f)
Supplement: Supplementary file 1 [file SC-009-C7SC01230F-s001.pdf]

Electronic Supplementary Material (ESI) for Chemical Science.
This journal is © The Royal Society of Chemistry 2017

Electronic Supplementary Information for On the incompatibility of lithium-O_2 battery technology with CO_2

Shiyu Zhang,[†,‡] Matthew J. Nava,[†,‡] Gary K. Chow,[¶] Nazario Lopez,[†,§] Gang Wu,[‖] David R. Britt,[¶] Daniel G. Nocera,[*,⊥] and Christopher C. Cummins[*,†]

†*Department of Chemistry, Massachusetts Institute of Technology, 77 Massachusetts Avenue, Cambridge, MA 02139*

‡*These authors contributed equally*

¶*Department of Chemistry, University of California, Davis, One Shields Avenue, Davis, CA 95616*

§*Current Address: Centro de Investigaciones Químicas, IICBA, Universidad Autonoma del Estado de Morelos, Avenida Universidad 1001, Colonia Chamilpa, C.P. 62209, Cuernavaca, Morelos Mexico*

‖*Department of Chemistry, Queens University, 90 Bader Lane, Kingston, Ontario K7L3N6, Canada*

⊥*Department of Chemistry and Chemical Biology, Harvard University, 12 Oxford Street, Cambridge, MA 02138*

E-mail: dnocera@fas.harvard.edu; ccummins@mit.edu

Contents

1 Materials and methods **S5**

2 Reactivity of [K(18-crown-6)]$_2$[O$_2$⊂mBDCA-5t-H$_6$] with CO_2 S6

2.1 Treatment of [K(18-crown-6)]$_2$[O$_2$⊂mBDCA-5t-H$_6$] with CO_2 in DMF-d_7 . . . . S6

2.2 Treatment of [K(18-crown-6)]$_2$[O$_2$⊂mBDCA-5t-H$_6$] with CO_2 in DMSO-d_6 . . . . S8

2.3 Treatment of [K(18-crown-6)]$_2$[O$_2$⊂mBDCA-5t-H$_6$] with CO_2 in the presence of PPh_3 . . . . S9

2.4 Treatment of [K(18-crown-6)]$_2$[O$_2$⊂mBDCA-5t-H$_6$] with CO_2 in the presence of 4-methoxythioanisole S12

2.5 Treatment of [K(18-crown-6)]$_2$[O$_2$⊂mBDCA-5t-H$_6$] with CO_2 in the presence of 9, 10-dihydroanthracene (DHA) S13

3 Reactivity of Li_2O_2 with CO_2 in organic solvents S17

3.1 Treatment of Li_2O_2 with CO_2 (1 atm) in DME S17

3.2 Treatment of Li_2O_2 with CO_2 (1 atm) in DMSO S20

3.3 Treatment of Li_2O_2 with 2,2,6,6-tetramethylpiperidone (4-oxo-TEMP) under CO_2 (1 atm) S21

3.4 Li_2O_2 quantification protocol S24

3.5 Li_2CO_3 quantification protocol S27

4 Variable temperature NMR spectroscopy studies S28

4.1 ^{13}C and ^{1}H NMR analysis of the mixture resulting from the reaction of [K(18-crown-6)]$_2$[O$_2$⊂mBDCA-5t-H$_6$] with $^{13}CO_2$ S28

4.2 ^{17}O NMR analysis of the mixture resulting from the reaction of [K(18-crown-6)]$_2$[^{17}O$_2$⊂mBDCA-5t-H$_6$] with $^{13}CO_2$ S30

4.3 Monitoring the reaction of [K(18-crown-6)]$_2$[O$_2$⊂mBDCA-5t-H$_6$] and $^{13}CO_2$ in the presence of PPh_3 . . . . S31

4.4 Generation of hydroperoxycarbonate $HOO^{13}CO_2^-$ from [PPN][$H^{13}CO_3$] and H_2O_2 S33

4.5 Generation of peroxydicarbonate $^-O_2{}^{13}COO^{13}CO_2^-$ from bis(trimethylsilyl) peroxide (TMSOOTMS), potassium *tert*-butoxide (KO*t*Bu) and $^{13}CO_2$ in DMF S35

4.5.1 Control experiment A S36
4.5.2 Control experiment B S37
4.6 Attempt to generate unsymmetrical peroxydicarbonate *in situ* from KO_2 and $^{13}CO_2$ S38

5 Synthesis and characterization of monodeprotonated anion receptor [*m*BDCA-5t-H_5]$^-$ S39
5.1 Preparation of [K(18-crown-6)][*m*BDCA-5t-H_5] S39
5.2 Preparation of [K(Kryptofix 222)][*m*BDCA-5t-H_5] S42

6 Gas chromatography and mass spectrometry (GCMS) studies S46
6.1 Instrument configuration S46
6.2 GCMS of the $OPPh_3$ produced from the reaction of [K(18-crown-6)]$_2$[$^{18}O_2\subset m$BDCA-5t-H_6] and PPh_3 under CO_2 (1 atm) S46
6.3 GCMS of the anthraquinone produced from the reaction of [K(18-crown-6)]$_2$[$^{18}O_2\subset m$BDCA-5t-H_6] with DHA under CO_2 (1 atm) S47

7 EPR studies S49
7.1 Materials and methods S49
7.2 EPR spectroscopy S50
7.3 Mass spectrometry S50
7.4 EPR sample preparation S50
7.5 EPR standard preparation S51

8 Solid state reactivity of [K(18-crown-6)]$_2$[$O_2\subset m$BDCA-5t-H_6] with CO_2 S54
8.1 Experimental solid-state ^{17}O NMR details S55
8.2 Treatment of solid [K(18-crown-6)]$_2$[$O_2\subset m$BDCA-5t-H_6] with CO_2 followed by dissolution in a solution containing PPh_3 . . . . S56
8.3 DRIFTS measurements of [K(18-crown-6)]$_2$[$O_2\subset m$BDCA-5t-H_6] treated with CO_2 in the solid state S58

8.4 Variable temperature RAMAN spectroscopy of [K(18-crown-6)]$_2$[$O_2 \subset m$BDCA-5t-H_6] exposed to CO_2 in the solid state . . . S59
8.5 Gas Chromatography (GC) of the reaction vessel headspace after exposure of [K(18-crown-6)]$_2$[$O_2 \subset m$BDCA-5t-H_6] to CO_2 . . . S61

9 Ab initio calculation details **S62**
9.1 Computational Methodology . . . S62
9.2 ^{13}C NMR calculation . . . S63
9.3 ^{17}O NMR calculation . . . S64
9.4 Determination of the O–O Bond Dissociation Enthalpy (BDE) in $^{-}O_2COOCO_2^{-}$. S65

10 Appendix **S66**
10.1 Coordinates of peroxycarbonate optimized at the B3LYP/6-311G++(2d,2p) level of theory used for GIAO NMR calculations . . . S66
10.2 Coordinates of hydroperoxycarbonate optimized at the B3LYP/6-311G++(2d,2p) level of theory used for GIAO NMR calculations . . . S67
10.3 Coordinates of symmetrical peroxydicarbonate optimized at the B3LYP/6-311G++(2d,2p) level of theory used for GIAO NMR calculations . . . S67
10.4 Coordinates of unsymmetrical peroxydicarbonate optimized at the B3LYP/6-311G++(2d,2p) level of theory used for GIAO NMR calculations . . . S68
10.5 Coordinates of symmetrical peroxydicarbonate optimized at the MP2/6-311G++(2d,2p) level of theory used for BDE calculations . . . S68
10.6 Coordinates of the carbonate radical anion optimized at the MP2/6-311G++(2d,2p) level of theory used for BDE calculations . . . S69

References **S69**

1 Materials and methods

All manipulations were carried out either in a Vacuum Atmospheres model MO-40M glovebox under an atmosphere of N_2 or using standard Schlenk techniques. ^{1}H, ^{13}C, ^{31}P, and ^{17}O NMR spectra were recorded on a Varian 500 MHz spectrometer and were externally referenced to the NMR residual solvent peaks. ESI-MS data were obtained on a Waters Q-TOF micro mass spectrometer using a source temperature of 100 °C and a desolvation temperature of 150 °C. ESI-MS samples were run in neat DMF at concentrations < 1 μM and the data were processed using the program mMass Version 5.4.1.0.E. Elemental analyses were performed by Robertson Microlit Laboratories (http://www.robertson-microlit.com/). O_2 quantification was performed with Gas Chromatography (GC) equipped with a thermal conductivity detector (multiple gas analyzer #3, SRI Instrument). Unless otherwise noted, all solvents were degassed and dried using a Glass Contour Solvent Purification System built by SG Water USA, LLC. After purification, all solvents were stored under an atmosphere of N_2 over 4 Å molecular sieves. Molecular sieves (4 Å) were dried at 50 mTorr overnight at a temperature above 200 °C. DMSO-d_6 (Cambridge Isotope Labs) was dried over CaH_2 and vacuum transferred onto 4 Å molecular sieves. DMF-d_7 (Cambridge Isotope Labs) was transferred from ampoules to a scintillation vial (20 mL) with 4 Å molecular sieves and allowed to stand for at least 3 days before use. Celite 435 (EMD Chemicals). All glassware were oven dried at 220 °C prior to use. $^{17}O_2$ (70% ^{17}O) was purchased from Cambridge Isotope Labs and used as received. Carbon dioxide (CO_2, 99.995%) was purchased from Airgas and used as received. Bis(trimethylsilyl) peroxide (TMSOOTMS) was purchased from TCI Ameria and used as received. $^{18}O_2$ (97% ^{18}O), $^{13}CO_2$ (99%^{13}C, <5% ^{18}O), and all other starting materials were purchased from Sigma-Aldrich (St. Louis, MO) and used without further purification. [K(18-crown-6)]$_2$[$O_2$$\subset$*m*BDCA-5t-$H_6$], [K(18-crown-6)]$_2$[$^{17}O_2$$\subset$*m*BDCA-5t-$H_6$], [$K_2DMF_3$][$^{17}O_2$$\subset$*m*BDCA-5t-$H_6$] and [K(18-crown-6)]$_2$[$^{18}O_2$$\subset$*m*BDCA-5t-$H_6$] were prepared according to literature procedures (see reference 11 and 12 in main text).

2 Reactivity of [K(18-crown-6)]$_2$[O$_2$$\subset$*m*BDCA-5t-H$_6$] with CO_2

To probe the fate of the missing oxygen atom, the reaction of CO_2 and [K(18-crown-6)]$_2$[O$_2$$\subset$*m*BDCA-5t-H$_6$] was performed in the presence of oxidizable substrates, such as triphenylphosphine (PPh_3), methoxythioanisole, and 9, 10-dihydroanthracene (DHA). While [K(18-crown-6)]$_2$[O$_2$$\subset$*m*BDCA-5t-H$_6$] on its own is unreactive towards these substrate at 25 °C, exposing a mixture of [K(18-crown-6)]$_2$[O$_2$$\subset$*m*BDCA-5t-H$_6$] and organic substrates to CO_2 (1 atm, 25 °C) resulted in rapid formation of [K(18-crown-6)]$_2$[CO$_3$$\subset$*m*BDCA-5t-H$_6$] and oxidized product: triphenylphosphine oxide ($OPPh_3$, 90%), 1-(methylsulfinyl)-4-methoxybenzene (61%) and anthraquinone (18-72%), respectively.

2.1 Treatment of [K(18-crown-6)]$_2$[O$_2$$\subset$*m*BDCA-5t-H$_6$] with CO_2 in DMF-d_7

[K(18-crown-6)]$_2$[O$_2$$\subset$*m*BDCA-5t-H$_6$] (14.7 mg, 0.00987 mmol) was dissolved in DMF-d_7 (*ca*. 0.8 mL) and the solution was transferred to an NMR tube equipped with a septum (Figure S1). CO_2 (1.00 mL, 1 atm, 25 °C, 0.0410 mmol, 4.15 equiv) was added to the NMR tube using a gas tight syringe equipped with a ball valve. The yellow color of [K(18-crown-6)]$_2$[O$_2$$\subset$*m*BDCA-5t-H$_6$] quickly bleached to afford a colorless homogeneous solution. ^{1}H NMR analysis of the reaction mixture shows formation of [K(18-crown-6)]$_2$[CO$_3$$\subset$*m*BDCA-5t-H$_6$] in 77% yield using 18-crown-6 as an internal standard. ^{1}H NMR (DMF-d_7, 500 MHz, 21 °C, ppm) δ, Figure S2: 12.57 (s, 6H), 11.22 (s, 3H), 8.18 (s, 6H), 3.61 (s, 48H), 2.50 (br, 12H), 1.33 (s, 27H).

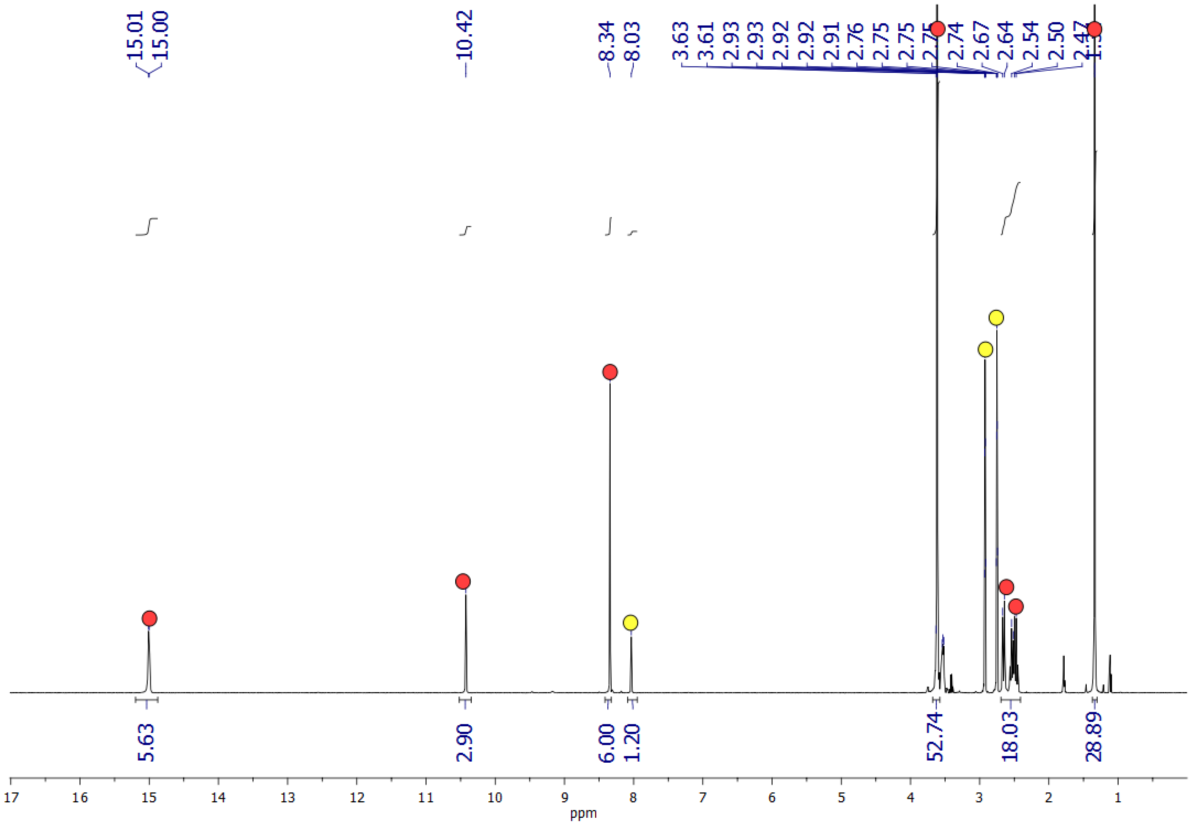

Figure S1: ^{1}H NMR (DMF-d_7, 500 MHz, 21 °C) spectrum of $[K(18\text{-crown-}6)]_2[O_2\subset m\text{BDCA-5t-}H_6]$. The DMF peaks are indicated by yellow circles and the peroxide cryptate by red circles.

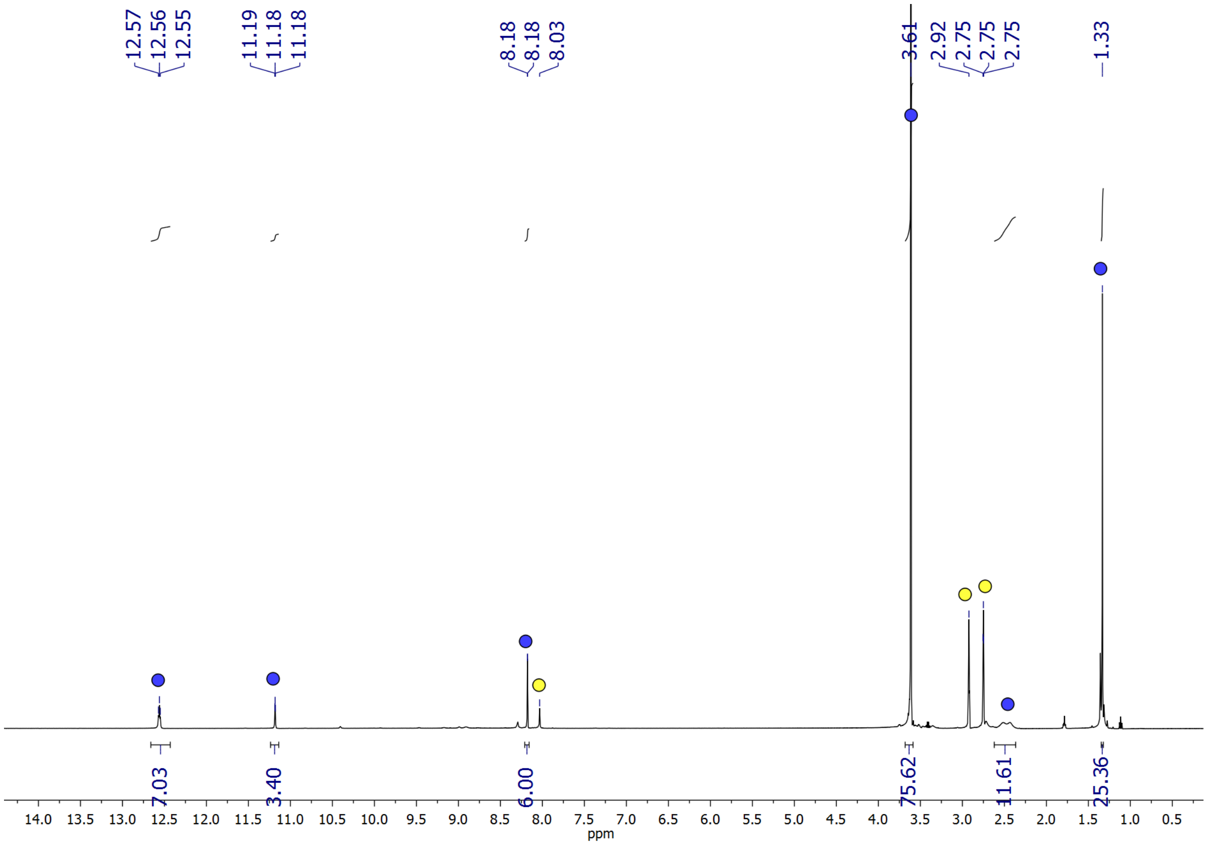

Figure S2: ^{1}H NMR (DMF-d_7, 500 MHz, 21 °C) spectrum of the mixture formed from the reaction of [K(18-crown-6)]$_2$[O_2⊂*m*BDCA-5t-H_6] with CO_2. The DMF peaks are indicated by yellow circles, and the carbonate cryptate by blue circles.

2.2 Treatment of [K(18-crown-6)]$_2$[O_2⊂*m*BDCA-5t-H_6] with CO_2 in DMSO-d_6

[K(18-crown-6)]$_2$[O_2⊂*m*BDCA-5t-H_6] (115.3 mg, 0.0774 mmol) was dissolved in DMSO-d_6 (*ca.* 5 mL) and the solution was transferred a Schlenk flask (50 mL). The flask was evacuated and the headspace was backfilled with CO_2 (1 atm), upon which the color of the solution changed from bright yellow to colorless concomitant with the formation of a white precipitate. The resulting suspension was stirred at 25 °C for 4 h. The reaction vessel was degassed and brought back into the glovebox. The resulting white precipitate was collected, washed with diethyl ether (20 mL), then dried under reduced pressure to afford 13.9 mg solid. The ^{1}H NMR spectrum of the white solid is in agreement with that for the free anion receptor *m*BDCA-5t-H_6. ^{1}H NMR (DMF-d_7, 500

MHz, 21 °C, ppm) δ, Figure S3: 8.87 (s, 6H), 8.29 (s, 6H), 8.17 (s, 6H), 3.35 (s, 6H), 2.72 (s, 6H), 1.36 (s, 27H). Formation of $[K(18\text{-crown-}6)]_2[CO_3{\subset}m\text{BDCA-5t-}H_6]$ was also observed.

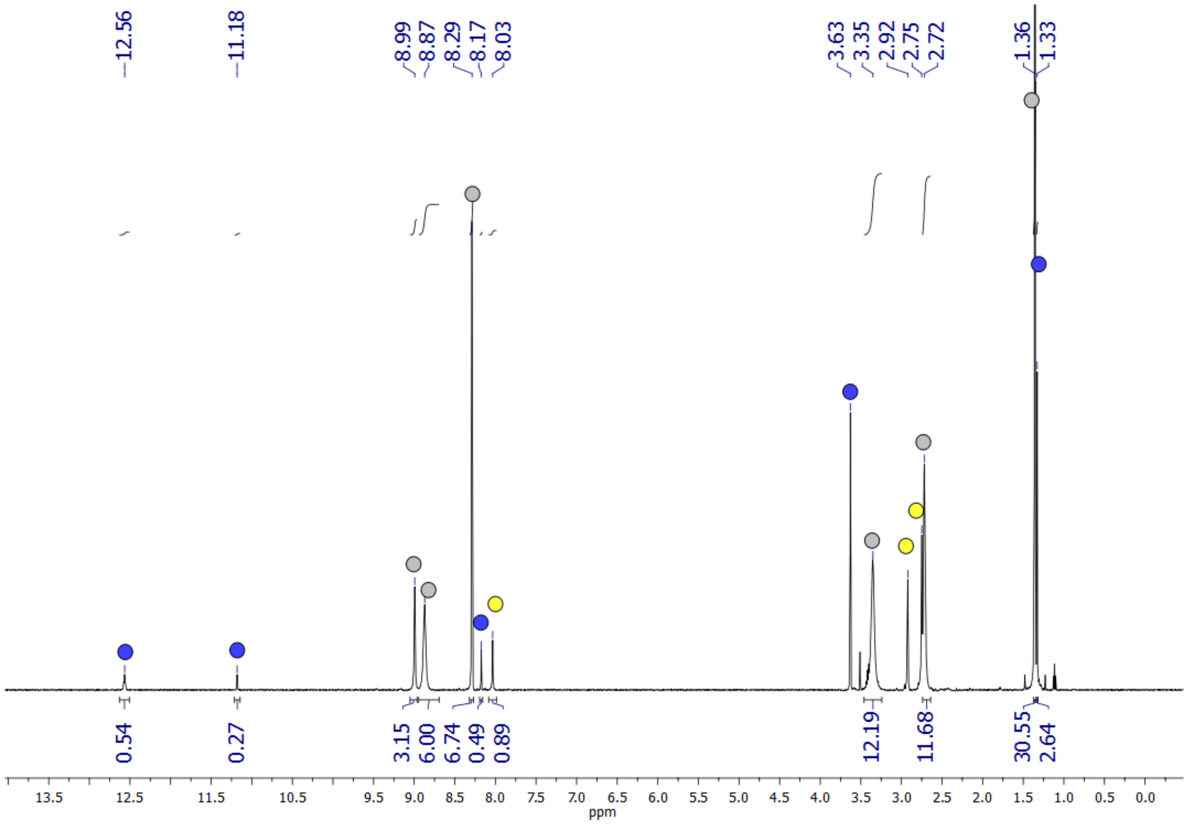

Figure S3: ^{1}H NMR (DMF-d_7, 500 MHz, 21 °C) spectrum of the colorless precipitate isolated from the reaction of $[K(18\text{-crown-}6)]_2[O_2{\subset}m\text{BDCA-5t-}H_6]$ with CO_2. The DMF peaks are indicated by yellow circles, the free anion receptor by grey circles and the carbonate cryptate by blue circles.

2.3 Treatment of $[K(18\text{-crown-}6)]_2[O_2{\subset}m\text{BDCA-5t-}H_6]$ with CO_2 in the presence of PPh_3

$[K(18\text{-crown-}6)]_2[O_2{\subset}m\text{BDCA-5t-}H_6]$ (17.7 mg, 0.0119 mmol) and PPh_3 (7.1 mg, 0.027 mmol, 2.26 equiv) were dissolved in DMSO-d_6 (*ca.* 0.8 mL) and the resulting solution was transferred to an NMR tube equipped with a septum. Carbon dioxide (1.00 mL, 1 atm, 25 °C, 0.0410 mmol, 3.45 equiv) was added to the NMR tube at 25 °C using a gas tight syringe equipped with a ball valve. The yellow color of $[K(18\text{-crown-}6)]_2[O_2{\subset}m\text{BDCA-5t-}H_6]$ quickly bleached and afforded a colorless homogeneous solution. ^{1}H NMR analysis of the reaction mixture shows [K(18-crown-

6)]$_2$[CO_3⊂*m*BDCA-5t-H_6] and $OPPh_3$ formed in 87% and 90% spectroscopic yield using 18-crown-6 as an internal standard. ^{1}H NMR (DMF-d_7, 500 MHz, 21 °C, ppm) δ, Figure S4: 12.56 (s, 6H), 11.19 (s, 3H), 8.18 (s, 6H), 7.73 (s, 6H, $OPPh_3$) 7.67 (s, 3H, $OPPh_3$) 7.60 (s, 6H, $OPPh_3$), 3.61 (s, 48H), 2.50 (br, 12H), 1.33 (s, 27H). ^{31}P{^{1}H} NMR (DMF-d_7, 203 MHz, 21 °C, ppm) δ, Figure S5: 24.87 (s, 1 P). A control reaction of [K(18-crown-6)]$_2$[O_2⊂*m*BDCA-5t-H_6] with PPh_3 was conducted and indicated there was no observable reaction at 25 °C over the course of 4 hours without added CO_2 (Figure S6).

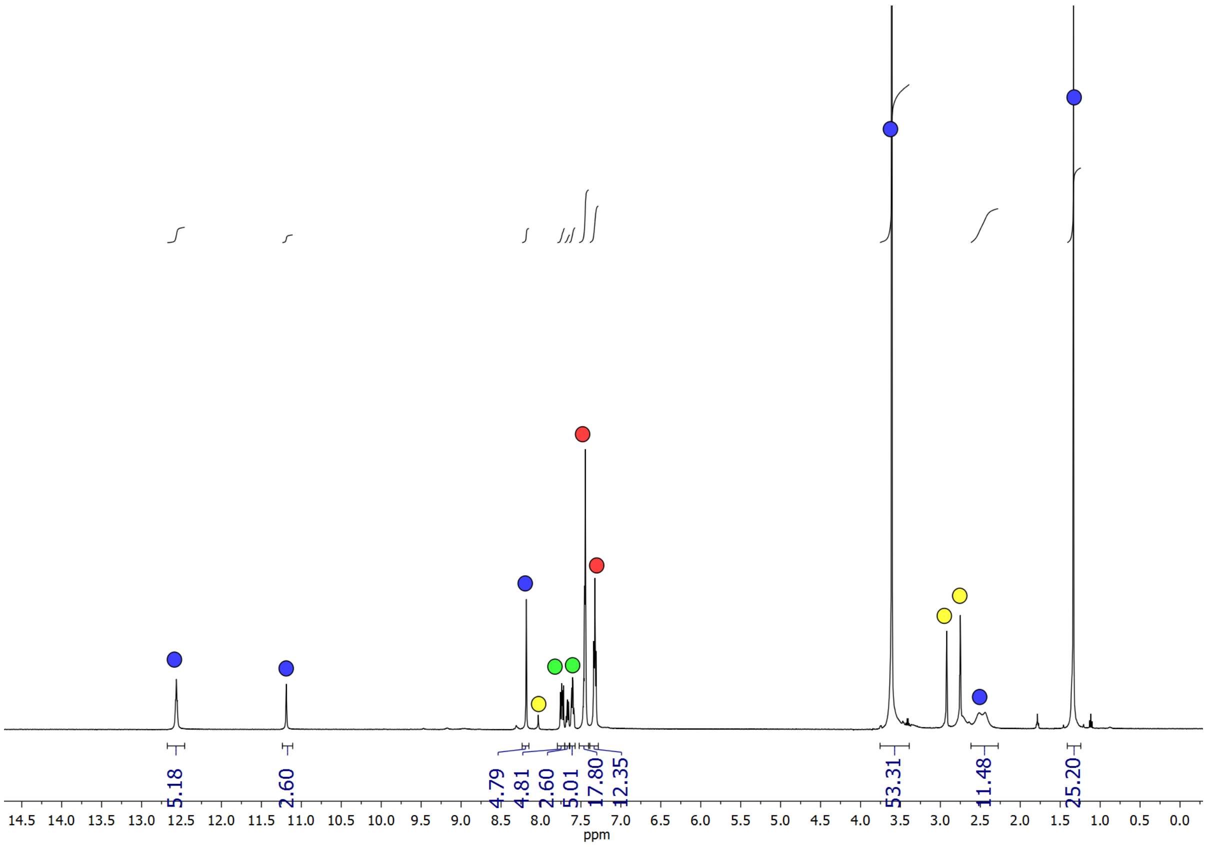

Figure S4: ^{1}H NMR (DMF-d_7, 500 MHz, 21 °C) spectrum of the mixture formed from the reaction of [K(18-crown-6)]$_2$[O_2⊂*m*BDCA-5t-H_6] with CO_2 in the presence of PPh_3. The DMF peaks are indicated by yellow circles, the carbonate cryptate by blue circles, PPh_3 by red circles, $OPPh_3$ by green circles. Addition of CO_2 to a mixture of [K(18-crown-6)]$_2$[O_2⊂*m*BDCA-5t-H_6] and PPh_3 results in instantaneous formation of carbonate cryptate and $OPPh_3$.

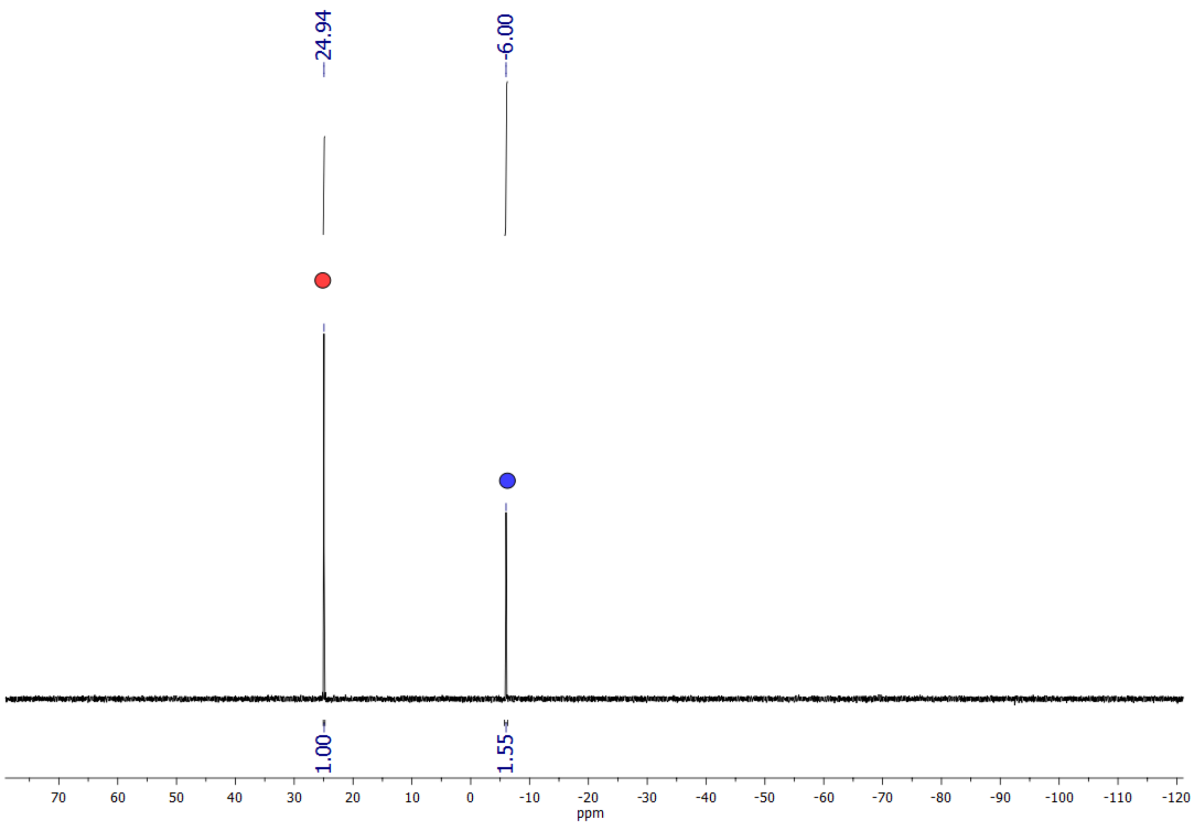

Figure S5: ^{31}P NMR (DMF-d_7, 203 MHz, 21 °C) spectrum of the mixture formed from the reaction of $[K(18\text{-crown-6})]_2[O_2{\subset}m\text{BDCA-5t-}H_6]$ with CO_2 in the presence of PPh_3. PPh_3 is indicated by a blue circle and $OPPh_3$ by a red circle.

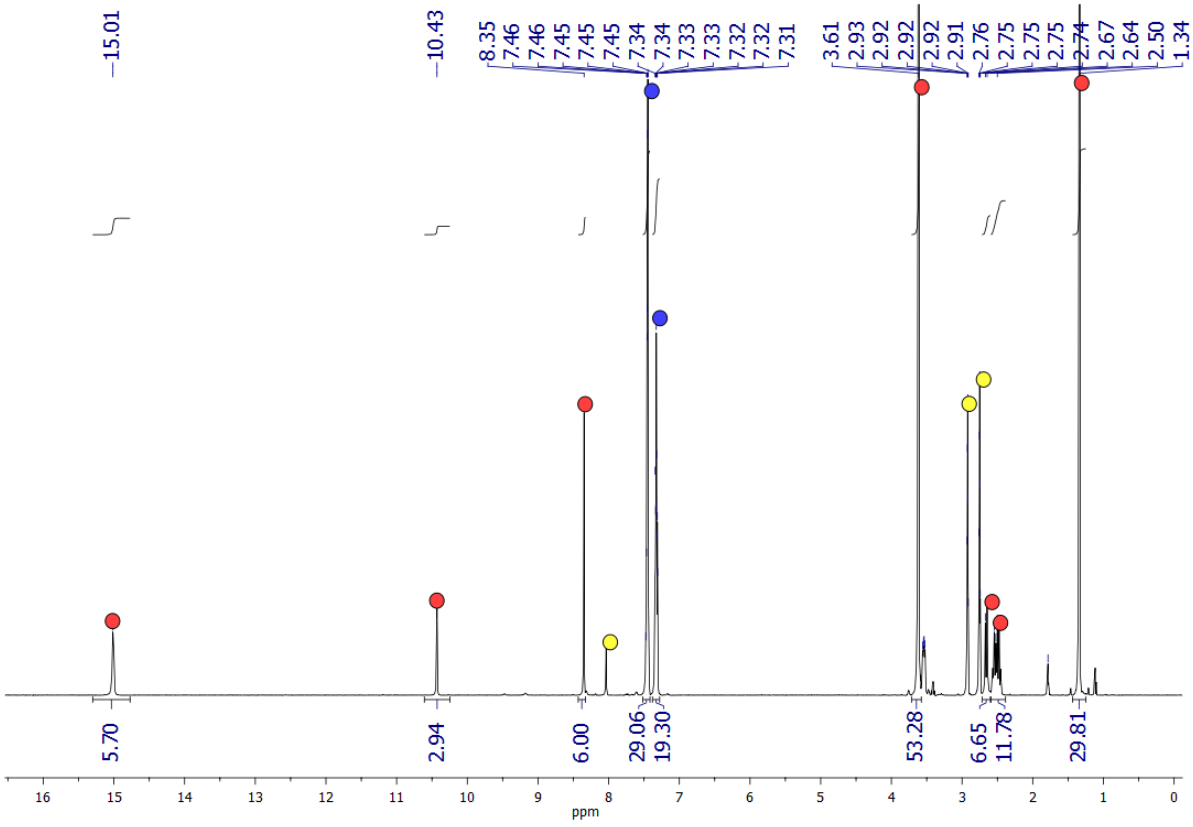

Figure S6: ^{1}H NMR (DMF-d_7, 500 MHz, 21 °C) spectrum of a mixture of [K(18-crown-6)]$_2$[$O_2\subset m$BDCA-5t-H_6] and PPh_3. The DMF peaks are indicated by yellow circles, the peroxide cryptate by red circles, and PPh_3 by blue circles. This reaction functions as a control and indicates that there was no observable reaction of [K(18-crown-6)]$_2$[$O_2\subset m$BDCA-5t-H_6] with PPh_3 at 25 °C over the course of 4 hours without added CO_2.

2.4 Treatment of [K(18-crown-6)]$_2$[$O_2\subset m$BDCA-5t-H_6] with CO_2 in the presence of 4-methoxythioanisole

[K(18-crown-6)]$_2$[$O_2\subset m$BDCA-5t-H_6] (8.3 mg, 5.6 μmol) and 4-methoxythioanisole (38.9 mg, 0.252 mmol, 45 equiv) were dissolved in DMF (1.00 mL) and the resulting solution was transferred to a GC vial equipped with a septum. Carbon dioxide (1.00 mL, 1 atm, 25 °C, 0.0410 mmol, 7.3 equiv) was added to the GC vial using a gas tight syringe equipped with a ball valve. The yellow color of [K(18-crown-6)]$_2$[$O_2\subset m$BDCA-5t-H_6] quickly bleached and afforded a colorless homogeneous solution. After 30 minutes, all volatiles were removed under reduced pressure and the remaining solid was redissolved in DMSO-d_6 for NMR analysis. [K(18-crown-6)]$_2$[$CO_3\subset m$BDCA-

5t-H_6] and 1-(methylsulfinyl)-4-methoxybenzene formed in 91% and 61% spectroscopic yield using 18-crown-6 as an internal standard. ^{1}H NMR (DMSO-d_6, 500 MHz, 21 °C, ppm) δ, Figure S7: 12.16 (s, 6H), 10.82 (s, 3H), 8.01 (s, 6H), 7.61 (d, 2H, 1-(methylsulfinyl)-4-methoxybenzene), 7.11 (d, 2H, 1-(methylsulfinyl)-4-methoxybenzene), 3.50 (s, 48H), 1.31 (s, 27H).

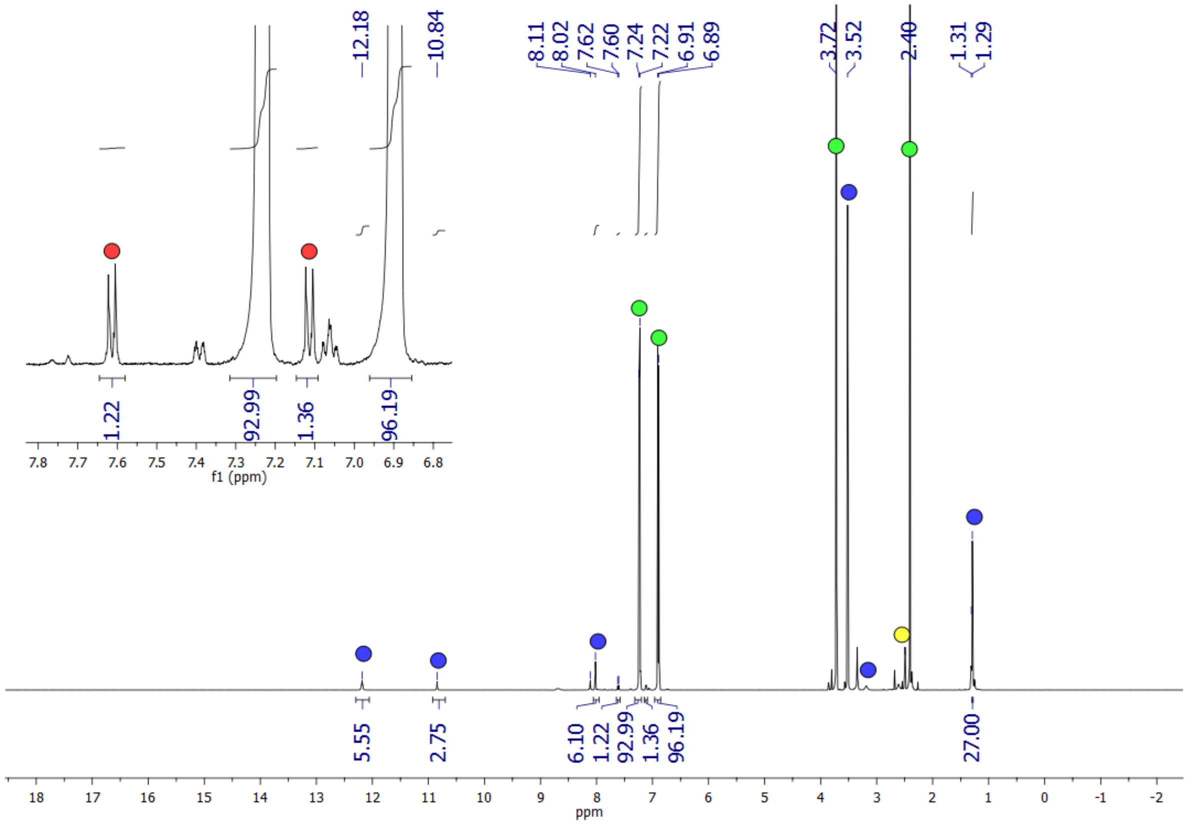

Figure S7: ^{1}H NMR (DMSO-d_6, 500 MHz, 21 °C) spectrum of the mixture formed from the reaction of [K(18-crown-6)]$_2$[$O_2 \subset m$BDCA-5t-H_6] with CO_2. The DMSO peak is indicated by a yellow circle, 1-(methylsulfinyl)-4-methoxybenzene product by red circles, the carbonate cryptate by blue circles, and starting material, 4-methoxythioanisole, by green circles.

2.5 Treatment of [K(18-crown-6)]$_2$[$O_2 \subset m$BDCA-5t-H_6] with CO_2 in the presence of 9, 10-dihydroanthracene (DHA)

A stock solution of [K(18-crown-6)]$_2$[$O_2 \subset m$BDCA-5t-H_6] (30.2 mM) was prepared by dissolving solid [K(18-crown-6)]$_2$[$O_2 \subset m$BDCA-5t-H_6] (21.0 mg, 0.0141 mmol) in DMF-d_7 (0.467 mL). A stock solution of DHA (10.2 mM) was prepared by dissolving solid DHA (10.2 mg,

0.0567 mmol) in DMF-d_7 (0.558 mL). A series of solutions with different ratios of [K(18-crown-6)]$_2$[$O_2 \subset m$BDCA-5t-H_6] to DHA were prepared as follows:

Table S1: Preparation of solutions for the reaction of [K(18-crown-6)]$_2$[$O_2 \subset m$BDCA-5t-H_6] with CO_2 in the presence of 9, 10-dihydroanthracene (DHA).

Ratio **1**:DHA	**1** (0.030 mM)	DHA (0.102 mM)	DMF
1:1	0.100 mL	0.030 mL	0.270 mL
5:1	0.100 mL	0.150 mL	0.150 mL
10:1	0.100 mL	0.300 mL	0 mL

The resulting solutions were transferred to three NMR tubes equipped with septa. The NMR tubes were removed from the glovebox and treated with CO_2 (3 mL, 1 atm, 25 °C) using a gas tight syringe equipped with a ball valve. The yellow color of [K(18-crown-6)]$_2$[$O_2 \subset m$BDCA-5t-H_6] quickly bleached to afford a colorless homogeneous solution. The NMR spectrum of each sample was taken to determine the yields of anthraquinone and [K(18-crown-6)]$_2$[$CO_3 \subset m$BDCA-5t-H_6], which are summarized in the table below. ^{1}H NMR (DMF-d_7, 500 MHz, 21 °C, ppm) δ: 12.59 (s, 6H), 11.21 (s, 3H), 8.33 (m, 4H, anthraquinone), 8.18 (s, 6H), 8.02 (m, 4H, anthraquinone), 7.73 (s, 6H) 7.67 (s, 3H) 7.60 (s, 6H), 3.61 (s, 48H), 2.48 (m, 12H), 1.33 (s, 27H).

Table S2: Yields of [K(18-crown-6)]$_2$[$CO_3 \subset m$BDCA-5t-H_6] and anthraquinone from the reaction of [K(18-crown-6)]$_2$[$O_2 \subset m$BDCA-5t-H_6] with CO_2 in the presence of 9, 10-dihydroanthracene (DHA).

Equiv. of DHA	**2** yield	anthraquinone yield
1 eq	72%	18%
5 eq	87%	55%
10 eq	88%	72%

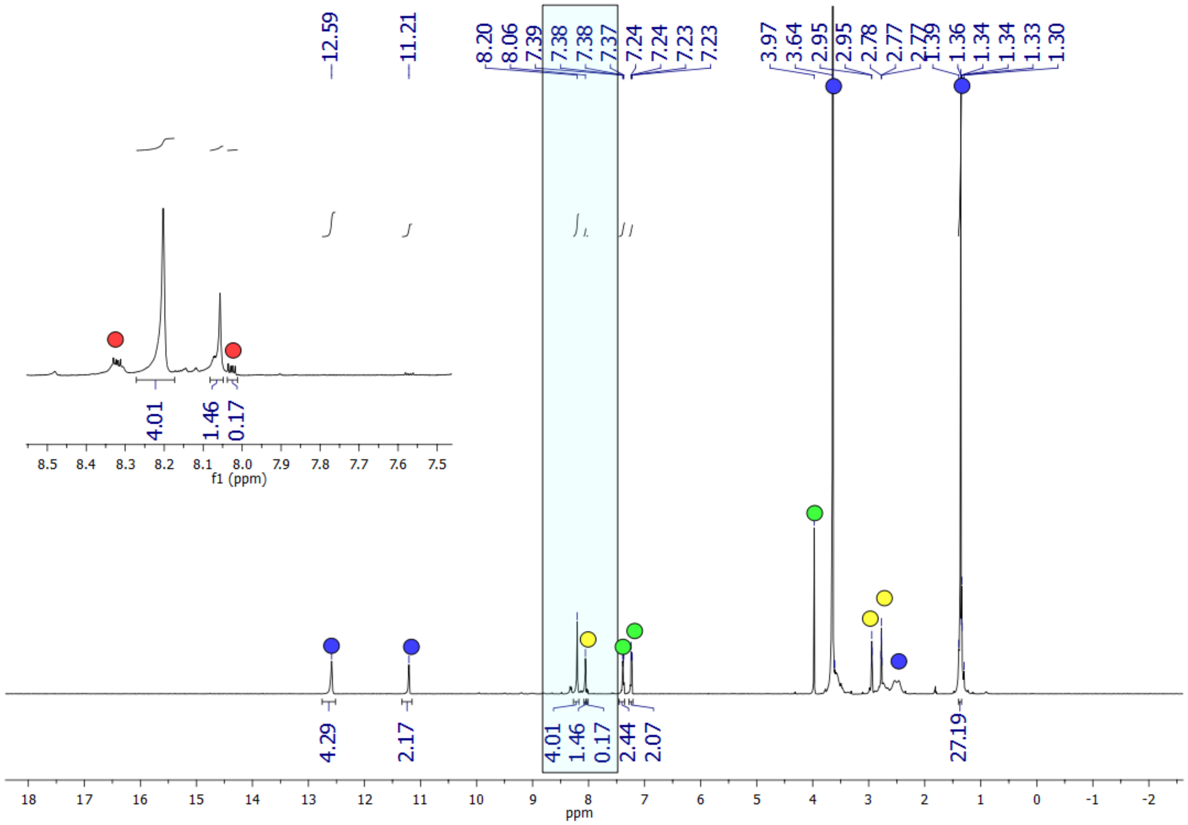

Figure S8: ^{1}H NMR (DMF-d_7, 500 MHz, 21 °C) spectrum of the mixture formed from the reaction of $[K(18\text{-crown-6})]_2[O_2{\subset}m\text{BDCA-5t-H}_6]$ with CO_2 in the presence of 1 eq DHA. The DMF peaks are indicated by yellow circles, anthraquinone by red circles, DHA by green circles, and the carbonate cryptate by blue circles.

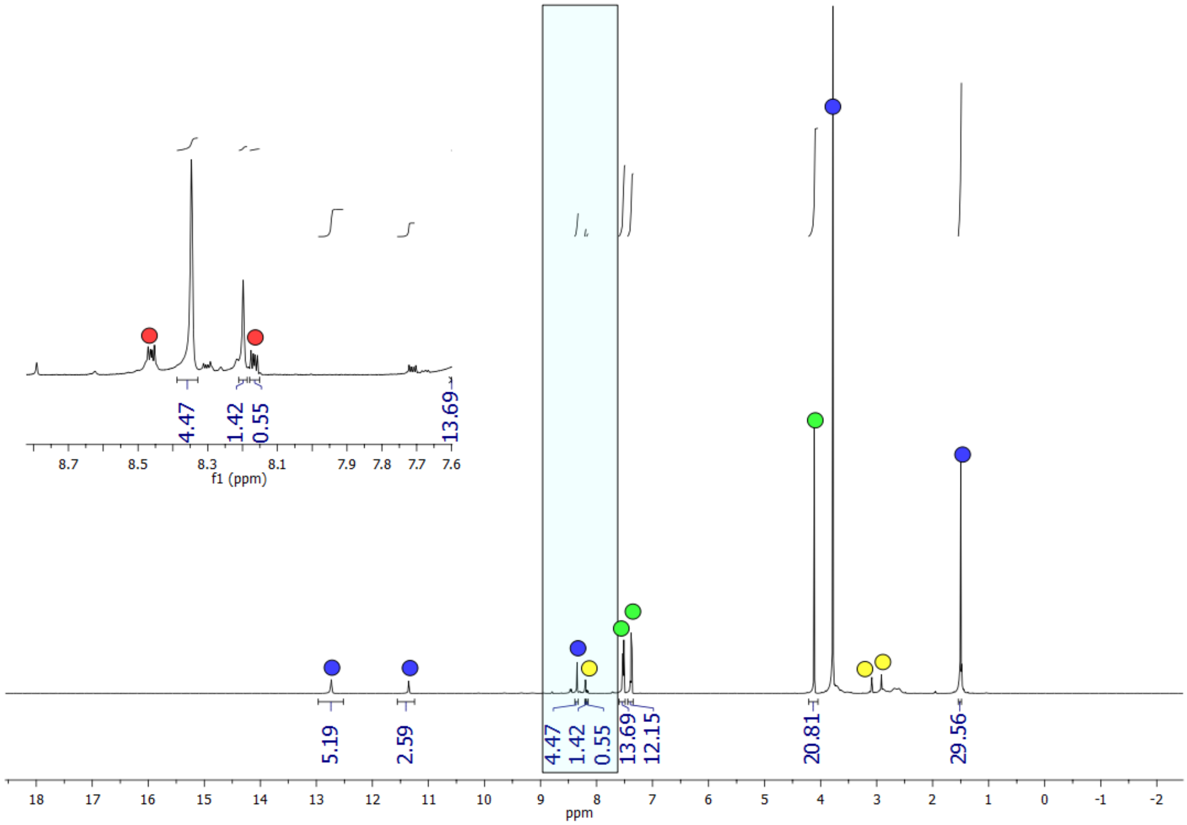

Figure S9: ^{1}H NMR (DMF-d_7, 500 MHz, 21 °C) spectrum of the mixture formed from the reaction of [K(18-crown-6)]$_2$[O_2⊂mBDCA-5t-H_6] with CO_2 in the presence of 5 eq DHA. The DMF peaks are indicated by yellow circles, anthraquinone by red circles, DHA by green circles, and the carbonate cryptate by blue circles.

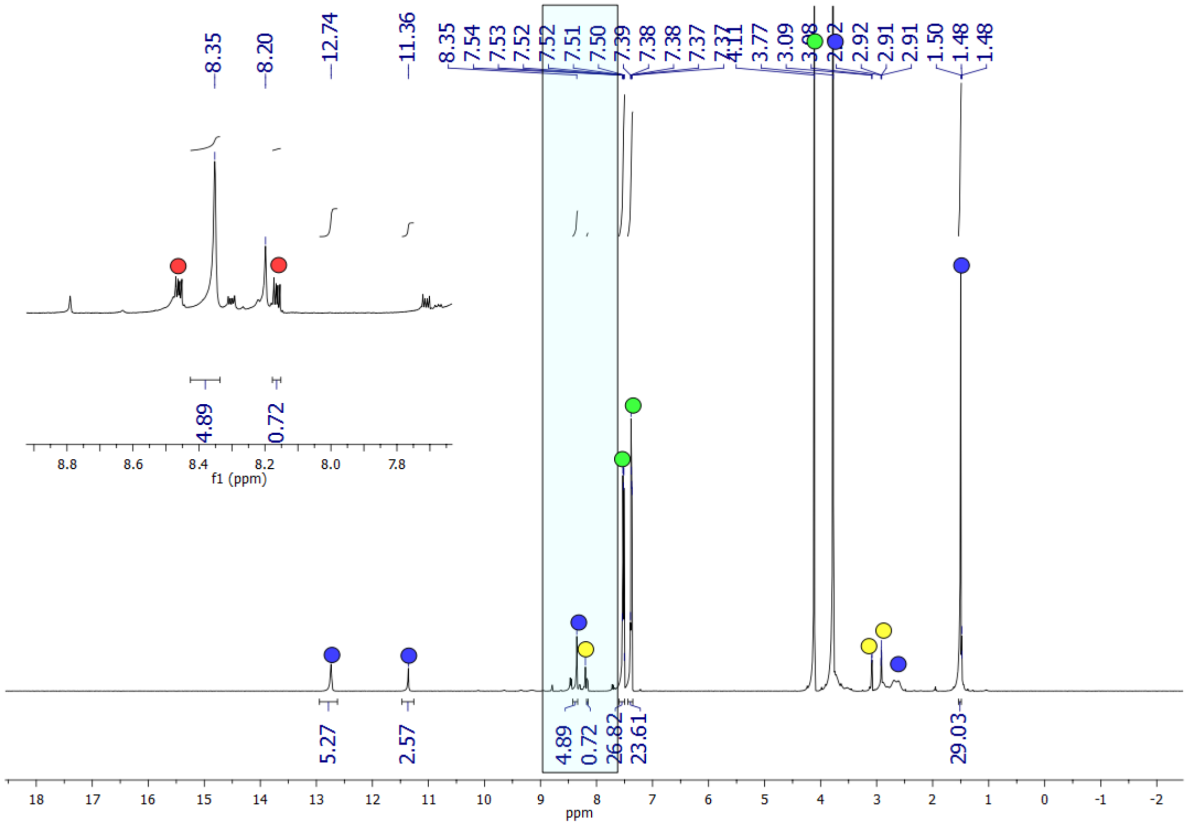

Figure S10: ^{1}H NMR (DMF-d_7, 500 MHz, 21 °C) spectrum of the mixture formed from the reaction of $[K(18\text{-crown-6})]_2[O_2{\subset}m\text{BDCA-5t-H}_6]$ with CO_2 in the presence of 10 eq DHA. The DMF peaks are indicated by yellow circles, anthraquinone by red circles, DHA by green circles and the carbonate cryptate by blue circles.

3 Reactivity of Li_2O_2 with CO_2 in organic solvents

3.1 Treatment of Li_2O_2 with CO_2 (1 atm) in DME

In a glovebox, DME (0.500 mL) was transferred to four different vials charged with 10.4 mg (vial 1), 9.5 mg (vial 2), 11.4 mg (vial 3), and 10.3 mg (vial 4) of Li_2O_2 respectively. Vial 1 was set aside and served as a control. Vials 2, 3, and 4 were capped with silicone/PTFE septum caps and removed from the glovebox. Once outside the glovebox, the headspace of vials 2, 3, and 4 was degassed and backfilled with CO_2 (1 atm). The suspensions in all four vials were stirred at 25 °C. After 48 h, the headspace of vial 1, 2, 3, and 4 was analyzed by Gas Chromatography (GC) equipped with

a thermal conductivity detector (multiple gas analyzer #3, SRI Instrument) for O_2 quantification. The amount of O_2 generated was calculated by comparing the O_2 peak area to that of a standard with 5200 ppm O_2 (Table S3). Afterward, vials 2, 3 and 4 were degassed and brought back into the glovebox. The suspensions in each vial were filtered to afford colorless homogeneous solutions. The formation of methyl methoxyacetate (MMA), one of the possible products of the DME solvent oxidation, was analyzed by ^{1}H NMR spectroscopy. The filtrate (0.100 mL) was transferred to an NMR tube with DMSO-d_6 (0.500 mL). A benzene solution in DMSO-d_6 (0.010 mL, 0.500 M) was added to each tube as an internal standard. To obtain reliable ^{1}H NMR integrations, the spectra were measured with a presaturation pulse sequence[1] to suppress the residual DME solvent peaks. The yield of methyl methoxyacetate (MMA) was calculated based on the integrations of the peak located at 4.04 ppm (Figure S11, Table S3 and Table S4). The residual solids collected by filtrations were washed with *ca*. 3 mL ether and dried under vacuum before subjection to Li_2O_2 and Li_2CO_3 quantification (see *Li_2O_2 and Li_2CO_3 quantification protocols* below).

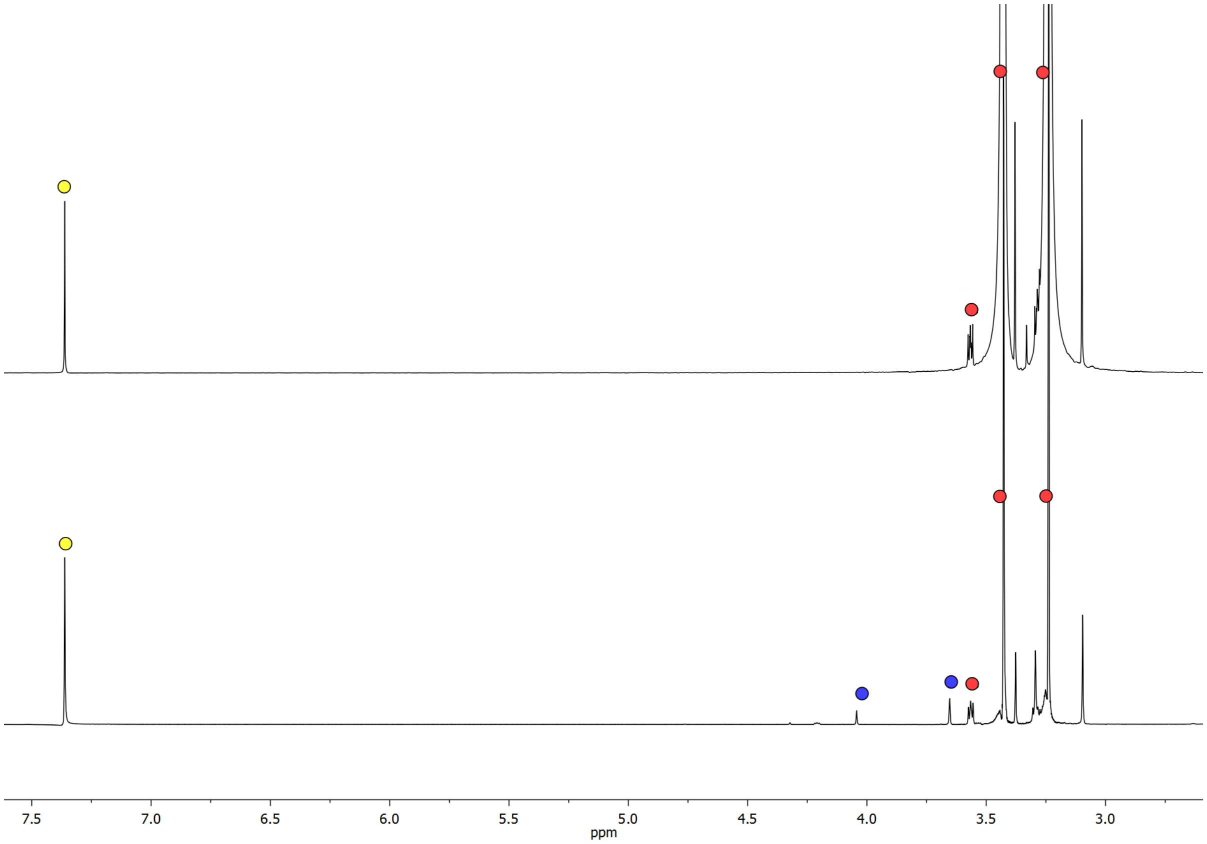

Figure S11: ^{1}H NMR (DMSO-d_6, 500 MHz, 21 °C) spectra of the mixture formed from the reaction of Li_2O_2 with DME under N_2 (1 atm, top) or CO_2 (1 atm, bottom). The methyl methoxyacetate (MMA) peaks are indicated by blue circles, benzene by yellow circles, and DME by red circles. To obtain reliable ^{1}H NMR integrations, the spectra were measured with a presaturation pulse sequence[1] to suppress the residual DME solvent peaks.

Table S3: Quantification of methyl methoxyacetate (MMA), O_2, and Li_2CO_3 from the mixture formed from the reactions of Li_2O_2 with CO_2 (1 atm) in DME.

Experiment	Li_2O_2 consumed	Li_2CO_3 produced	MMA produced	O_2 produced
2	0.129 mmol	0.140 mmol	0.0085 mmol	0.0917 mmol
3	0.115 mmol	0.159 mmol	0.0120 mmol	0.0829 mmol
4	0.115 mmol	0.133 mmol	0.0060 mmol	0.0797 mmol

Table S4: The percentage yields of methyl methoxyacetate (MMA), O_2, and Li_2CO_3 from the reactions of Li_2O_2 with CO_2 (1 atm) in DME.

Experiment	Li_2CO_3 yield*	MMA yield	O_2 yield
2	108%	13%	71%
3	137%	21%	72%
4	134%	12%	80%

* The LiOH, Li_2O and starting Li_2CO_3 impurities in commercial Li_2O_2 perhaps contributed to the formation of extra Li_2CO_3.

3.2 Treatment of Li_2O_2 with CO_2 (1 atm) in DMSO

In a glovebox, DMSO (1.000 mL) was transferred to four different vials charged with 19.8 mg (vial 1), 21.3 mg (vial 2), 20.1 mg (vial 3), and 20.4 mg (vial 4) of Li_2O_2 respectively. Vial 1 was set aside as a control. Vials 2, 3, and 4 were capped with silicone/PTFE septum caps and removed from the glovebox. Once outside the glovebox, the headspace of vials 2, 3, and 4 was replaced by CO_2 (*ca.* 1 atm) by bubbling CO_2 through the solution for *ca.* 30 s. The solutions in all four vials were stirred at 25 °C for 48 hours. After 48 h, vials 2, 3 and 4 were degassed and brought back into the glovebox. The suspensions in each vial were filtered to afford colorless homogeneous solutions. The formation of $DMSO_2$, one of the possible decomposition products of DMSO solvent oxidation, was analyzed by ^1H NMR spectroscopy. Each resulting solution (0.100 mL) was transferred to an NMR tube with DMSO-d_6 (0.500 mL). A acetonitrile solution in DMSO-d_6 (10 μL, 0.250 M) was added to each tube as an internal standard. The yield of $DMSO_2$ was calculated based on integrations of the $DMSO_2$ peaks located at 2.94 ppm (Figure S12, Table S5). The residual solids collected by filtrations were washed with ether (*ca.* 3 mL) and dried under vacuum before subjection to Li_2O_2 quantification (see *Li_2O_2 quantification protocol* below).

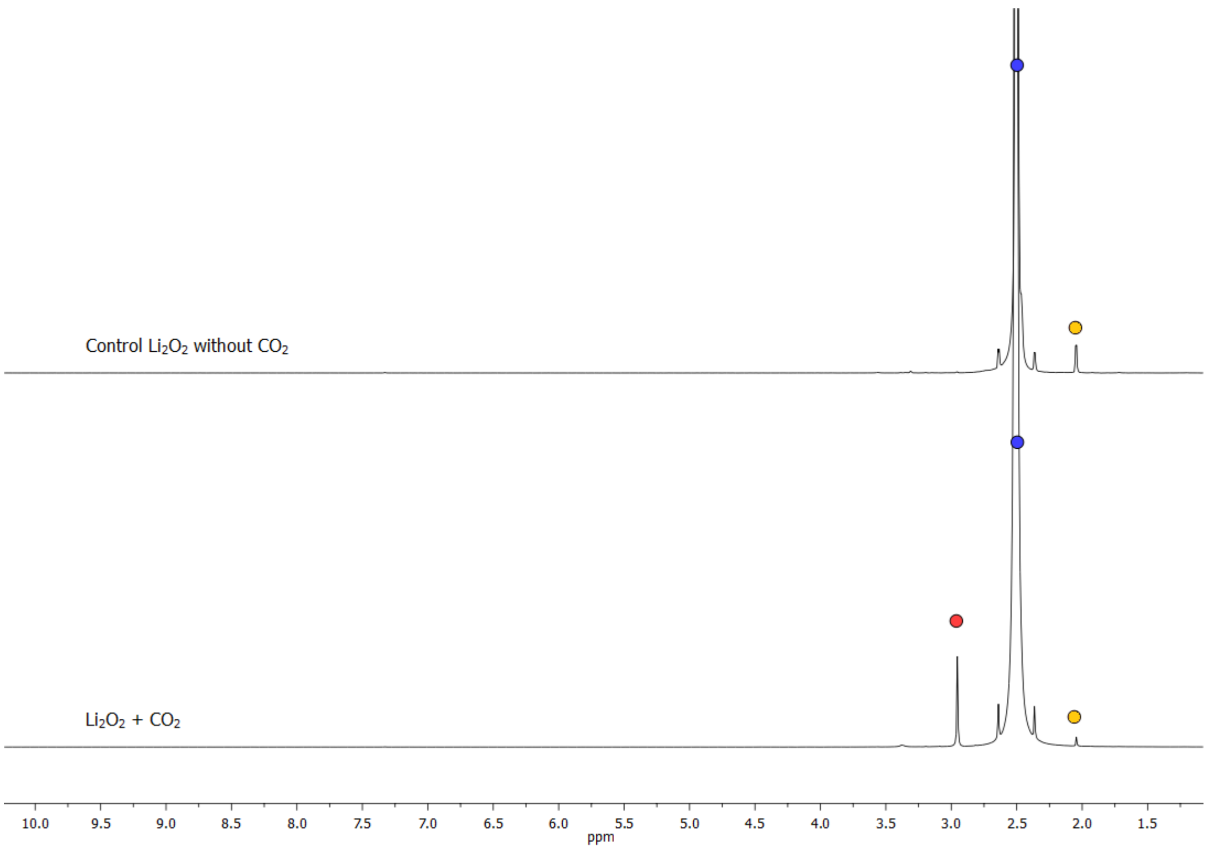

Figure S12: ^{1}H NMR (DMSO-d_6, 500 MHz, 21 °C) spectrum of the mixture formed from the reaction of Li_2O_2 with CO_2 in DMSO. The DMSO peaks are indicated by blue circles, $DMSO_2$ by a red circle and acetonitrile internal standard by yellow circles.

Table S5: Quantification of $DMSO_2$ from the mixture formed from the reactions of Li_2O_2 with CO_2 (1 atm) in DMSO.

Experiment	Li_2O_2 consumed	$DMSO_2$ produced	$DMSO_2$ yield
2	0.424 mmol	0.369 mmol	87%
3	0.400 mmol	0.362 mmol	90%
4	0.406 mmol	0.377 mmol	93%

3.3 Treatment of Li_2O_2 with 2,2,6,6-tetramethylpiperidone (4-oxo-TEMP) under CO_2 (1 atm)

In a glovebox, a solution of lithium bis(trifluoromethane)sulfonimide (LiTFSI, 0.500 M) and 2,2,6,6-tetramethylpiperidone (0.100 M) in diglyme (0.500 mL) was transferred to four different vials charged with 19.1 mg (vial 1), 20.7 mg (vial 2), 19.5 mg (vial 3), and 19.5 mg (vial

4) of Li_2O_2 respectively. Vial 1 was set aside as a control. Vials 2, 3, and 4 were capped with silicone/PTFE septum caps and removed from the glovebox. Diglyme and LiTFSI were used to simulate reaction conditions employed by Wandt *et al.* (see reference 9 in main text). Once outside the glovebox, the headspace of vials 2, 3, and 4 was replaced by CO_2 (*ca.* 1 atm) by bubbling CO_2 through the solution for *ca.* 30 s. The solutions in all four vials were stirred at 25 °C for 24 hours. After 24 h, vials 2, 3 and 4 were degassed and brought back into the glovebox. The suspensions in each vial were filtered and the resulting homogeneous solutions (50 μL) from each sample were transferred to capillary tubes inside 4 mm diameter quartz tubes for EPR analysis. The yield of 4-oxo TEMPO from the four reactions was calculated by comparing their doubly integrated EPR signals to that of a standard TEMPO solution (5.0 mM, diglyme). The residual solids collected by filtrations were washed with ether (*ca.* 3 mL) and dried under vacuum before subjection to Li_2O_2 quantification (see *Li_2O_2 quantification protocol* below).

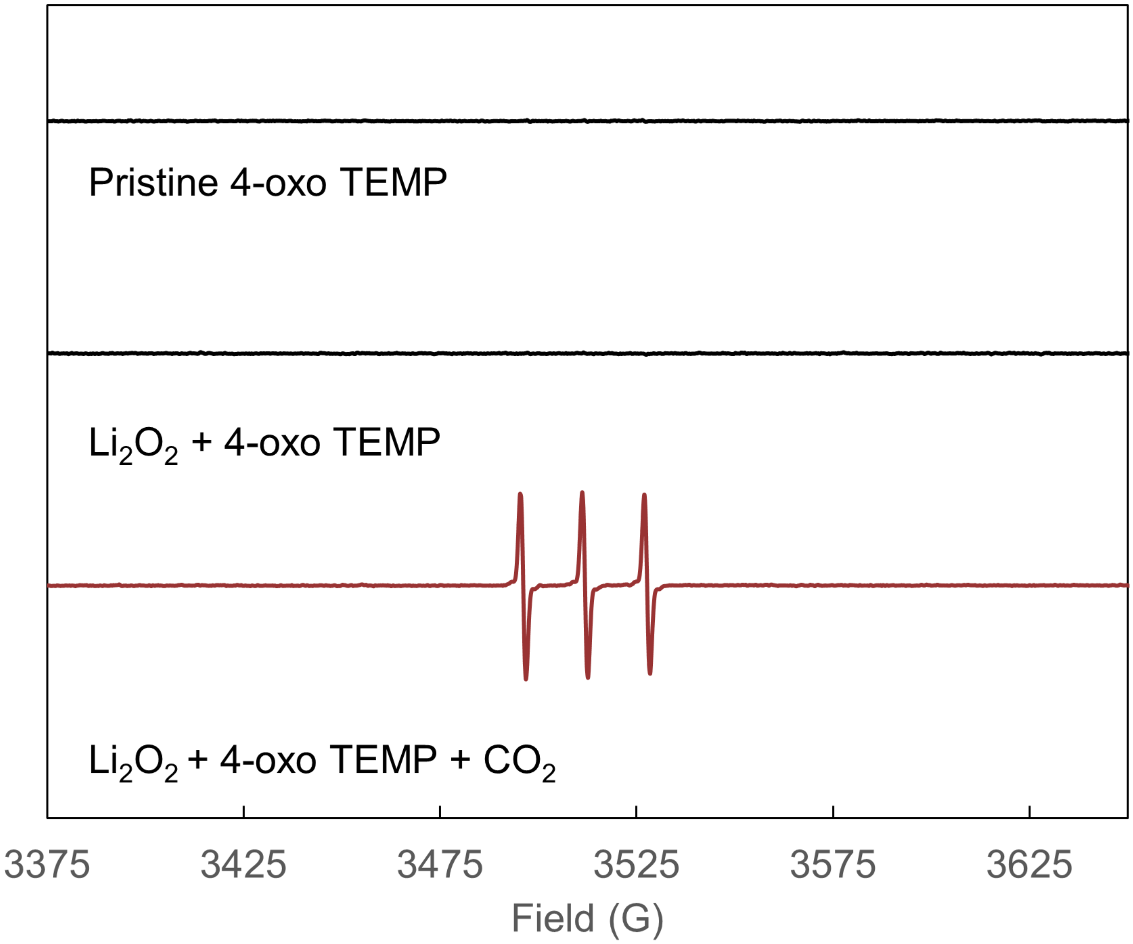

Figure S13: EPR spectra (25 °C) of (a) a pristine solution of 2,2,6,6-tetramethylpiperidone (0.100 M) and LiTFSI (0.500 M) in diglyme, (b) a solution of 2,2,6,6-tetramethylpiperidone (0.100 M) and LiTFSI (0.500 M) mixed with Li_2O_2 under N_2 atmosphere for 24 h, (c) a solution of 2,2,6,6-tetramethylpiperidone (0.100 M) and LiTFSI (0.500 M) mixed with Li_2O_2 under CO_2 (1 atm) for 24 h.

EPR measurements were performed in glass capillary tubes inside 4 mm quartz tubes. EPR spectra were recorded at 25 °C on a Bruker EMX spectrometer with an ER 4199HS cavity and a Gunn diode microwave source producing X-band (8-10 GHz) radiation. A modulation frequency of 100 kHz and a time constant of 20.48 ms were employed. The spin yield of 4-oxo TEMPO was calculated by comparing the intensity of doubly integrated EPR signals to that of a standard TEMPO solution (5.0 mM, diglyme) (Table S6 and Table S7).

Table S6: Doubly integrated intensity of 4-oxo TEMPO from the reactions between Li_2O_2 and 2,2,6,6-tetramethylpiperidone under CO_2 (1 atm).

Experiment	Doubly integrated intensity (A.U.)
2	2250287
3	2306612
4	2207382
TEMPO(5.00 mM)	1850660

Table S7: Quantification of 4-oxo TEMPO from the reactions between Li_2O_2 and 2,2,6,6-tetramethylpiperidone under CO_2 (1 atm).

Experiment	Li_2O_2 consumed	4-oxo-TEMPO produced	4-oxo-TEMPO yield
2	0.154 mmol	0.030 mmol	20%
3	0.218 mmol	0.031 mmol	14%
4	0.264 mmol	0.030 mmol	11%

3.4 Li_2O_2 quantification protocol

Typically, Li_2O_2 quantification were performed within 3 hours of terminating the reactions. The remaining solids collected from reactions mentioned above were dried under vacuum and weighted accurately in Agilent GC vials and transferred out of the glovebox. D_2O (1.000 mL) was added to the vials using a syringe. The vial content was sonicated to afford a homegonous solution. A known amount (normally 0.1-0.5 mL) of the resulting solution was transferred to an NMR tube followed by a solution of phenylboronic acid (0.500 mL, 0.110 M, DMSO-d_6) and D_2O (0.200 mL). The ^{1}H NMR spectrum of the samples was measured after 1 h. H_2O_2 is known to react with phenylboronic acid to form phenol quantitatively under basic conditions.[2] Therefore, the Li_2O_2 weight fraction in the sample may be calculated based on the ^{1}H NMR integration of phenol and phenylboronic acid using equation 1 and 2, where $w_{Li_2O_2}$ is the weight fraction of Li_2O_2, r is the molar ratio between Li_2O_2:$PhB(OH)_2$, and R is the molar ratio between PhOH:$PhB(OH)_2$ calculated based on ^{1}H NMR integration (Figure S14).

$$R = \frac{w_{Li_2O_2} \times r}{1 - r \times w_{Li_2O_2}} \tag{1}$$

$$w_{Li_2O_2} = \frac{R}{r + rR} \tag{2}$$

This protocol was found to be highly reproducible (Table S8). Control experiments were performed on a commercial Li_2O_2 sample from Sigma-Aldrich (90% purity). A series of solutions with different ratios of Li_2O_2:$PhB(OH)_2$ were prepared as shown in Table S8. ^{1}H NMR spectra of each solution were taken to determine the ratios of PhOH:$PhB(OH)_2$, which were used to calculate the percentage purity of Li_2O_2 using the equations 1 and 2, yielding an average Li_2O_2 purity of 91.4 (±1.9)% (Table S8).

Table S8: Determining the purity of commercial Li_2O_2.

Experiment	Li_2O_2:$PhB(OH)_2$(r)	PhOH:$PhB(OH)_2$(R)	Calculated purity % (w)
1	0.250	0.308	94.2
2	0.333	0.437	91.2
3	0.417	0.601	90.2
4	0.500	0.819	90.0

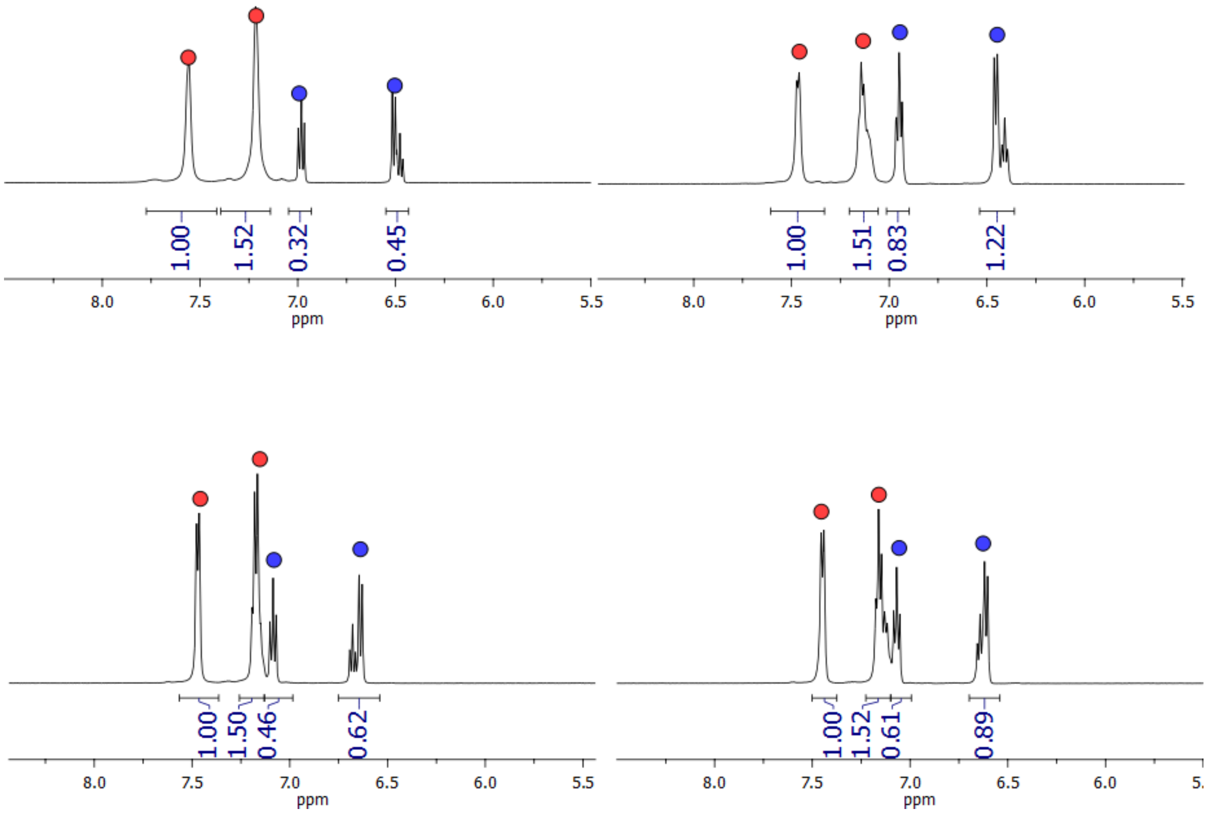

Figure S14: Li_2O_2 quantification using ^{1}H NMR (DMSO-d_6 and D_2O, 500 MHz, 21 °C) spectroscopy. The four experiments were set up with different ratios of Li_2O_2:PhB(OH)$_2$ top left: 0.250; top right: 0.500; bottom left: 0.333; bottom right: 0.417. The phenol peaks are indicated by blue circles and the phenylboronic acid by red circles.

The protocol outlined above was used to determine the conversion of Li_2O_2 to Li_2CO_3 in each reaction. The amount of Li_2O_2 consumed by CO_2 ($n_{Li_2O_2}$) was calculated by subtracting the amount of Li_2O_2 after the reaction from that before the reaction using equations 3 and 4, where $w_{Li_2O_2}$ and $w'_{Li_2O_2}$ are the weight fractions of Li_2O_2 before and after the reaction, $M_{Li_2CO_3}$ and $M_{Li_2O_2}$ are the molecular weights of Li_2CO_3 and Li_2O_2, $n_{Li_2O_2}$ is the amount of Li_2O_2 consumed during the reaction and $m_{Li_2O_2}$ is the weight of the Li_2O_2 starting materials.

$$w'_{Li_2O_2} = \frac{m_{Li_2O_2} \times w_{Li_2O_2} - n_{Li_2O_2} \times M_{Li_2O_2}}{m_{Li_2O_2} + n_{Li_2O_2} \times (M_{Li_2CO_3} - M_{Li_2O_2})} \quad (3)$$

$$n_{Li_2O_2} = \frac{m_{Li_2O_2} \times (w_{Li_2O_2} - w'_{Li_2O_2})}{w'_{Li_2O_2} \times (M_{Li_2CO_3} - M_{Li_2O_2}) + M_{Li_2O_2}} \quad (4)$$

Li_2O_2 quantification was performed in triplicate. Li_2O_2 was not detected from the filtrate isolated in section 3.2. Therefore, Li_2O_2 was assumed to be fully consumed in the case of the conditions described in section 3.2.

3.5 Li_2CO_3 quantification protocol

Typically, Li_2CO_3 quantification was performed within 3 hours of terminating the reactions. The remaining solids collected from the Li_2O_2 and CO_2 reaction were dried under vacuum and weighted accurately in Agilent GC vials. D_2O (1.00 mL) was added to the vials using a syringe. The vial contents were sonicated to afford homogeneous solutions. The solutions were further diluted by ten-fold by transferring 0.500 mL of the solution to Milli-Q water (4.500 mL). The amount of Li_2CO_3 in each sample was analyzed by Total Organic Carbon analyzing kits (HACH) with a detecting range of 30-300 ppm. A modified testing procedure was used: sample (1.00 mL) was added to the clear reaction vial. The vial was quickly capped and immediately connected to the blue indicator vial. The vial assembly was heated at 100 °C for 2.5 hours, then removed from the heating block to cool down to 25 °C. The blue indicator vial was inserted into a Varian Cary 50 UV-vis spectrometer, and a UV-vis spectrum was taken. The absorbance at 438 nm is proportional to the concentration of Li_2CO_3 present in the solution. A calibration curve was constructed using standard solutions with known amounts of Li_2CO_3 (Figure S15).

The protocol outlined above was used to determine the yield of Li_2CO_3 from the reaction of Li_2O_2 with CO_2 (1 atm) in DME. In some cases, the yield of Li_2CO_3 exceeded 100%, perhaps due to the fact that commercial Li_2O_2 is often contaminated with Li_2O and LiOH, which can be converted to Li_2CO_3 under a CO_2 atmosphere.[3]

A

Sample	C content (ppm)	A_{438nm}
A	45.4	0.127
B	91.0	0.242
C	136.5	0.372
D	181.9	0.522
E	272.9	0.816

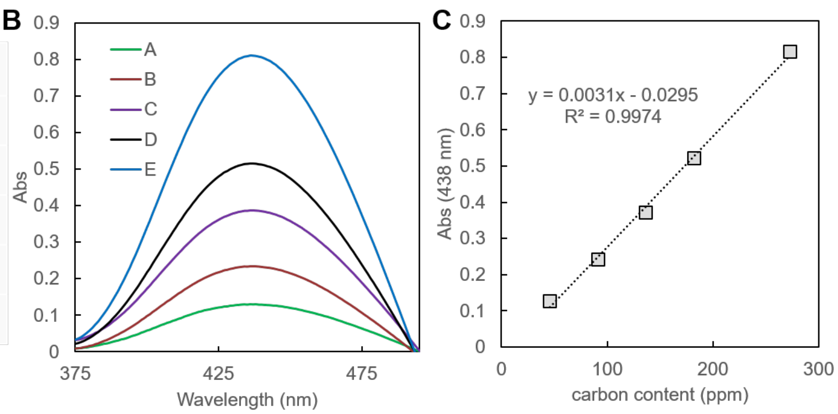

Figure S15: Li_2CO_3 quantification using HACH TOC analyzing kit. (a) solutions used to construct the calibration curve, (b) UV-vis profiles of Li_2CO_3 standards, (c) plot of carbon concentration (ppm) *vs*. absorbance at 438 nm.

4 Variable temperature NMR spectroscopy studies

4.1 ^{13}C and ^{1}H NMR analysis of the mixture resulting from the reaction of [K(18-crown-6)]$_2$[O_2⊂*m*BDCA-5t-H_6] with $^{13}CO_2$

[K(18-crown-6)]$_2$[O_2⊂*m*BDCA-5t-H_6] (49.3 mg, 0.0331 mmol) was dissolved in DMF-d_7 (*ca*. 0.6 mL) and the solution was transferred to a J Young NMR tube. The solution was frozen in a −78 °C dry ice/acetone bath. The headspace was evacuated for 1 min and backfilled with excess $^{13}CO_2$ (3.00 mL, 1 atm, 0.123 mmol, 3.7 equiv). The sample was allowed to thaw in the NMR spectrometer after the probe was cooled down to −50 °C. ^{13}C and ^{1}H NMR spectra were taken at −50, −40, −30, −20, and 21 °C. ^{13}C{^{1}H} NMR (DMF-d_7, 126 MHz, ppm) δ, Figure S16: 172.2 (s), 157.4 (s), 157.0 (s). The ^{13}C NMR spectra show two intermediate peaks (156.9 and 157.4 ppm) at low temperature. The species having a signal located at 156.9 ppm converted to [K(18-crown-6)]$_2$[CO_3⊂*m*BDCA-5t-H_6] (172.2 ppm) above −30 °C. The intermediate with a resonance at 157.4 ppm converted to [K(18-crown-6)]$_2$[CO_3⊂*m*BDCA-5t-H_6] (172.2 ppm) at 21 °C over the course of 1 h. The ^{1}H NMR spectra (Figure S17) show the formation of [K(18-crown-6)]$_2$[CO_3⊂*m*BDCA-5t-H_6] and appearance of three broad resonances at 9.61, 9.23 and 8.39 ppm, assigned to the

monodeprotonated anion receptor [K(18-crown-6)][*m*BDCA-5t-H_5] (see section 5, reference 12 in main text). Warming the sample up to 25 °C resulted in the decrease in the intensity of peaks associated with [K(18-crown-6)][*m*BDCA-5t-H_5] concomitant with formation of [K(18-crown-6)]$_2$[CO_3⊂*m*BDCA-5t-H_6].

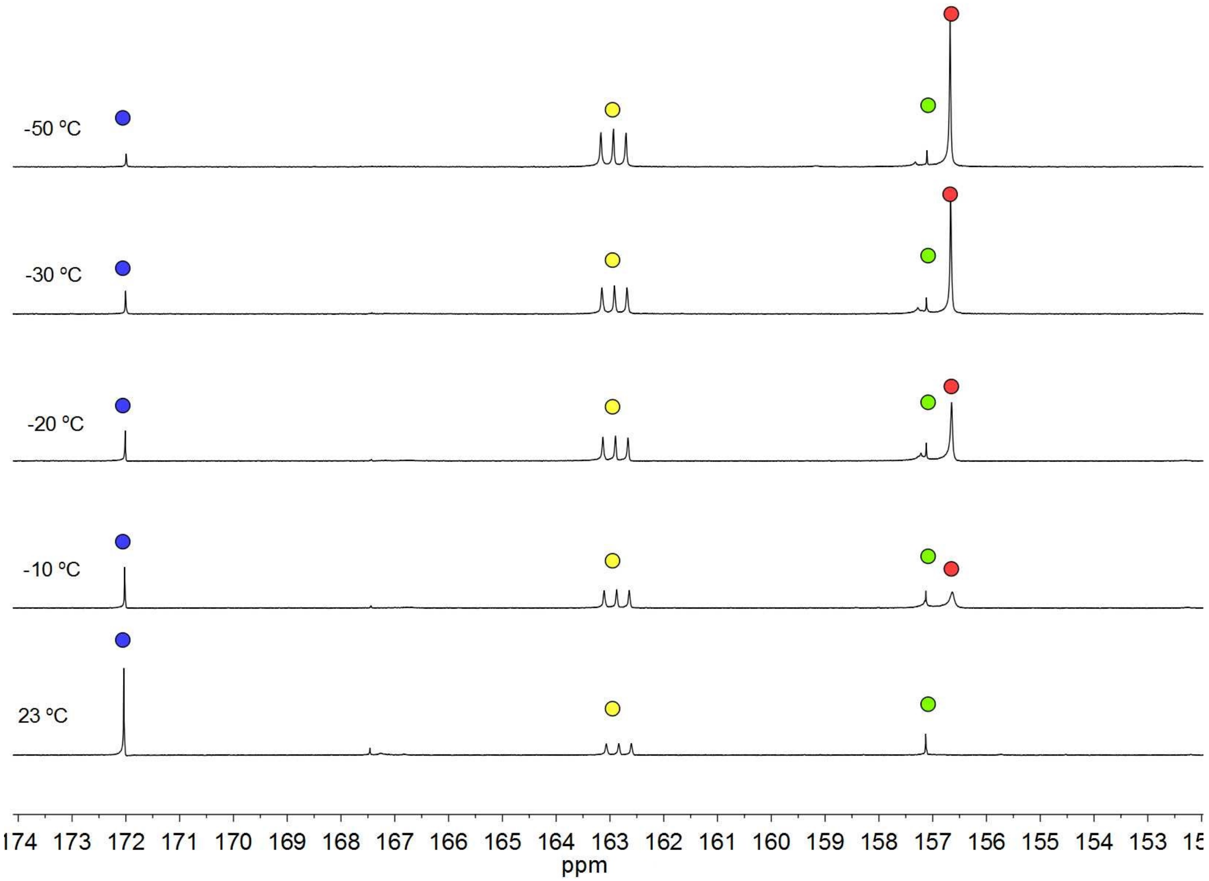

Figure S16: ^{13}C{^{1}H} NMR (DMF-d_7, 126 MHz) spectra of the mixture formed from the reaction of [K(18-crown-6)]$_2$[O_2⊂*m*BDCA-5t-H_6] with $^{13}CO_2$ at −50, −40, −30, −20, 0, and 21 °C. The DMF peaks are indicated by yellow circles, the carbonate cryptate by blue circles, hydroperoxy-carbonate $HOOCO_2^-$ by green circles and peroxydicarbonate $^-O_2COOCO_2^-$ by red circles.

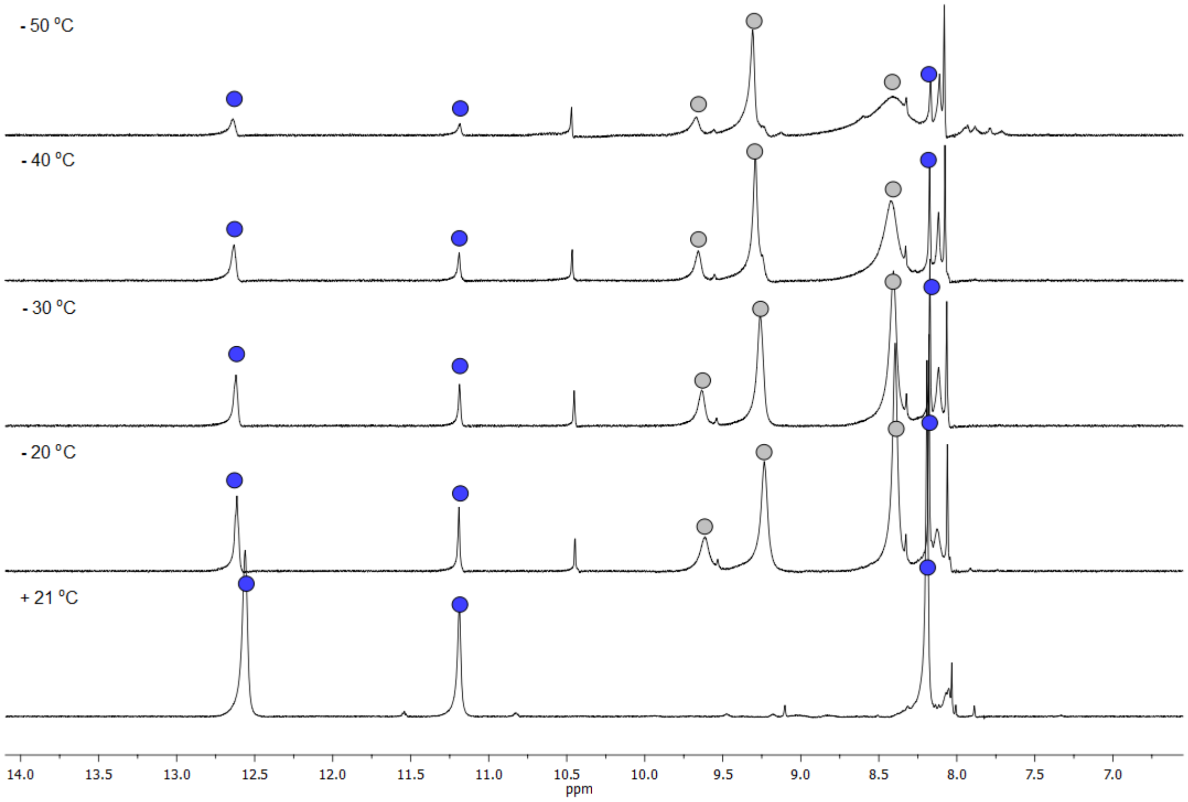

Figure S17: ^{1}H NMR (DMF-d_7, 500 MHz) spectra of the mixture formed from the reaction of [K(18-crown-6)]$_2$[O_2⊂*m*BDCA-5t-H_6] with CO_2 in DMF-d_7 at −50, −40, −30, −20, and 21 °C. The carbonate cryptate by blue circles, monodeprotonated anion receptor [K(18-crown-6)][*m*BDCA-5t-H_5] by grey circles.

4.2 ^{17}O NMR analysis of the mixture resulting from the reaction of [K(18-crown-6)]$_2$[$^{17}O_2$⊂*m*BDCA-5t-H_6] with $^{13}CO_2$

[K(18-crown-6)]$_2$[$^{17}O_2$⊂*m*BDCA-5t-H_6] (24.3 mg, 0.0163 mmol) was dissolved in DMF (0.500 mL) and the solution was transferred to a J Young NMR tube. The solution was frozen in a −78 °C dry ice/acetone bath. The headspace was evacuated for 1 min and backfilled with excess $^{13}CO_2$ (3.00 mL, 1 atm, 0.123 mmol, 7.5 equiv). The sample was allowed to thaw in the NMR spectrometer after the probe was cooled down to −50 °C. ^{17}O NMR spectra of the reaction mixture (Figure S18) were taken at −50, −40, −30, −20, 0, and 21 °C. An intermediate peak located at 274.8 ppm grew in at −50 °C. Upon gradually warming the sample up to −10 °C, the intensity of

the signal was seen to decay and ultimately resolved into two peaks with equal intensities, observed at 278.7 and 264.0 ppm.

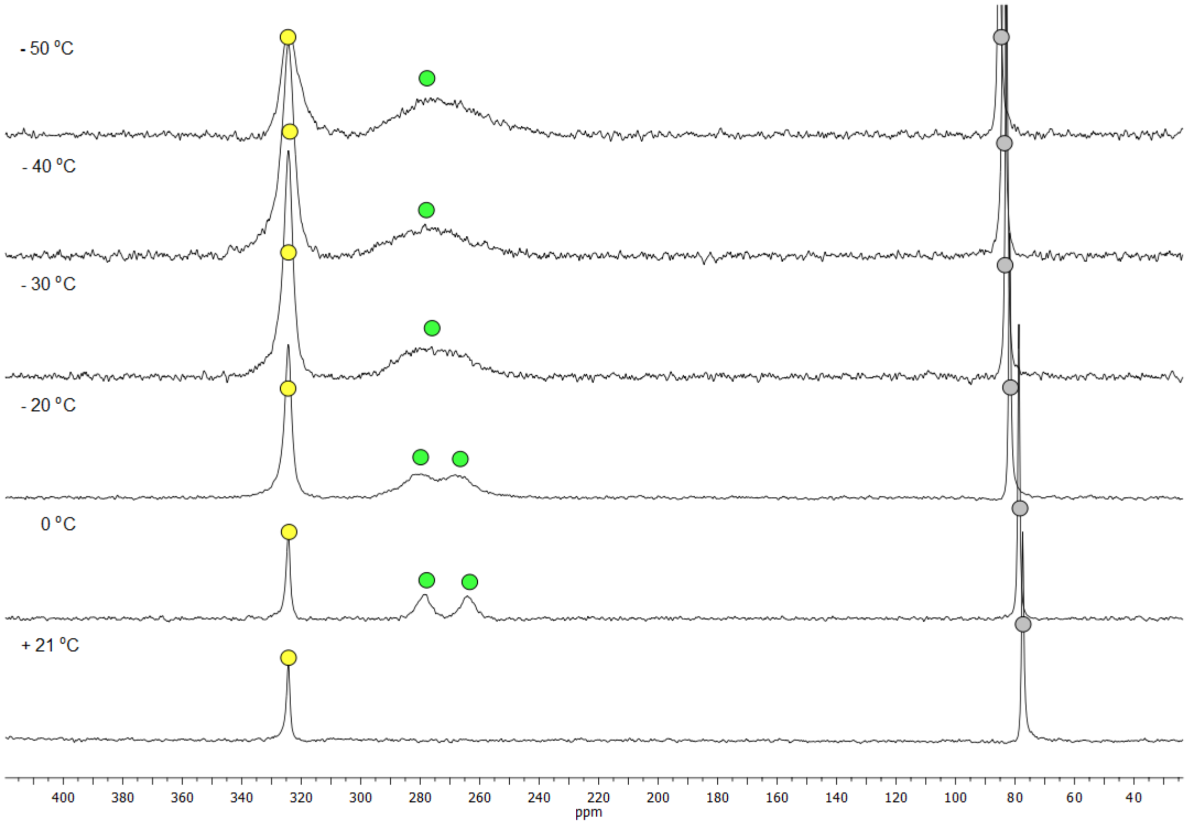

Figure S18: ^{17}O NMR (DMF, 68 MHz) spectra of the mixture formed from the reaction of **1**-$^{17}O_2$ with CO_2 as a function of temperature at −50, −40, −30, −20, and 21 °C. The DMF peak is indicated by yellow circles, CO_2 by grey circles, and hydroperoxycarbonate $HOOCO_2^-$ by green circles. Peroxide cryptate, and carbonate cryptate are ^{17}O NMR silent (see reference 20 in main text).

4.3 Monitoring the reaction of [K(18-crown-6)]$_2$[O$_2$⊂*m*BDCA-5t-H$_6$] and $^{13}CO_2$ in the presence of PPh_3

[K(18-crown-6)]$_2$[O$_2$⊂*m*BDCA-5t-H$_6$] (24.3 mg, 0.0147 mmol, 1 equiv) was dissolved in DMF-d_7 (*ca.* 0.5 mL) and the solution was transferred to a J Young NMR tube. The solution was frozen in a −78 °C dry ice/acetone bath. The headspace was evacuated for 1 min and backfilled with excess $^{13}CO_2$ (3.00 mL, 1 atm, 0.134 mmol, 9.1 equiv). The sample was allowed to thaw in the

NMR spectrometer after the probe was cooled down to −50 °C. A ^{13}C NMR spectrum was taken to confirm the formation of $^{-}O_2COOCO_2^{-}$ and $HOOCO_2^{-}$. After 30 minutes, the NMR tube was removed from the spectrometer and the solution was frozen in a −78 °C dry ice/acetone bath. To the NMR tube was added PPh_3 (24.3 mg, 0.0147 mmol, 3 equiv) under an N_2 atmosphere. The sample was sealed and transferred back to the NMR spectrometer cooled at −50 °C. The progress of the reaction was monitored by ^{13}C NMR spectroscopy at −30 °C for 90 minutes (Figure S19). The peaks at 156.9 ppm and 157.4 ppm, which correspond to $^{-}O_2COOCO_2^{-}$ and $HOOCO_2^{-}$, were seen to decay along with formation of CO_2 (126.0 ppm) and carbonate cryptate (172.2 ppm). ^{1}H NMR analysis indicates the formation of $OPPh_3$ and monodeprotonated anion receptor (Figure S20).

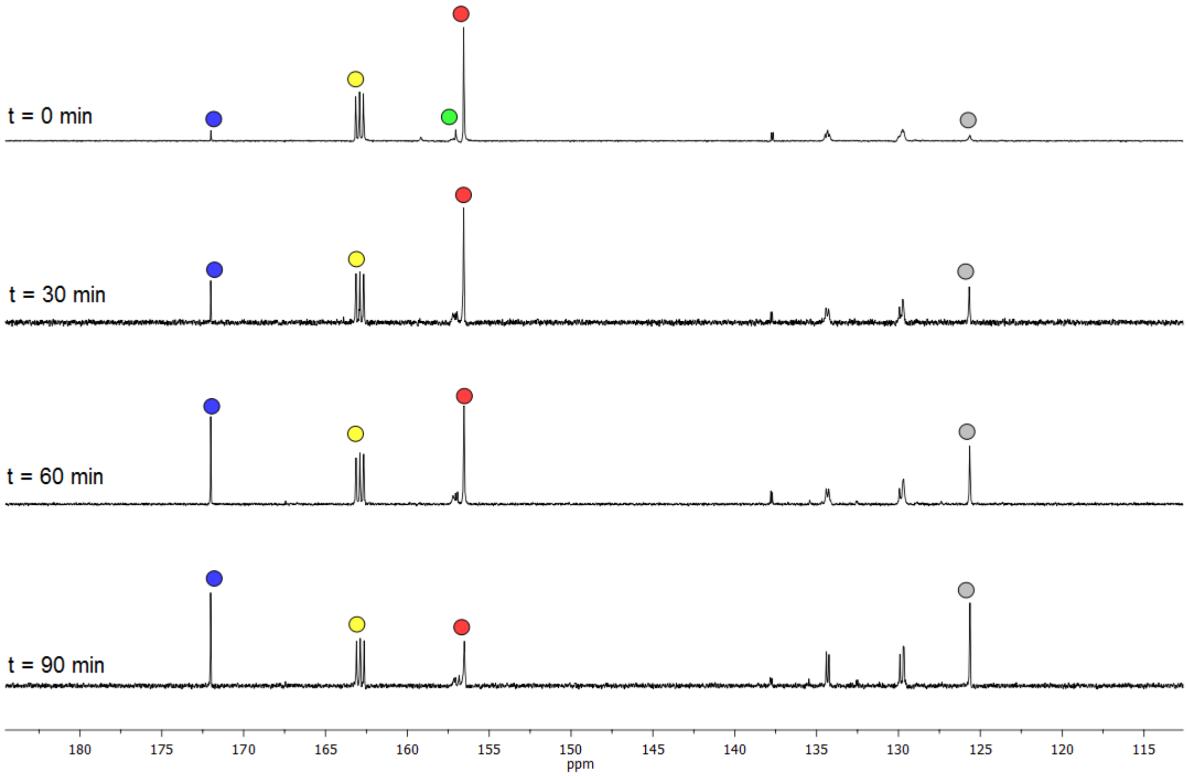

Figure S19: $^{13}C\{^{1}H\}$ NMR (DMF-d_7, 126 MHz) spectra of the mixture formed from the reaction of $[K(18\text{-crown-6})]_2[O_2{\subset}m\text{BDCA-5t-}H_6]$ with $^{13}CO_2$ in the presence of PPh_3 at −30 °C (30 min per scan). The DMF peaks are indicated by yellow circles, carbonate cryptate by blue circles, $^{-}O_2COOCO_2^{-}$ by red circles, $HOOCO_2^{-}$ by a green circle and CO_2 by grey circles.

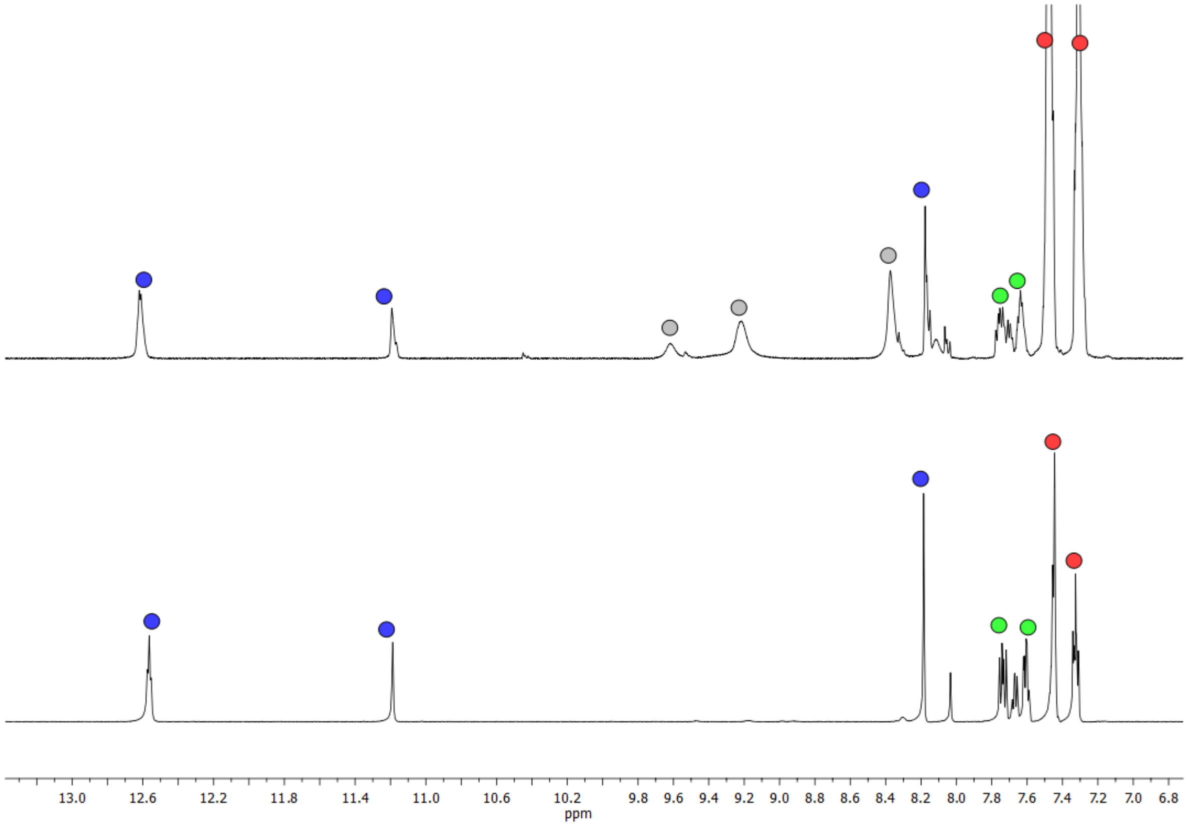

Figure S20: ^{1}H NMR (DMF-d_7, 500 MHz) spectra of the mixture formed from the reaction of $[K(18\text{-crown-6})]_2[O_2{\subset}m\text{BDCA-5t-}H_6]$ with $^{13}CO_2$ in the presence of PPh_3 in DMF-d_7 as a function of temperature (top: −30 °C; bottom: 21 °C). The carbonate cryptate peaks are indicated by blue circles, monodeprotonated anion acceptor by grey circles, $OPPh_3$ by green circles, and PPh_3 by red circles.

4.4 Generation of hydroperoxycarbonate $HOO^{13}CO_2^-$ from $[PPN][H^{13}CO_3]$ and H_2O_2

$$[PPN][H^{13}CO_3] + H_2O_2 \xrightarrow[\text{DMF, } -20\ ^\circ\text{C}]{} [PPN][O_2{}^{13}COOH] + H_2O \qquad (5)$$

$[PPN][H^{13}CO_3]$ was synthesized from PPNCl and $NaH^{13}CO_3$ based on a modified literature procedure.[4] To a boiling solution of PPNCl (140.0 mg, 0.244 mmol) in H_2O (2.8 mL), $NaH^{13}CO_3$ (558.0 mg, 6.56 mmol, 27 equiv) in H_2O (10 mL) was added slowly to yield a clear solution. The resulting solution was placed in a ice bath. The white precipitate that formed after 2 h was collected by filtration, washed with cold water (2 × 5 mL), cold ether (3 × 5 mL), and dried un-

der reduced pressure to afford 89.7 mg product (0.149 mmol, 61% yield). The ^{1}H NMR and ^{13}C NMR spectrum of [PPN][$H^{13}CO_3$] so obtained are in agreement with data from the literature.[4] In a glovebox, [PPN][$H^{13}CO_3$] (40.0 mg, 0.0743 mmol) was dissolved in DMF (0.5 mL) and the solution was transferred to an NMR tube equipped with a septum. The NMR tube was sealed and taken out of the glovebox. The solution was frozen by placing the NMR tube in a −78 °C cold bath. Hydrogen peroxide (126 μL, 2.23 mmol, 30 equiv, 50% w/w H_2O) was added and the mixture was allowed to thaw at −20 °C to afford a homogeneous solution. A ^{13}C NMR spectrum of the sample was taken at −20 °C (Figure S21). A new peak located at 157.5 ppm was assigned to hydroperoxycarbonate $HOOCO_2^-$.

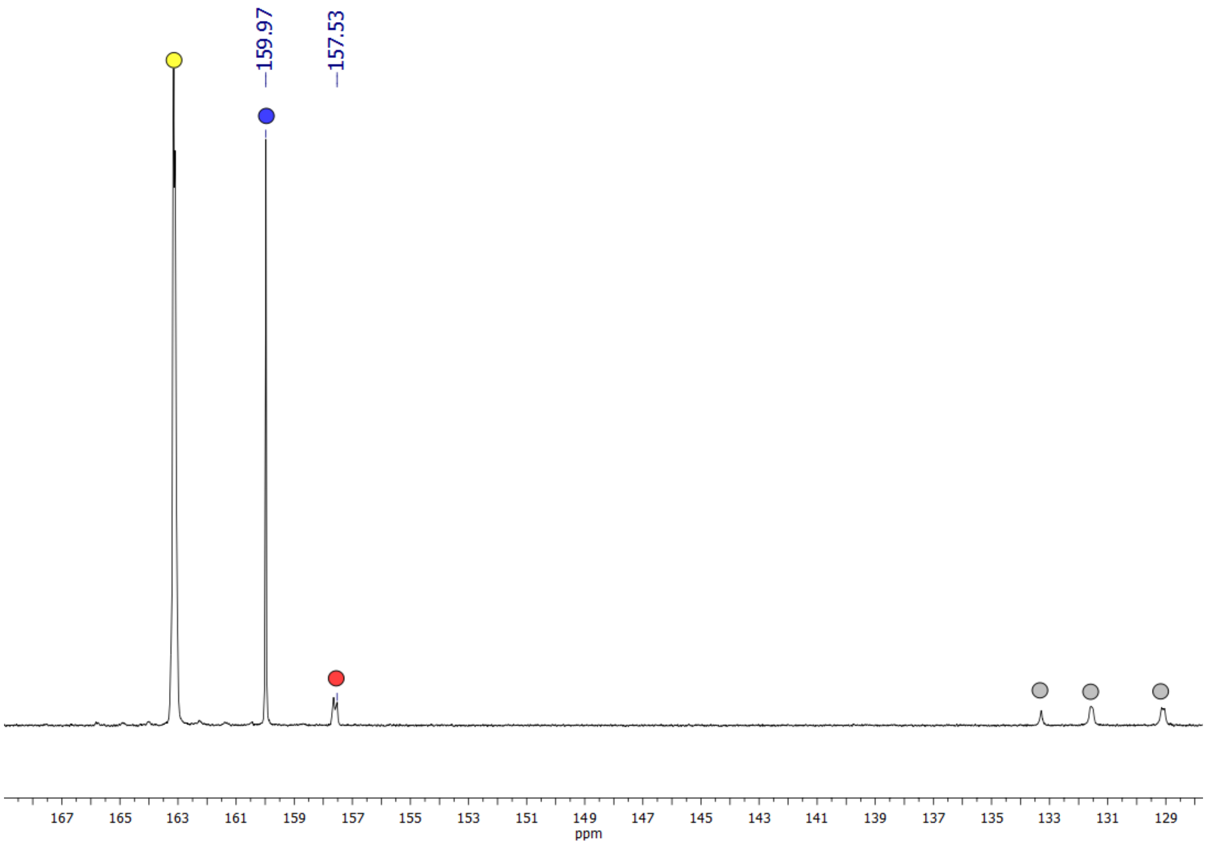

Figure S21: ^{13}C{^{1}H} NMR (DMF-d_7, 126 MHz, −20 °C) spectrum of hydroperoxycarbonate $HOOCO_2^-$ *in situ* generated from [PPN][HCO_3] and H_2O_2. The DMF peak is indicated by a yellow circle, bicarbonate/carbonic acid by a blue circle, hydroperoxycarbonate $HOOCO_2^-$ by a red circle, and PPN anion by grey circles.

4.5 Generation of peroxydicarbonate $^{-}O_2{}^{13}COO^{13}CO_2{}^{-}$ from bis(trimethylsilyl) peroxide (TMSOOTMS), potassium *tert*-butoxide (KO*t*Bu) and $^{13}CO_2$ in DMF

$$0.5\ \text{TMSOOTMS} + \text{KO}t\text{Bu} + \text{CO}_2 \xrightarrow[\text{DMF, -40 °C}]{} 0.5\ \text{K}_2[\text{O}_2\text{COOCO}_2] + \text{TMSO}t\text{Bu} \quad (6)$$

In a glovebox, a J Young NMR tube was charged with a DMF solution of KO*t*Bu (12.5 mg, 0.111 mmol, 0.100 mL). The NMR tube was sealed and the solution was frozen at 77 K. DMF (0.100 mL) was added to the NMR tube, followed by a DMF solution of TMSOOTMS (9.9 mg, 0.056 mmol, 0.300 mL, 0.5 equiv) at −78 °C under an N_2 atmosphere. The J Young NMR tube was capped, the headspace was evacuated for 1 min and back filled with $^{13}CO_2$ (*ca*. 1 atm). The sample was allowed to thaw in the NMR spectrometer with the probe pre-cooled at −40 °C. ^{13}C NMR spectra were taken at −40, −20, 0, and 21 °C (Figure S22). Upon gradually warming the sample up to 21 °C, the intensity of the signal for $^{-}O_2COOCO_2{}^{-}$ (155.7 ppm) was seen to decay along with formation of KO_2COtBu (157.5 ppm).

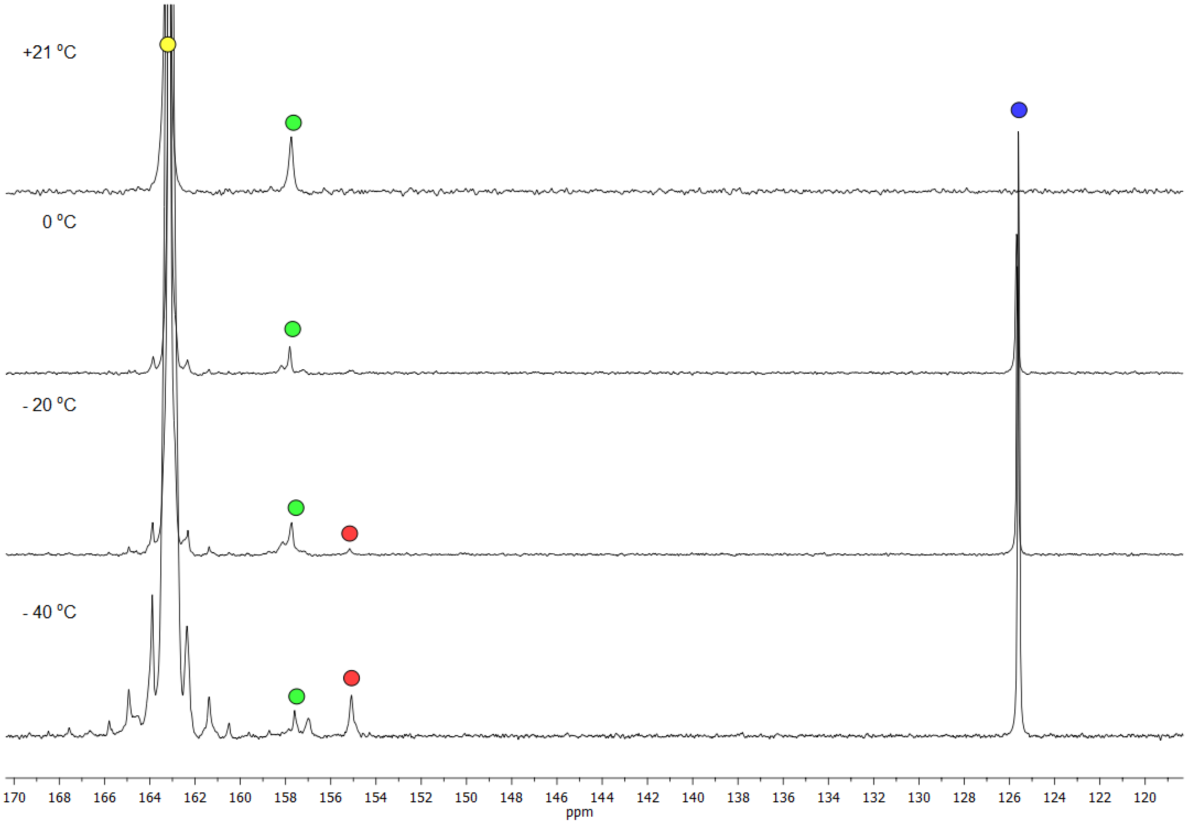

Figure S22: $^{13}C\{^{1}H\}$ NMR (DMF-d_7, 126 MHz) spectra of peroxydicarbonate $^{-}O_2COOCO_2^{-}$ *in situ* generated from TMSOOTMS, KO*t*Bu and $^{13}CO_2$ in DMF as a function of temperature (−40, −20, 0, and 21 °C). The DMF peak is indicated by a yellow circle, peroxydicarbonate $^{-}O_2COOCO_2^{-}$ by red circles, KO_2COtBu (157.5 ppm) by green circles, and CO_2 by a blue circle.

4.5.1 Control experiment A

In a glovebox, a J Young NMR tube was charged with TMSOOTMS (8.7 mg, 0.049 mmol) and 0.500 mL DMF. The J Young NMR tube was capped, the headspace was evacuated for 1 min and back filled with $^{13}CO_2$ (*ca.* 1 atm). The sample was allowed to thaw and a ^{13}C NMR spectrum was taken at 21 °C (Figure S23). No reaction was observed between TMSOOTMS and $^{13}CO_2$.

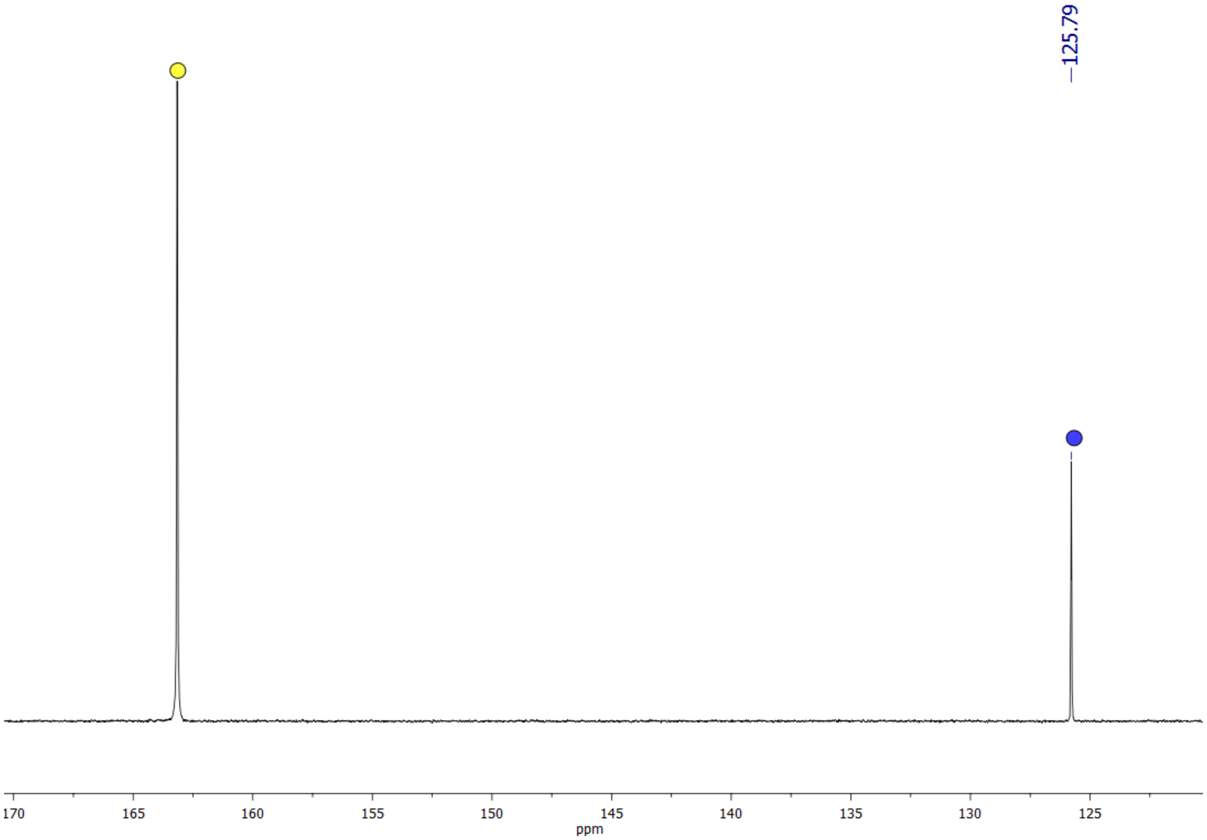

Figure S23: $^{13}C\{^1H\}$ NMR (DMF-d_7, 126 MHz, 21 °C) spectrum of the mixture obtained after treatment of TMSOOTMS with $^{13}CO_2$ in DMF at 21 °C. The DMF peak is indicated by a yellow circle and CO_2 by a blue circle.

4.5.2 Control experiment B

In a glovebox, a J Young NMR tube was charged with KO*t*Bu (12.4 mg, 0.111 mmol) and 0.500 mL DMF. The J Young NMR tube was capped, the headspace was evacuated for 1 min and back filled with $^{13}CO_2$ (*ca.* 1 atm). The sample was allowed to thaw and a ^{13}C NMR spectrum was taken at 21 °C (Figure S24). The ^{13}C NMR data indicate formation of KO_2COtBu at 157.5 ppm as the only ^{13}C containing product (Figure S24).

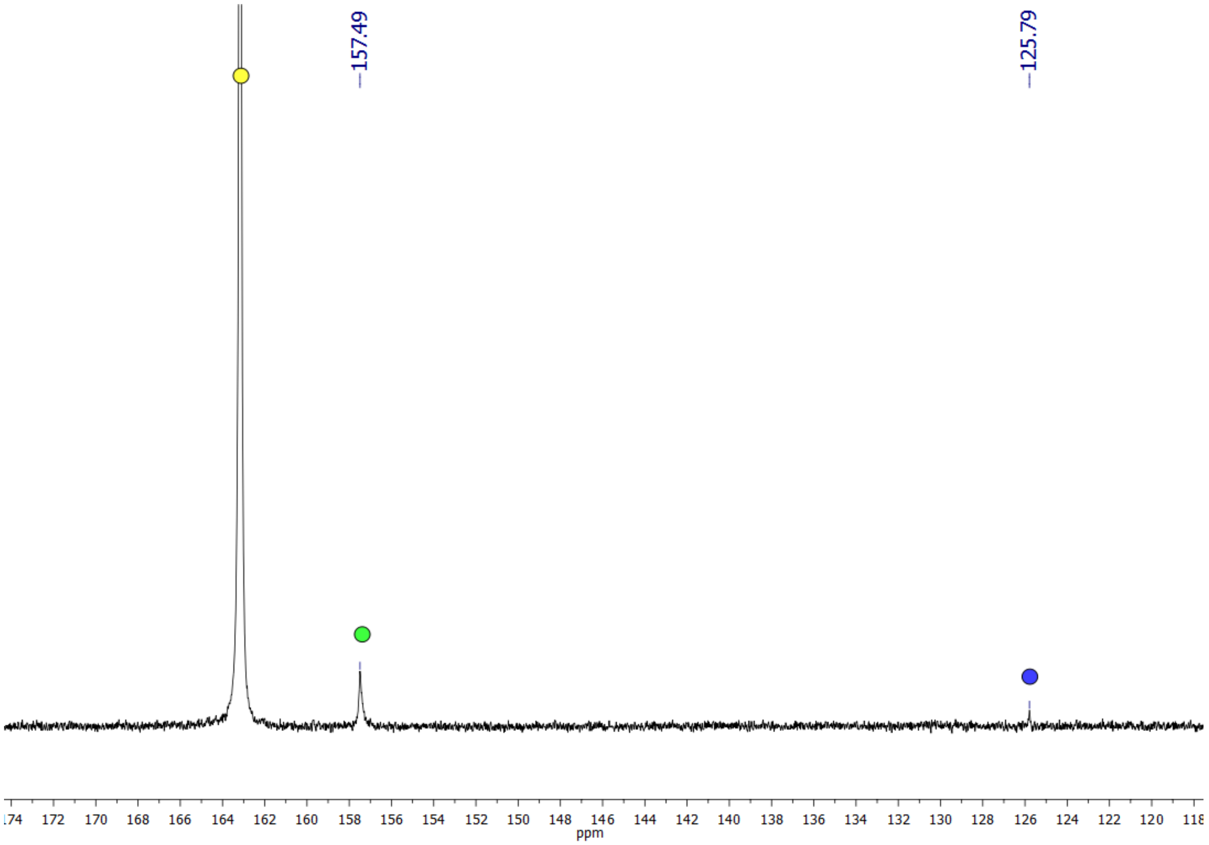

Figure S24: $^{13}C\{^{1}H\}$ NMR (DMF-d_7, 126 MHz, 21 °C) spectrum of the mixture obtained after treatment of KO*t*Bu with $^{13}CO_2$ in DMF at 21 °C. The DMF peak is indicated by a yellow circle, KO_2CO*t*Bu by green circles, and CO_2 by a blue circle.

4.6 Attempt to generate unsymmetrical peroxydicarbonate *in situ* from KO_2 and $^{13}CO_2$

In a glovebox, KO_2 (30 mg, 0.423 mmol) was added to DMF-d_7 (1 mL) and the resulting suspension was stirred at room temperature for 8 hours. The insoluble KO_2 was removed by filtration and the resulting homogeneous solution was transferred to a J-Young NMR tube. The solution was frozen in dry ice/acetone bath (−78 °C). The head space was evacuated for 1 min and back filled with $^{13}CO_2$ (*ca.* 1 atm). The NMR tube was allowed to warm up −20 °C in pre-cooled NMR probe for 10 mins. ^{13}C NMR spectra were taken at different temperature (−20, −10, 0, 22 °C). No new ^{13}C resonance was observed (Figure S25), perhaps due to the low solubility of KO_2 in organic media.

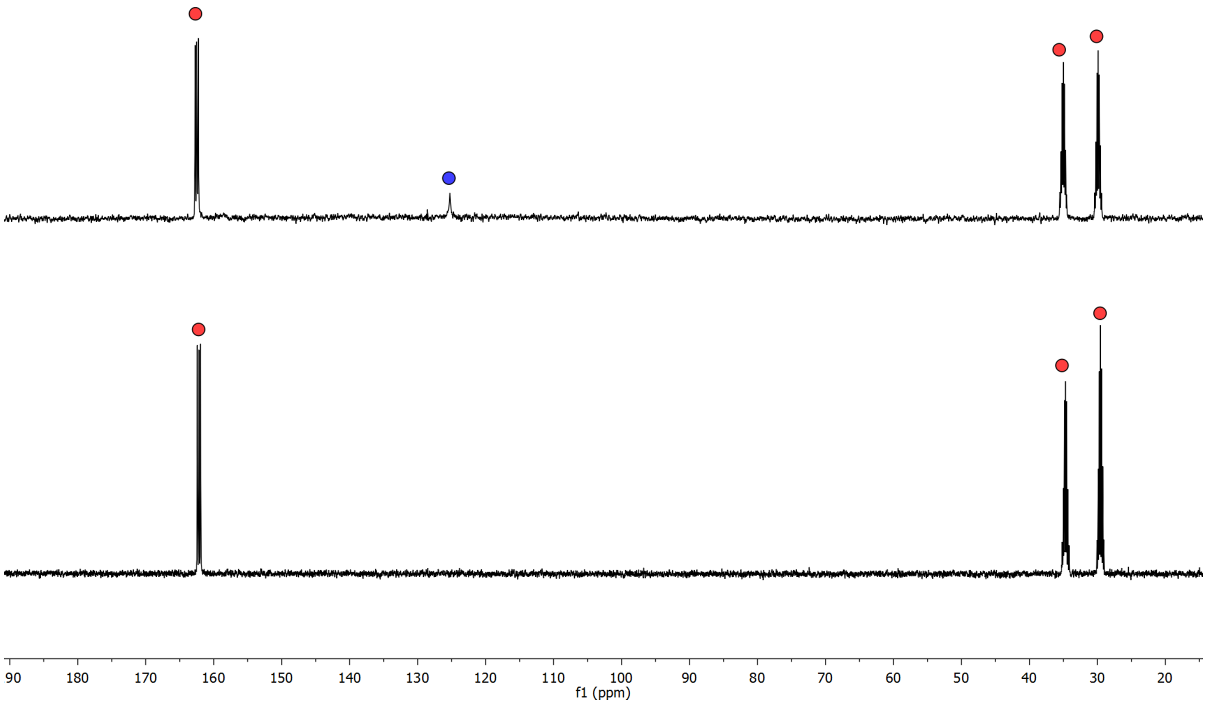

Figure S25: $^{13}C\{^1H\}$ NMR (DMF-d_7, 126 MHz, 21 °C) spectrum of the reaction mixture before (bottom) and after (top) the addition of $^{13}CO_2$. DMF peaks are indicated by red circles and CO_2 by a blue circle.

5 Synthesis and characterization of monodeprotonated anion receptor [mBDCA-5t-H$_5$]$^-$

5.1 Preparation of [K(18-crown-6)][mBDCA-5t-H$_5$]

Anion receptor *m*BDCA-5t-H$_6$ (128 mg, 0.124 mmol, 1 equiv), potassium *tert*-butoxide (15.5 mg, 0.136 mmol, 1.1 equiv), 18-crown-6 (36.3 mg, 0.136 mmol, 1.1 equiv), and THF (4 mL) were added to a scintillation vial (20 mL) equipped with a teflon stirbar. The resulting slurry was stirred at room temperature for 10 hours. The precipitate was collected on a fine frit, washed with THF (5 × 3 mL) and dried under dynamic vacuum for 3 hours. Yield: 131 mg (0.106 mmol, 86%) ^{1}H

NMR (400 MHz, DMSO-d_6, ppm, 21 °C): Figure S26, 9.38 (br, 3H), 8.22 (br, 6 H), 3.60 (m, 4 H), 3.54 (s, 27 H), 2.52 (b, 24 H), 1.76 (m, 4 H), 1.30 (s, 27 H). $^{13}C\{^1H\}$ NMR (126 MHz, DMSO-d_6, ppm, 21 °C): 165.70, 149.60, 126.98, 123.44, 69.80, 69.51, 66.98, 59.51, 53.20, 34.52, 31.10. Anal. Calcd. for [K(18-crown-6)][*m*BDCA-5t-H_5]···0.5THF ($C_{66}H_{101}KN_8O_{13.5}$): C, 62.72; H, 7.89; N, 9.14. Found: C, 61.81; H, 7.80; N, 9.03.

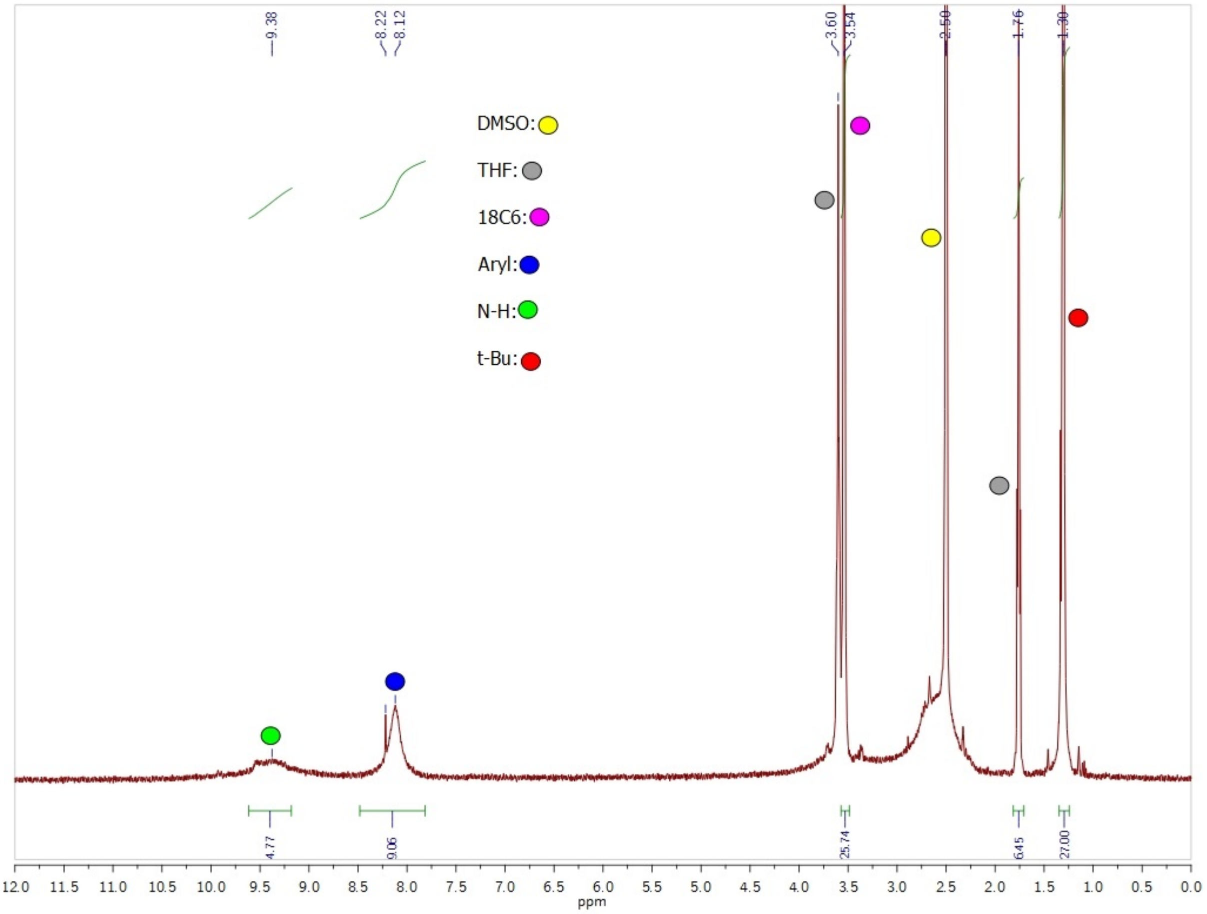

Figure S26: ^1H NMR (400 MHz, DMSO-d_6, 21 °C) spectrum of [K(18-crown-6)][*m*BDCA-5t-H_5].

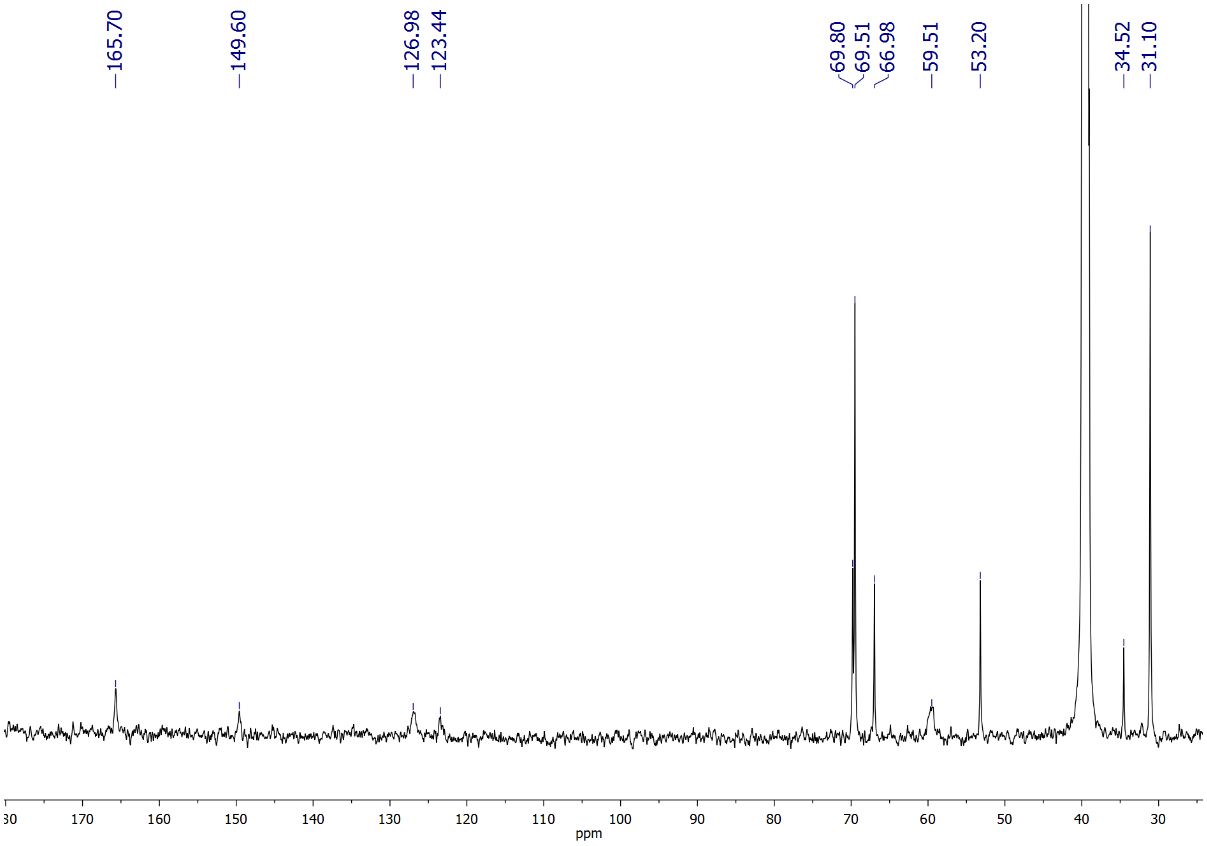

Figure S27: $^{13}C\{^{1}H\}$ NMR (126 MHz, DMSO-d_6, 21 °C) spectrum of [K(18-crown-6)][*m*BDCA-5t-H_5].

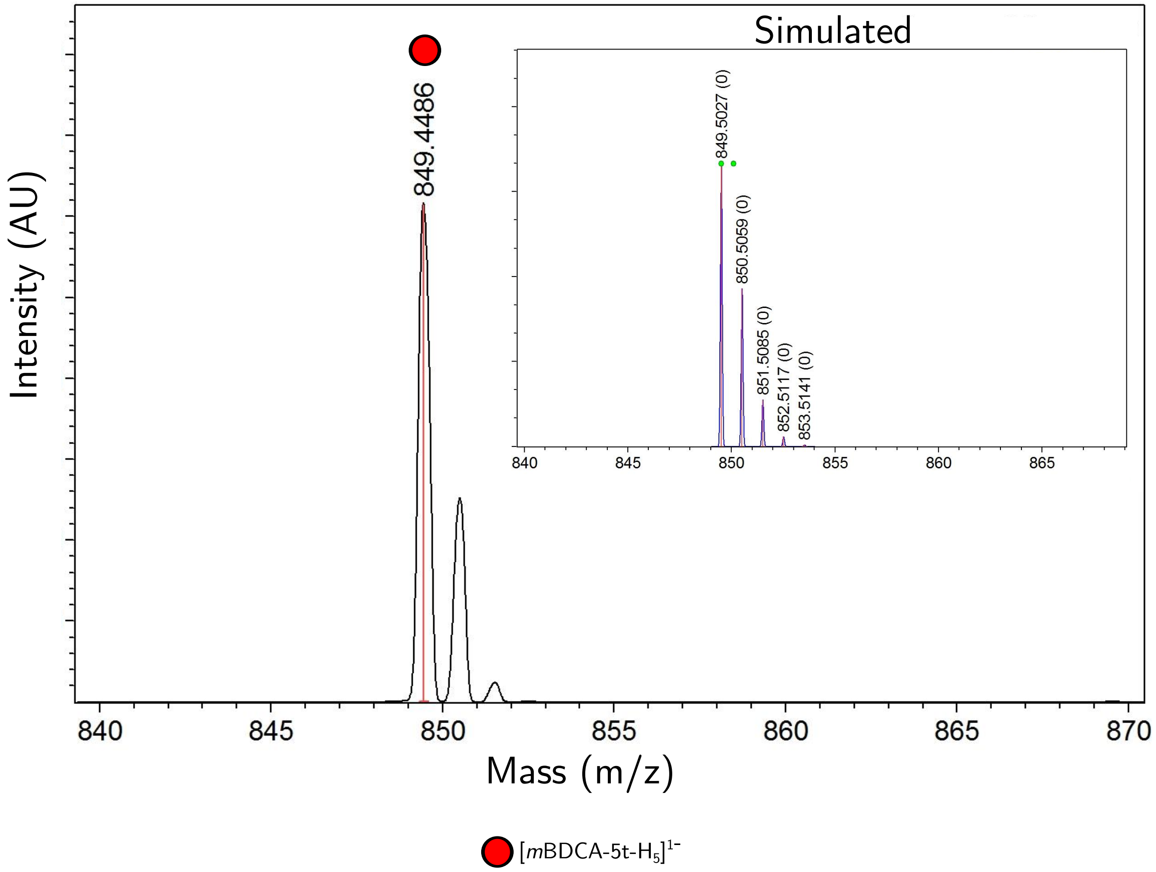

Figure S28: ESI-MS (negative mode) and simulation (inset) of [K(18-crown-6)][*m*BDCA-5t-H_5].

5.2 Preparation of [K(Kryptofix 222)][*m*BDCA-5t-H_5]

We decided to sequester the potassium ion of [K(18-crown-6)][*m*BDCA-5t-H_5] with Kryptofix 222 to investigate if monodeprotonated cryptand could be obtained as K(Kryptofix 222) salt. [K(18-crown-6)][*m*BDCA-5t-H_5] (148.4 mg, 0.1261 mmol, 1 equiv), Kyptofix 222 (47.5 mg, 0.1261 mmol, 1 equiv) and THF (5 mL) were transferred to a scintillation vial (20 mL) equipped with a teflon stirbar. The resulting slurry was stirred at room temperature for 16 hours. The resulting precipitate was collected on a fine frit, washed with THF (5 $\times$ 3 mL) and dried under dynamic vacuum for 3 hours. Yield: 107 mg (0.052 mmol, 67%). Crystals of [K(Kryptofix 222)][*m*BDCA-5t-H_5] were obtained after 4 days by vapor diffusion of diethyl ether into a MeCN solution of [K(Kryptofix 222)][*m*BDCA-5t-H_5]. ^{1}H NMR (500 MHz, DMSO-d_6, ppm, 21 °C): Figure S29,

9.32 (b, 3 H), 8.12 (br, 6 H), 3.53-3.36 (m, 36 H), 2.63 (m, 24 H), 1.30 (s, 27 H). Satisfactory elemental analysis results were not obtained for this salt due to its variable solvent content. MS-ESI (m/z), calculated 849.50, found 849.51. The solid state structure of [K(Kryptofix 222)][*m*BDCA-5t-H_5], as determined in a single-crystal X-ray diffraction study (see below) indicates that the full molecule is part of the asymmetric unit and the deprotonated carboxamide can be located; it has a significantly shorter C310–N302 distance (1.307(2) Å) and a longer C310–O302 bond (1.281(4) Å) in comparison with protonated moieties (C–N_{av}: 1.34 Å; C–O_{av}: 1.24 Å, Figure S30).

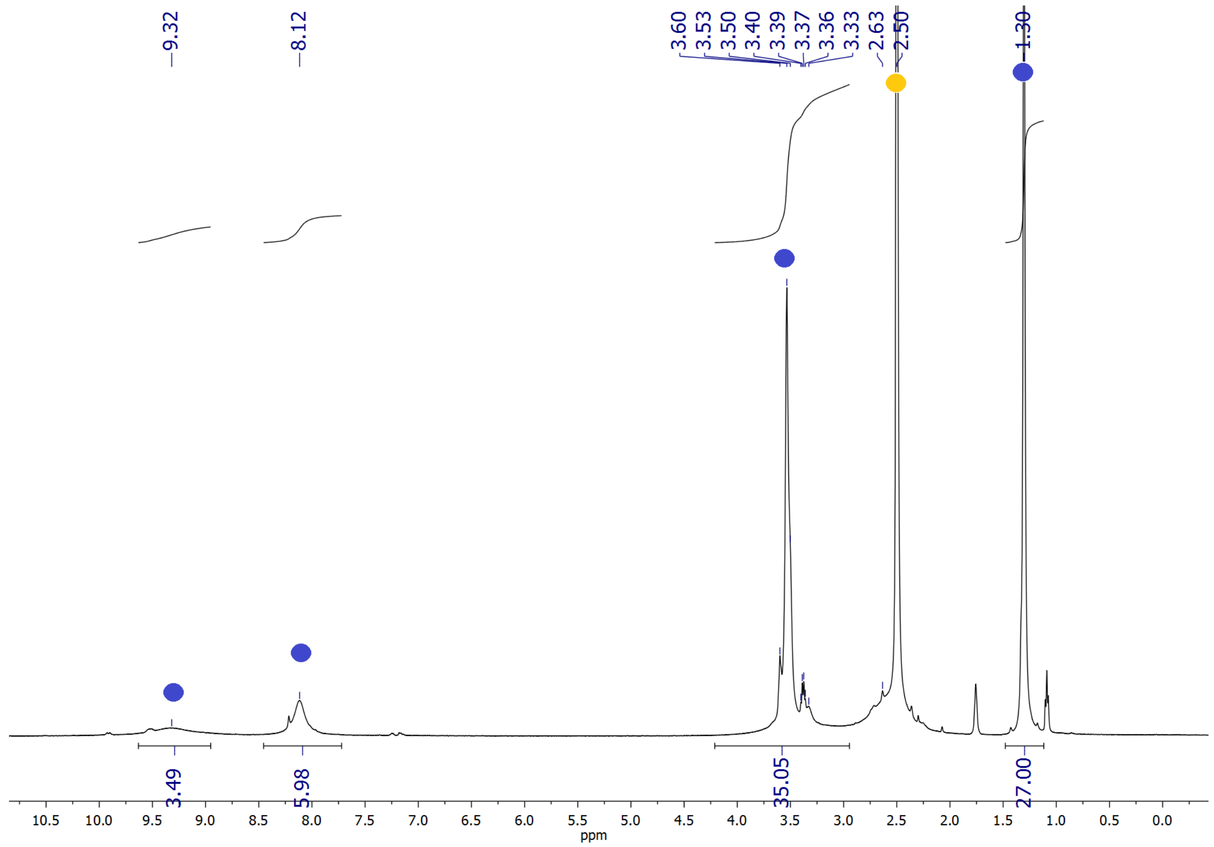

Figure S29: ^{1}H NMR (400 MHz, DMSO-d_6, 21 °C) spectrum of [K(Kryptofix 222)][*m*BDCA-5t-H_5]. The DMSO peak is indicated by a yellow circle and [K(Kryptofix 222)][*m*BDCA-5t-H_5] by blue circles.

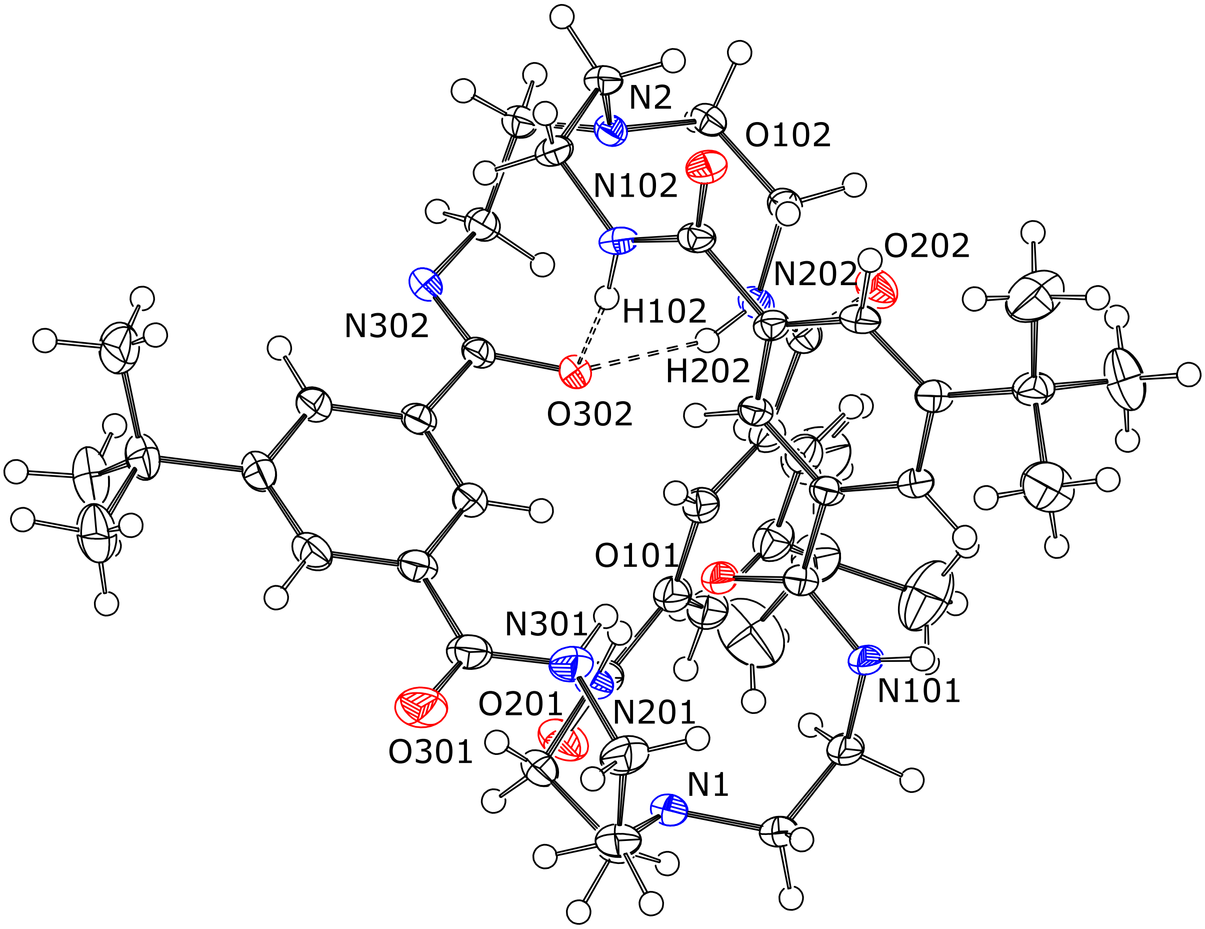

Figure S30: Solid-state structure of [K(Kryptofix 222)][*m*BDCA-5t-H_5] with thermal ellipsoids (drawn using PLATON[5]) shown at the 50% probability level. K(Kryptofix 222) cation was omitted for clarity. Selected interatomic distances (Å): O302–C301 1.281(4), O302–N202 2.774(4), O302–N102 2.959(4), O302–H202 1.9375, O302–H102 2.1708, O101–C103 1.239(4), O101–N201 2.887(4), O101–N301 3.131(5), O101–H201 2.0741, O101–H301 2.3603.

Low-temperature (100 K) diffraction data (ϕ and ω) were collected on a Bruker-AXS X8 Kappa Duo diffractometer coupled to a Smart APEX2 CCD detector with Mo Kα radiation (λ = 0.71073 Å) from an IμS micro-source. Absorption and other corrections were applied using SADABS.[6] The structure was solved by direct methods using SHELXT[7] and refined against F^2 on all data by full-matrix least squares with SHELXL-2015[8] using established refinement approaches.[9] All hydrogen atoms were included in the model at geometrically calculated positions and refined using a riding model. The isotropic displacement parameters of all hydrogen atoms were fixed to 1.2 times the U_{eq} value of the atoms they are linked to (1.5 times for methyl groups). Details about crystal

properties and diffraction data can be found in the table below. The program SQUEEZE[10] as implemented in PLATON[5] was used to account for the contribution of disordered solvent contained in voids within the crystal lattice. The solvent contribution was added to the model in a separate file (the .fab file) by SHELXL. Squeeze identified two crystallographically independent solvent accessible voids with a volume of 1106 Å^3. In these voids, Squeeze identified the equivalent of 179 electrons, corresponding to about 8 MeCN molecules.

Table S9: Crystallographic Data for [K(Kryptofix 222)][*m*BDCA-5t-H_5].

	[K(Kryptofix 222)][*m*BDCA-5t-H_5]
CCDC	1512972
Empirical formula, FW (g/mol)	$C_{81}H_{134}K_2N_8O_{22}$, 1650.15
Color/Morphology	Colorless/block
Crystal size (mm^3)	0.310×0.170×0.090
Temperature (K)	100(2)
Wavelength (Å)	0.71073
Crystal system, Space group	Monoclinic, C2/*c*
Unit cell dimensions (Å, deg)	a = 37.565(3), α = 90
	b = 20.3058(16), β = 90
	c = 22.5109(18), γ = 118.616(2)
Volume (Å^3)	15074(2)
Z	8
Density (calc., g/cm^3)	1.115
Absorption coefficient (mm^{-1})	0.130
$F(000)$	5456.0
Theta range for data collection (deg)	2.26-30.27
Index ranges	$-52 \leq h \leq 52$, $-29 \leq k \leq 28$, $-32 \leq l \leq 32$
Reflections collected	247025
Independent reflections, R_{int}	22903, 0.0802
Completeness to θmax (%)	0.990
Absorption correction	multi-scan
Refinement method	Full-matrix least-squares on F^2
Goodness-of-fit	1.103
Final R indices [$I > 2\sigma(I)$]	0.0552
R indices (all data)	0.0924
Largest diff. peak and hole ($e{\cdot}$Å^{-3})	0.447 and −0.423

6 Gas chromatography and mass spectrometry (GCMS) studies

6.1 Instrument configuration

An Agilent 5973 Network Mass Selective detector (Agilent Technologies, Santa Clara, CA) was used to examine the molar masses of the oxidation products. To determine of the amount of labeled ^{18}O, the mass spectra of the reaction mixture were compared to those of authentic $^{16}OPPh_3$ and ^{16}O-anthraquinone. Instrument configuration for solution analysis: MS detector: 70 eV; MS detector range: 100-650 amu; Inlet temperature: 250 °C; Injection pulse pressure: 20 psi, 1 min; Oven temperature: 100 °C, 5 min; 250 °C, 3 min; 320 °C, 8 min. Ramping between temperature set points: 20 and 30 °C/min; Total injection flow: 102 psi; Injection amount: 1 μL.

6.2 GCMS of the $OPPh_3$ produced from the reaction of [K(18-crown-6)]$_2$[$^{18}O_2 \subset m$BDCA-5t-H_6] and PPh_3 under CO_2 (1 atm)

[K_2DMF_3][$^{18}O_2 \subset m$BDCA-5t-H_6] (4.9 mg, 0.0041 mmol), 18-crown-6 (5.5 mg, 0.021 mmol, 5.1 equiv), and PPh_3 (3.5 mg, 0.013 mmol, 2.8 equiv) were dissolved in DMF (*ca*. 1 mL) and the resulting solution was transferred to a GCMS vial equipped with a septum. Excess CO_2 (1.00 mL, 1 atm, 25 °C, 0.0410 mmol, 10 eq) was added to the GCMS vial using a gas tight syringe equipped with a ball valve. The yellow color of [$^{18}O_2 \subset m$BDCA-5t-H_6]$^{2-}$ quickly bleached and afforded a colorless, homogeneous solution. After 10 minutes, a GCMS sample was prepared by diluting the reaction mixture (0.100 mL) with dichloromethane (0.900 mL). $^{18}OPPh_3$ was found to be the major product by GCMS (Figure S31).

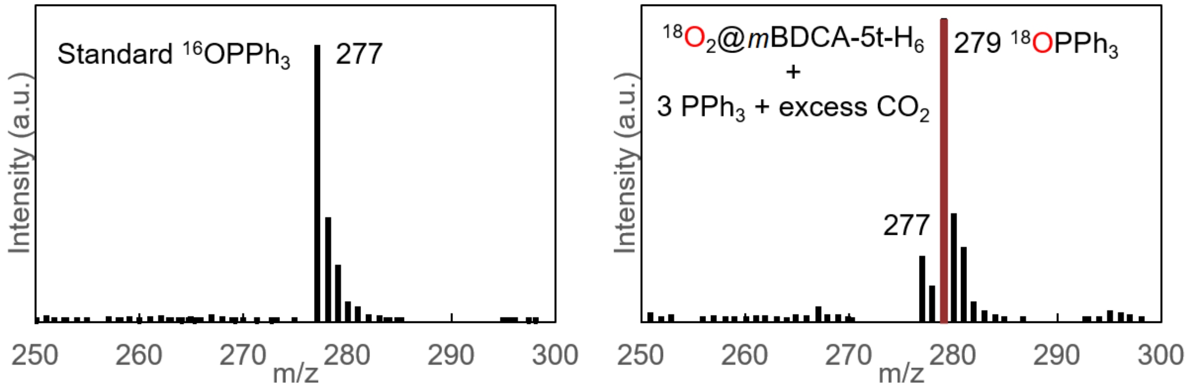

Figure S31: MS analysis of $^{16}OPPh_3$ standard (left) and the reaction mixture of [K(18-crown-6)]$_2$[$^{18}O_2 \subset$*m*BDCA-5t-H_6] and CO_2 in the presence of PPh_3 (right).

6.3 GCMS of the anthraquinone produced from the reaction of [K(18-crown-6)]$_2$[$^{18}O_2 \subset$*m*BDCA-5t-H_6] with DHA under CO_2 (1 atm)

[K_2DMF_3][$^{18}O_2 \subset$mBDCA-5t-H_6] (9.1 mg, 0.0061 mmol, 1 equiv), 18-crown-6 (4.3 mg, 0.016 mmol, 2.69 equiv) and DHA (1.5 mg, 0.0082 mmol, 1.3 equiv) were dissolved in DMF (*ca.* 0.5 mL) and the resulting solution was transferred to a GCMS vial equipped with a septum. Excess CO_2 (1.00 mL, 1 atm, 25 °C, 0.0410 mmol, 6.7 eq) was added to the GCMS vial using a gas tight syringe equipped with a ball valve. After 10 minutes, a GCMS sample was prepared by diluting the reaction mixture (0.100 mL) with dichloromethane (0.900 mL). Anthraquinone-$^{18}O^{16}O$, anthraquinone-$^{16}O_2$ and anthraquinone-$^{18}O_2$ were detected with anthraquinone-$^{16}O_2$ being the major product (Figure S32). The formation of anthraquinone can be rationalized by the C-H abstraction/radical recombination reaction sequence depicted in Scheme 1.

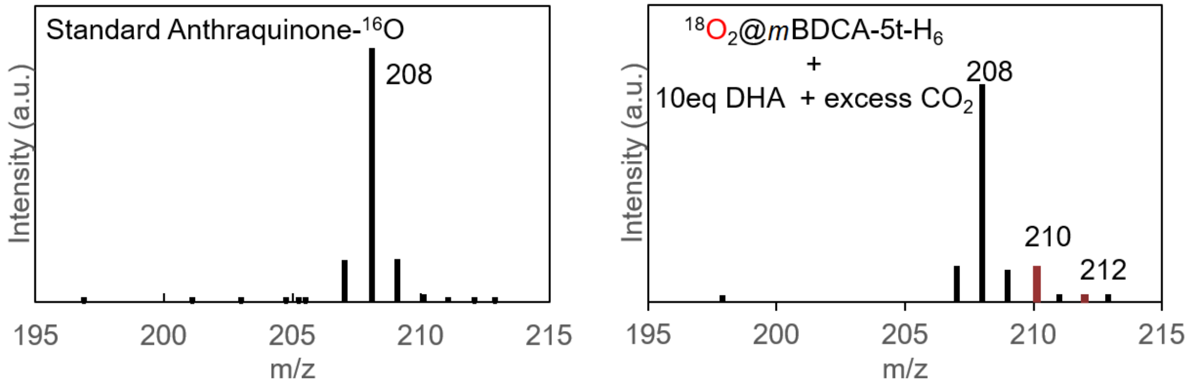

Figure S32: MS analysis of anthraquinone-O^{16} standard (left) and the reaction mixture of [K(18-crown-6)]$_2$[$^{18}O_2 \subset m$BDCA-5t-H_6] and CO_2 in the presence of DHA (right).

Scheme 1: ^{18}O scrambling via H-atom abstraction/radical recombination sequence.

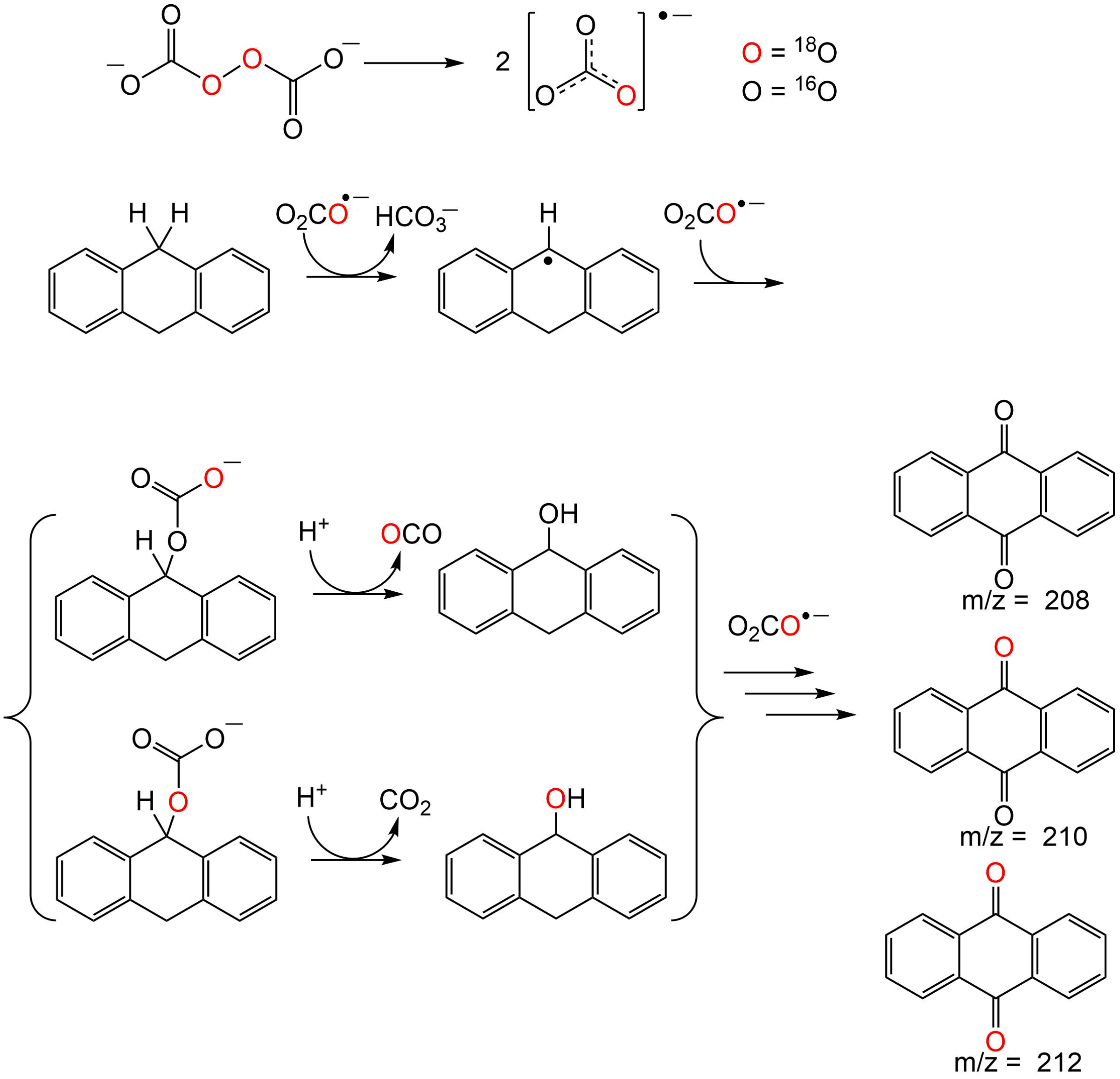

7 EPR studies

7.1 Materials and methods

The spin trap 5-*tert*-butoxycarbonyl-5-methyl-1-pyrroline-N-oxide (BMPO, Dojindo) was used as received. N,N-dimethylformamide (DMF) was dried over 3 Å molecular sieves for at least 3 weeks and vacuum distilled before use.

7.2 EPR spectroscopy

Continuous wave EPR experiments were performed on an ECS 106 (Bruker) at X-band (9.8 GHz). Spectra were collected using 1 G modulation amplitude at 100 kHz. Typical scan time was 160 s for all experiments. Simulations and least squares (LSQ) fittings were carried out with EasySpin toolbox (version 4.5.5) in Matlab (Mathworks Inc., Natick, MA).[11] Concentrations were estimated by fitting the spectra and then comparing the LSQ weight of the component with spin standard 4-Hydroxy-TEMPO (TEMPOL). Singular value decomposition (SVD) analysis was performed to facilitate data analysis.[12]

7.3 Mass spectrometry

Electrospray ionization high-resolution mass spectra (ESI-HRMS) of the spin trap samples were measured using an LTQ Orbitrap XL mass spectrometer equipped with an electrospray ionization source (ThermoFisher, San Jose, CA) operating in positive ion mode.

7.4 EPR sample preparation

Stock solutions of BMPO (0.100 M) and $[K(18\text{-crown-6})]_2[O_2\subset m\text{BDCA-5t-H}_6]$ (12 mM) were prepared using air-free manifold techniques. Solutions of $[K(18\text{-crown-6})]_2[O_2\subset m\text{BDCA-5t-H}_6]$ (50 μL, 12 mM, DMF) and BMPO (50 μL, 0.1 M, DMF) were transferred to an airtight EPR tubes to afford final concentrations of 6 mM $[K(18\text{-crown-6})]_2[O_2\subset m\text{BDCA-5t-H}_6]$ and 50 mM BMPO. For experiments involving CO_2, the BMPO solution and the EPR tubes were prepurged with CO_2 and the reaction was initiated by adding the peroxide cryptate and BMPO solution described above to this EPR tube containing CO_2. EPR analysis of the CO_2 purged sample showed a mixture of BMPO-OH, BMPO-O, and BMPO-OCO_2^- (Figure 3C-E, Table 2). Simulations and least squares (LSQ) fittings of the spectra were carried out with EasySpin toolbox (version 4.5.5) in Matlab (Mathworks Inc., Natick, MA).[11] A summary of simulation parameters are given in Table S10.

7.5 EPR standard preparation

The standard BMPO-OH solution was prepared by mixing iron(II) sulfate heptahydrate ($FeSO_4 \cdot 7H_2O$, Fisher, 1 mM, H_2O) and hydrogen peroxide (10 mM, H_2O) and BMPO (0.25 M, H_2O) followed by ten-fold dilution in DMF (Figure S33). The standard BMPO-O solution was prepared by adding potassium permanganate (10 μL, 10 mM, DMF) to BMPO-OH solution (40 μL, 50 mM, DMF). This solution was further diluted by twenty-fold in water for mass spectrometry analysis (Figure 3C-E).

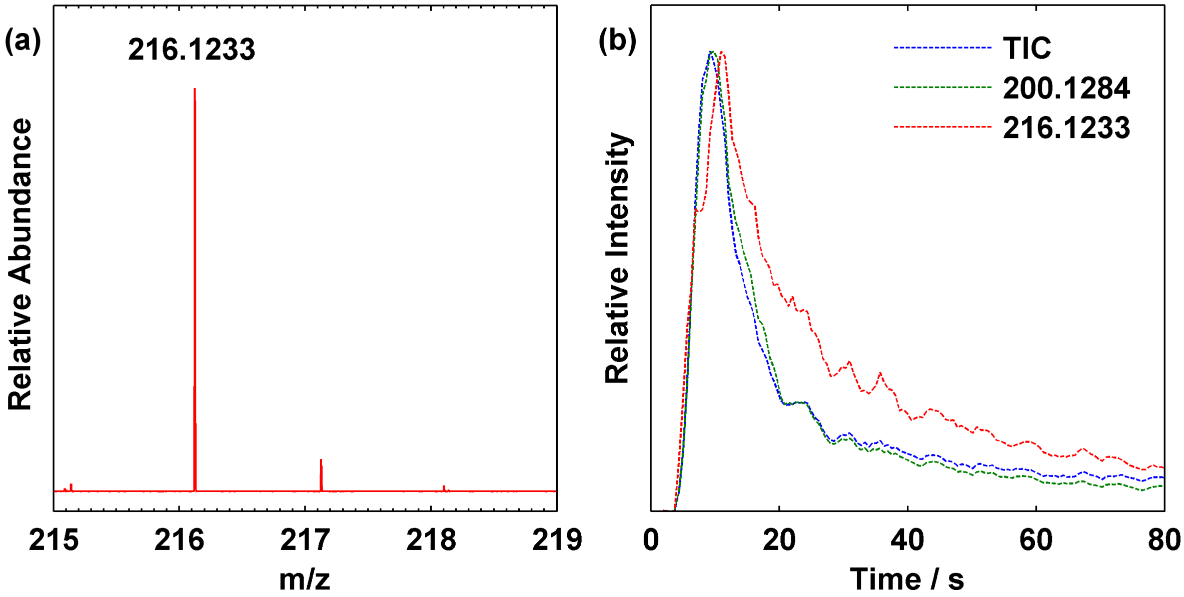

Figure S33: Independent preparation of BMPO-OH from BMPO and Fenton reagent (Fe^{2+} + H_2O_2). (a) HRMS spectrum (positive mode) and (b) chromatogram of BMPO-OH. Calculated m/z ($C_{10}H_{18}NO_4$): 216.1230. Found: 216.1237.

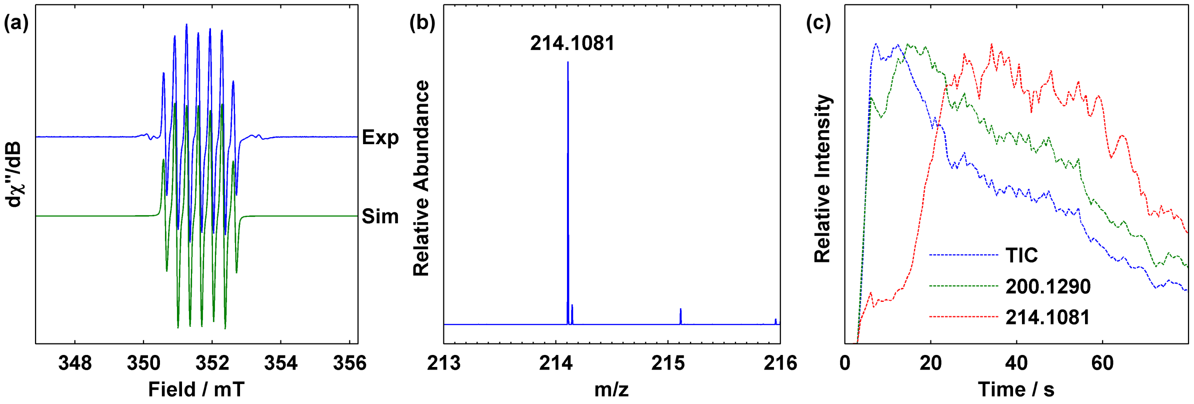

Figure S34: Independent preparation of BMPO-O from the reaction of $KMnO_4$ with BMPO-OH that was *in situ* generated from BMPO + Fenton reagent (Fe^{2+} + H_2O_2). (a) cw EPR spectrum and simulation of BMPO-O. (b) HRMS spectrum (positive mode) and (c) chromatogram of BMPO-O (observed as H^+ adduct). Calculated m/z ($C_{10}H_{16}NO_4$): 214.1074. Found: 214.1081.

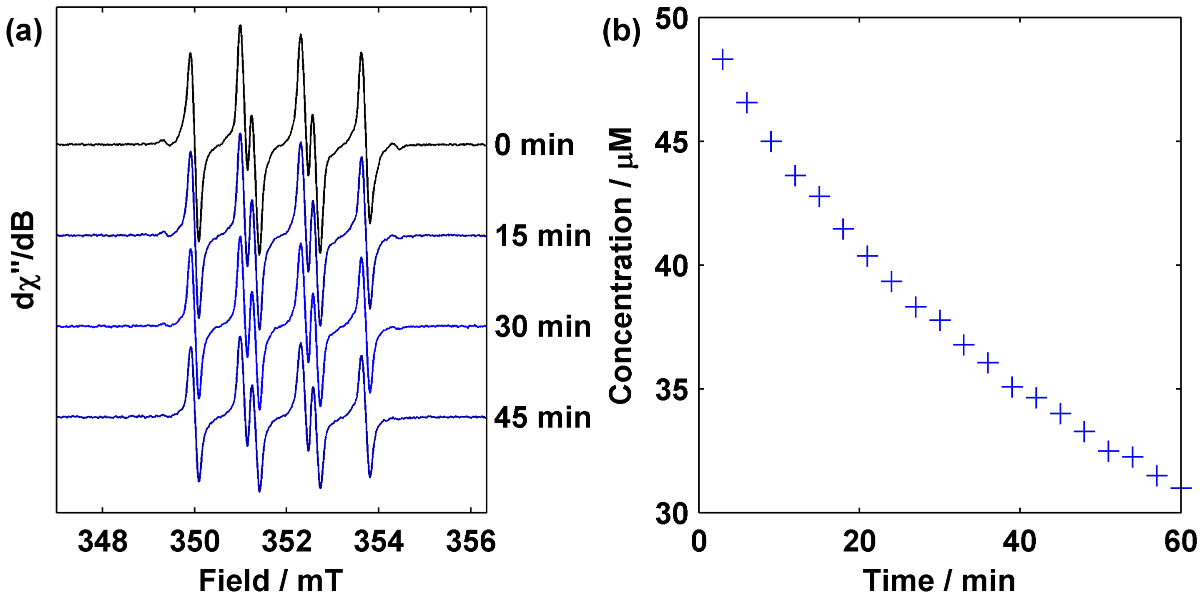

Figure S35: EPR spectrum of BMPO-OH prepared by mixing BMPO + Fenton reagent (Fe^{2+} + H_2O_2) in H_2O followed by dilution in DMF. (a) Time evolution of spectra. (b) Estimated concentration evolution over time.

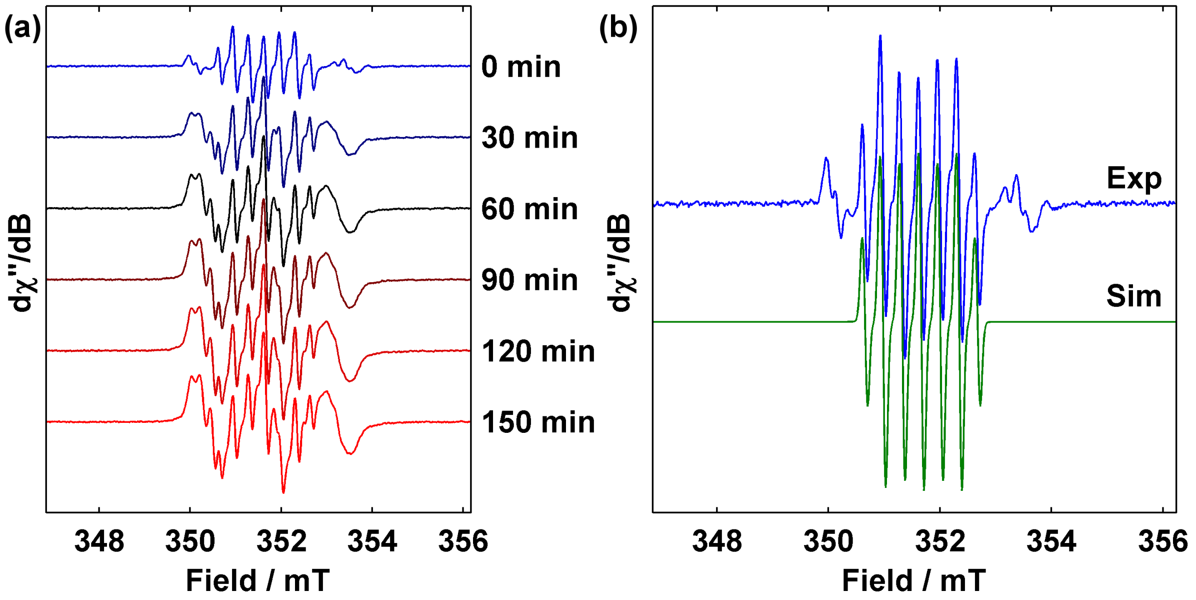

Figure S36: EPR spectra of [K(18-crown-6)]$_2$[O$_2$⊂*m*BDCA-5t-H$_6$] (24 mM) + BMPO after exposure to CO_2 in DMF. (a) Time evolution of spectra over 150 minutes. (b) Simulation of the major 7-peak component (green) and comparison to the first experimental spectrum (blue). See Table S10 below for simulation parameters.

Scheme 2: Formation of BMPO-O from BMPO-OH and $^{-}O_2COOH$.

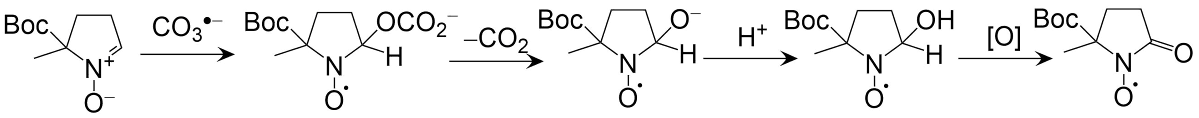

Table S10: Summary of hyperfine splitting parameters of observed radicals.

Species	αN/MHz	αN/MHz
BMPO-OCO$_2^-$	36.8	32.0; 15.6*
BMPO-OH	36.7	29.8
BMPO-O	19.2	9.3(×2)

*The apparent splitting of the signal for the suspected BMPO-OCO$_2^-$ adduct is unlikely to be a result of ^{1}H coupling but rather a mixture of conformers having different hyperfine splitting constants. Due to the extremely low concentration of this species, accurate simulation and extraction of hyperfine parameters is unlikely. An apparent proton hyperfine constant was used to simplify the simulation.

8 Solid state reactivity of [K(18-crown-6)]$_2$[O_2⊂*m*BDCA-5t-H_6] with CO_2

The following experiments were carried out to explore the reaction of CO_2 with [K(18-crown-6)]$_2$[O_2⊂*m*BDCA-5t-H_6] under solvent-free conditions, but in the presence of cryptand *m*BDCA-5t-H_6 as a potential stabilizer via hydrogen bonding of $HOOCO_2^-$ and $^-O_2COOCO_2^-$. Both symmetric peroxycarbonate (blue)[13] and unsymmetric peroxycarbonate (orange)[14] both have been isolated as solid before (Scheme 3). $Na_2C_2O_6$ has been observed previously by IR and Raman spectroscopy when CO_2 gas was introduced to solid sodium peroxide hydrate ($Na_2O_2{\cdot}8H_2O$), a compound in which a peroxide dianion unit is encased in a hydrogen bonding framework.[15]

Scheme 3: Synthesis of symmetric peroxycarbonate and unsymmetric peroxycarbonate.

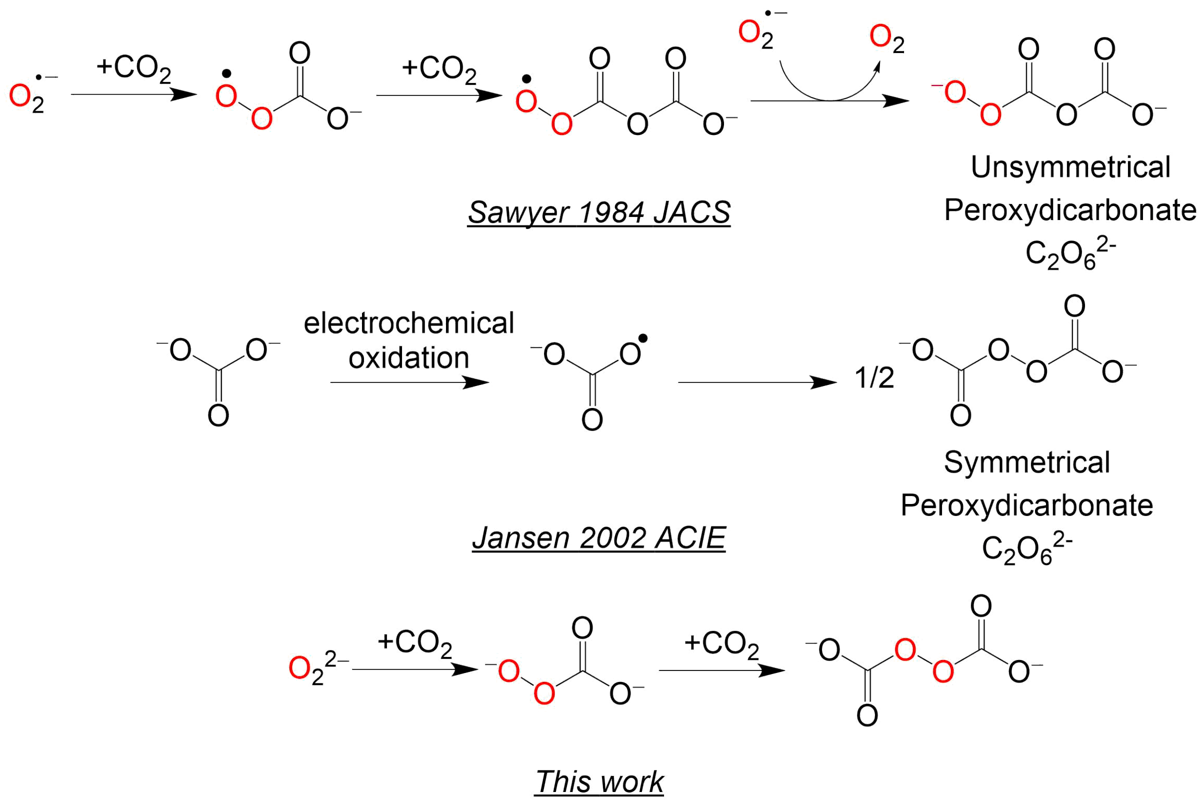

Similarly, treatment of solid samples of [K(18-crown-6)]$_2$[O_2⊂*m*BDCA-5t-H_6] with gaseous CO_2 (21 °C) resulted in a faint color changed from yellow to very pale orange. Analysis by ^{1}H NMR spectroscopy after dissolution of the resulting solids in DMSO-d_6 indicated the complete

consumption of [K(18-crown-6)]$_2$[$O_2$$\subset$*m*BDCA-5t-$H_6$] and conversion into a mixture (approximately 50:50) of [K(18-crown-6)]$_2$[$CO_3$$\subset$*m*BDCA-5t-$H_6$] and deprotonated cryptand *m*BDCA-5t-H_5^- via ^{1}H NMR integration with reference to an internal standard (18-crown-6). To probe for a possible intermediate that could be present in the solid samples after CO_2 exposure but which may rapidly oxidize the NMR solvent upon dissolution, a separate experiment was performed in which CO_2-treated solid [K(18-crown-6)]$_2$[$O_2$$\subset$*m*BDCA-5t-$H_6$] was dissolved using a DMSO-d_6 solution containing PPh_3 as an oxygen-atom acceptor. Conversion to PPh_3 was not observed, suggesting that an OAT oxidant such as $HOOCO_2^-$ was not present in the solid samples of [K(18-crown-6)]$_2$[$O_2$$\subset$*m*BDCA-5t-$H_6$] after treatment with CO_2. If formed under these conditions, $HOOCO_2^-$ or $^-O_2COOCO_2^-$ intermediate either decayed into carbonate prior to the dissolution or effected solvent oxidation upon the dissolution. Single crystal to single crystal transformation experiments were also attempted by exposing high quality crystals of [K(18-crown-6)]$_2$[$O_2$$\subset$*m*BDCA-5t-$H_6$] to CO_2 and remounting them on the diffractometer for X-ray analysis. The crystals of [K(18-crown-6)]$_2$[$O_2$$\subset$*m*BDCA-5t-$H_6$], however, were observed to lose crystallinity upon CO_2 treatment, precluding the intended analysis by X-ray crystallography. In addition, treatment of solid samples of **1**-$^{17}O_2$ with CO_2 followed by ^{17}O solid-state NMR spectroscopy revealed spectral features consistent with assignment to the anion-receptor carbonate complex [K(18-crown-6)]$_2$[$CO_3$$\subset$*m*BDCA-5t-$H_6$]. Diffuse Reflectance Infrared Fourier Transform Spectroscopy (DRIFTS) and low temperature, solid-state *in situ* Raman Spectroscopy were also used to monitor the reaction of solid [K(18-crown-6)]$_2$[$O_2$$\subset$*m*BDCA-5t-$H_6$] with gaseous CO_2, but intermediate species could not be identified by either of the two methods.

8.1 Experimental solid-state ^{17}O NMR details

The relevant ^{17}O SSNMR details can be found in the caption of Figure 5 of the main paper.

8.2 Treatment of solid [K(18-crown-6)]$_2$[O_2⊂*m*BDCA-5t-H_6] with CO_2 followed by dissolution in a solution containing PPh_3

A Schlenk flask (100 mL) was charged with [K(18-crown-6)]$_2$[O_2⊂*m*BDCA-5t-H_6] (0.103 g, 0.07 mmol, 1 equiv). The flask was sealed and removed from the glovebox. Once outside the glovebox, the flask was connected to a Schlenk line and placed under dynamic vacuum for 10 minutes. Then, the headspace of the flask was backfilled with CO_2 (1 atm, 4.4 mmol, 63 equiv) and allowed to stand for two hours. Then, the flask was evacuated and placed in the glovebox. Separately, a stock solution of PPh_3 in acetonitrile-d_3 (20 mg of PPh_3 in 1.000 mL of acetonitrile) was prepared. 184 μL (1 equiv PPh_3) of the stock solution was added to 1.000 mL of acetonitrile-d_3 using a calibrated pipette. This solution was frozen in the cold well of a glovebox and solid [K(18-crown-6)]$_2$[O_2⊂*m*BDCA-5t-H_6] (20.9 mg, 1 equiv relative to PPh_3 in the frozen solution) treated with CO_2 was added to the frozen solution. The frozen solution was allowed to warm up to 25 °C which resulted in complete dissolution of the solids. An aliquot of the reaction mixture was transfered to an NMR tube and ^{1}H and ^{31}P NMR spectra were acquired.

NMR analysis indicates that [K(18-crown-6)]$_2$[O_2⊂*m*BDCA-5t-H_6] converted to the carbonate cryptate (Figure S37), but $OPPh_3$ was not formed in this reaction (Figure S38), unlike in the previous section where adding CO_2 to a solution of [K(18-crown-6)]$_2$[O_2⊂*m*BDCA-5t-H_6] and PPh_3 was described (Figure S4). This observation suggests that the oxidizing equivalent formed upon adding CO_2 to [K(18-crown-6)]$_2$[O_2⊂*m*BDCA-5t-H_6] rapidly converted to the carbonate cryptate in the solid state.

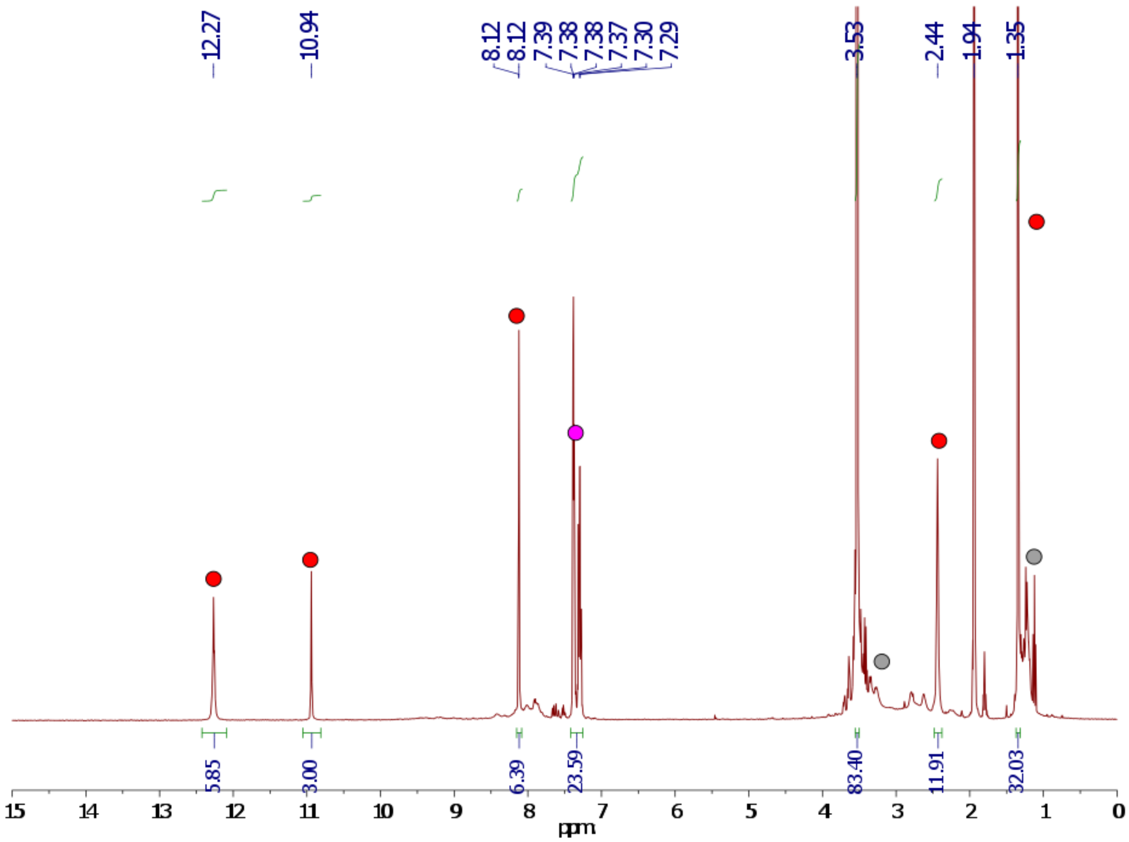

Figure S37: ^{1}H NMR (acetonitrile-d_3, 400 MHz, 21 °C) spectrum of the reaction mixture resulting from the treatment of [K(18-crown-6)]$_2$[$O_2 \subset m$BDCA-5t-H_6] with CO_2 in the solid state followed by dissolution in a solution of PPh_3 in acetonitrile-d_3. Diethyl ether is indicated by grey circles, carbonate cryptate by red circles, and PPh_3 by a pink circle.

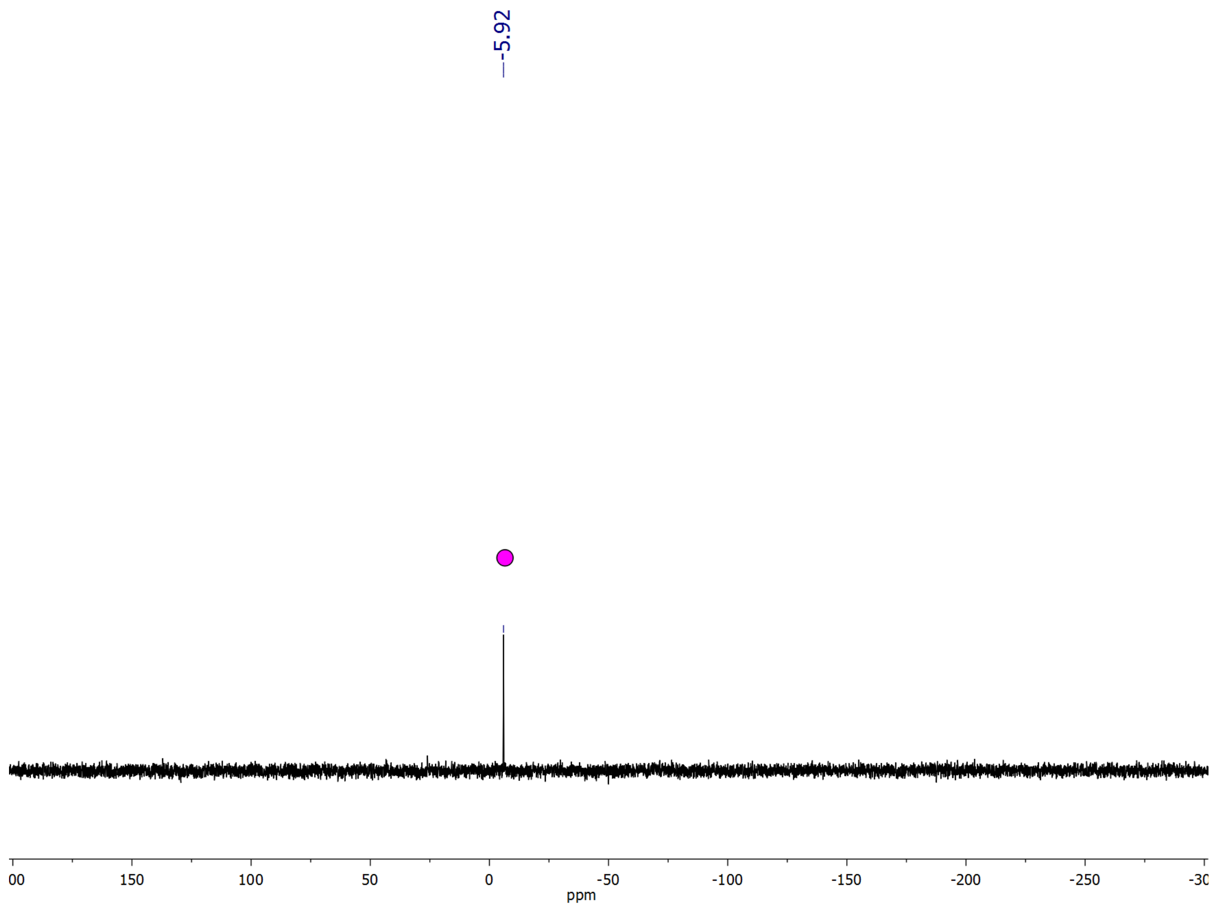

Figure S38: ^{31}P NMR (acetonitrile-d_3, 202 MHz, 21 °C) spectrum of the reaction mixture resulting from the treatment of [K(18-crown-6)]$_2$[O_2⊂*m*BDCA-5t-H_6] with CO_2 in the solid state followed by dissolution in a solution of PPh_3 in acetonitrile-d_3. PPh_3 is indicated by a pink circle. $OPPh_3$ (+25 ppm) is not visible in the spectrum.

8.3 DRIFTS measurements of [K(18-crown-6)]$_2$[O_2⊂*m*BDCA-5t-H_6] treated with CO_2 in the solid state

DRIFTS measurements (Diffuse Reflectance Infrared Fourier Transform Spectroscopy) were performed on a Bruker Tensor 37 Fourier transform IR (FTIR). A gas tight diffuse reflectance cell (Pike Instruments) equipped with a praying mantis apparatus was used. The signal intensity was preoptimized using KBr and recorded in absorbance mode. Prior to recording spectra, the cell was evacuated and an initial spectrum of [K(18-crown-6)]$_2$[O_2⊂*m*BDCA-5t-H_6] was taken. Then, CO_2 (Airgas, 99.995%, 500 mL, 1 atm) was admitted while recording spectra at 20 second inter-

vals (Figure S39). [K(18-crown-6)]$_2$[O_2⊂*m*BDCA-5t-H_6] was rapidly converted to [K(18-crown-6)]$_2$[CO_3⊂*m*BDCA-5t-H_6] within 40 seconds at 25 °C. Intermediate species could not be identified.

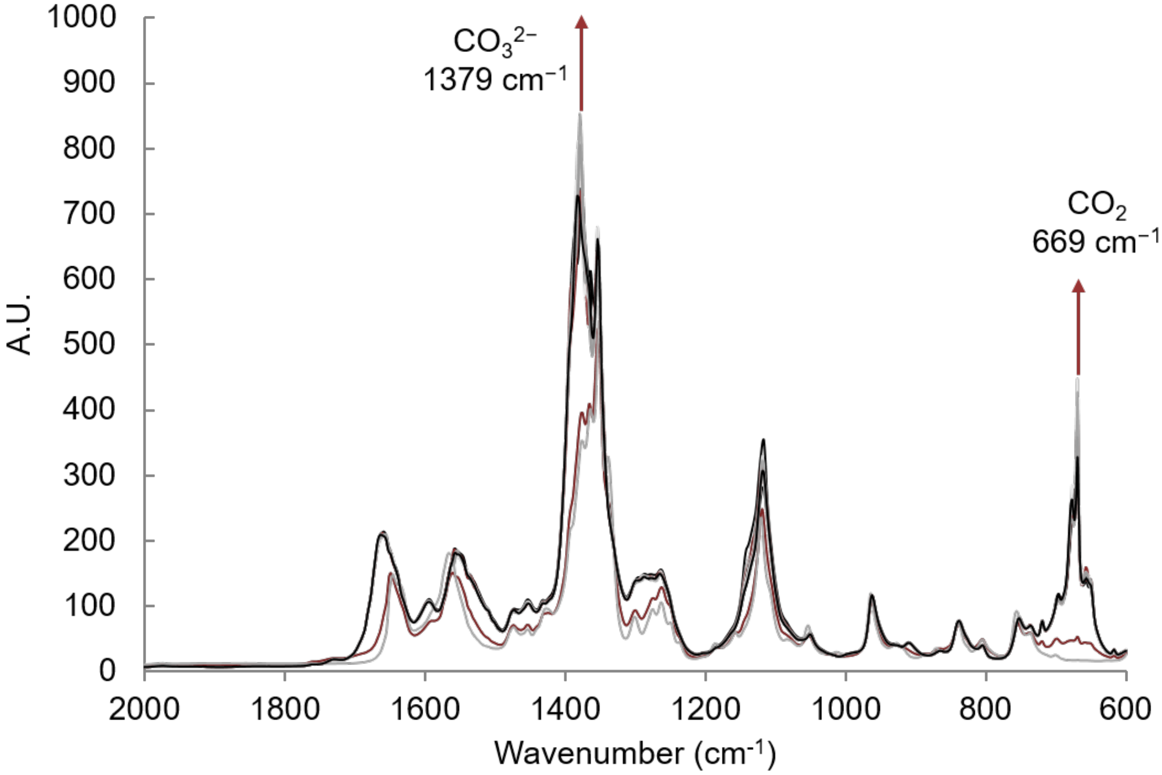

Figure S39: DRIFTS spectra of solid [K(18-crown-6)]$_2$[O_2⊂*m*BDCA-5t-H_6] before (red) and after (black) exposure to CO_2. The spectra are recorded in 20 second intervals.

8.4 Variable temperature RAMAN spectroscopy of [K(18-crown-6)]$_2$[O_2⊂*m*BDCA-5t-H_6] exposed to CO_2 in the solid state

Raman spectroscopy was performed on a Kaiser Optics Hololab series 5000 Raman microscope. Laser excitation was provided by an Invictus solid-state laser (785 nm) and the light was routed through 100 μm fiber optic cables to the microscope. Raman scattering was observed via 180° reflectance through the objective of the microscope. The scattered light was detected by a CCD cooled to −60 °C with a 785 nm filter. The average spectrum had a 30 second exposure and 10 accumulations. Data were processed using the Hololab software.

A sample of [K(18-crown-6)]$_2$[O$_2$⊂*m*BDCA-5t-H$_6$] (20 mg) was prepared in a glovebox and transferred to a Linkham FTIR600 Infrared freeze stage (Tadworth, Surrey, UK). A bulb of CO_2 (Airgas, 99.995%, 500 mL at 1 atm) was attached to the cell. An initial spectrum of [K(18-crown-6)]$_2$[O$_2$⊂*m*BDCA-5t-H$_6$] was taken at 25 °C. Then the cell was cooled to −80 °C and a spectrum was taken. Then the stopcock between the cell and the CO_2 bulb was opened. Spectra (Figure S40) were taken at −80, −50, −30, 0, and 25 °C (Figure S40). After acquisition of the spectra, a ^{1}H NMR spectrum (Figure S41) was taken to confirm that carbonate cryptate had formed.

A meaningful interpretation of the Raman data could not be made on account of the similarity of the spectrum of the starting and ending points. In addition, low temperature spectra exhibited broadening or disappearance of some peaks, also making *in situ* interpretation and identification of intermediates impossible.

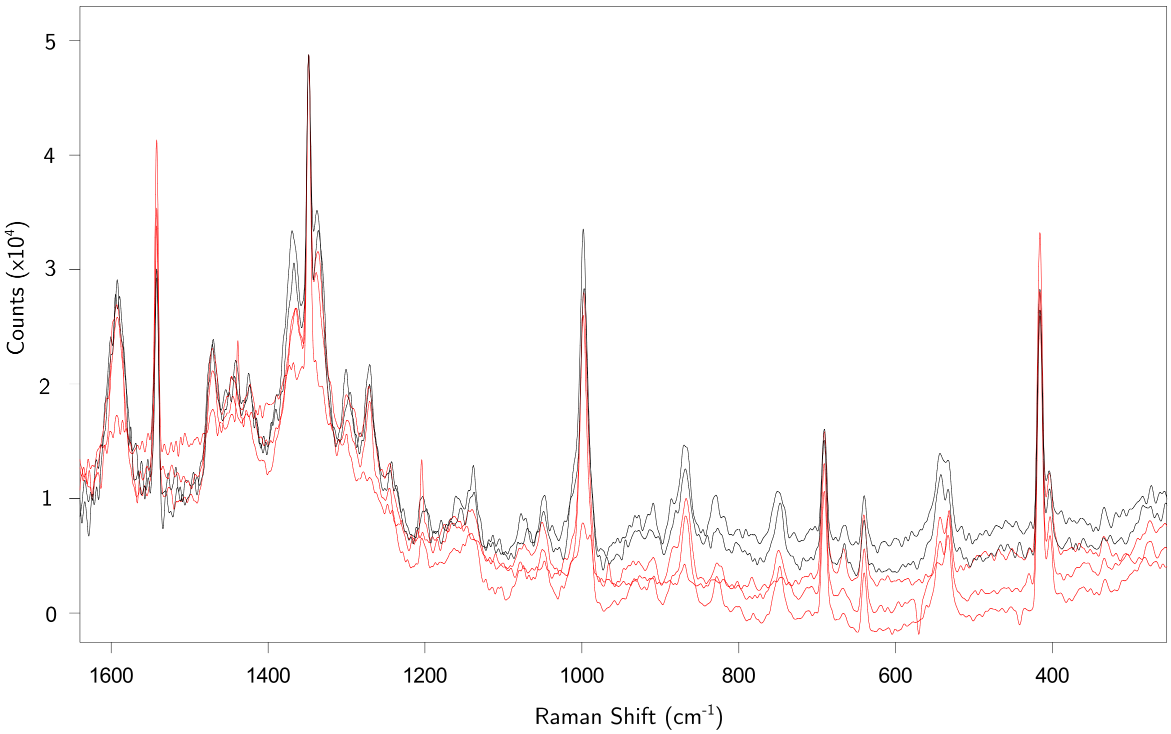

Figure S40: Raman spectra of solid [K(18-crown-6)]$_2$[O$_2$⊂*m*BDCA-5t-H$_6$] exposed to excess CO_2 at various temperatures. The starting spectrum at 25 °C and at −80 °C before adding CO_2 are represented as the black spectra. The red spectra are representative after the addition of CO_2 and due to the lack of differentiation between the spectra, some temperatures are omitted.

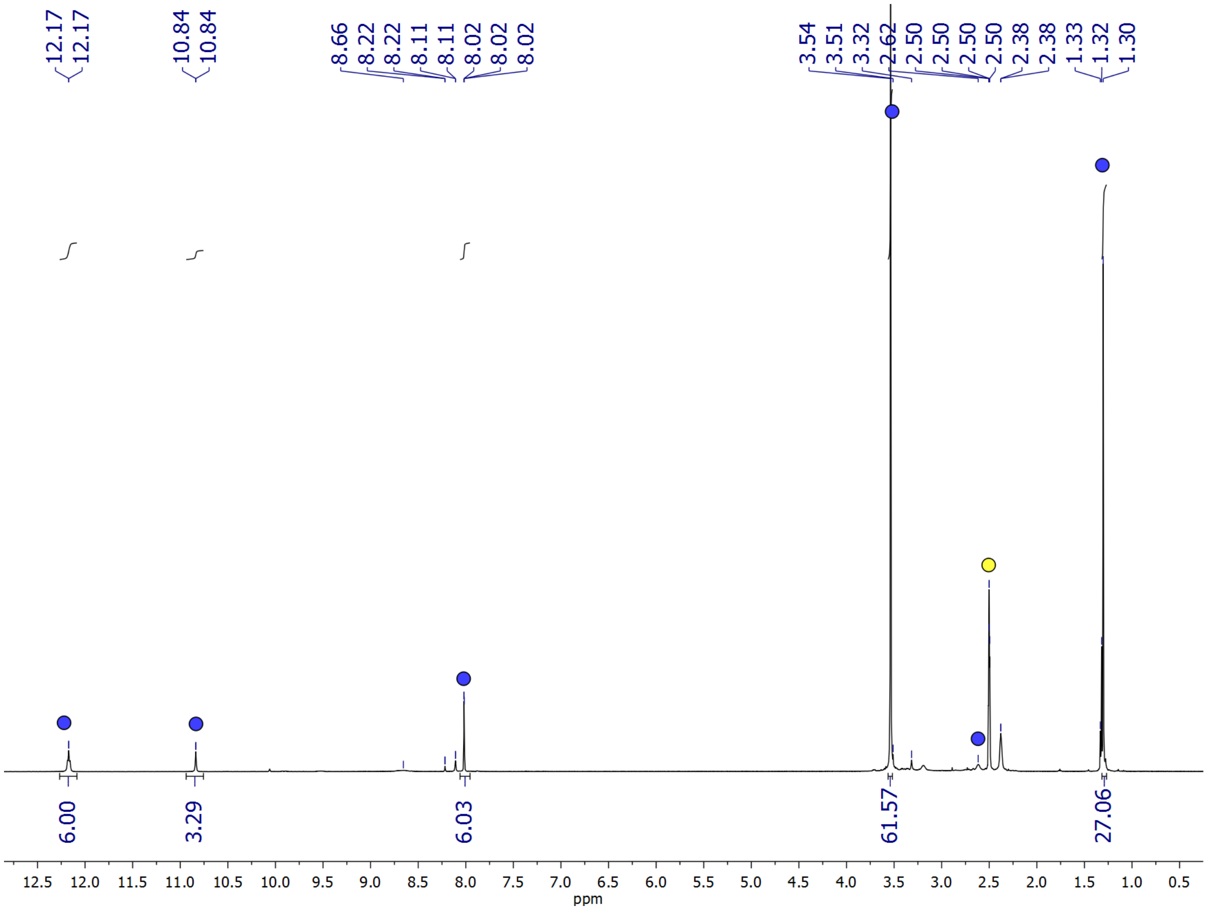

Figure S41: ^{1}H NMR (DMSO-d_6, 400 MHz, 21 °C) spectrum of $[K(18\text{-crown-}6)]_2[O_2{\subset}m\text{BDCA-5t-H}_6]$ used in the above Raman experiment after the acquisition of the Raman spectra. Formation of the carbonate cryptate (blue circles) is clearly observed. The DMSO peak is indicated by a yellow circle.

8.5 Gas Chromatography (GC) of the reaction vessel headspace after exposure of $[K(18\text{-crown-}6)]_2[O_2{\subset}m\text{BDCA-5t-H}_6]$ to CO_2

Gas Chromatograms were obtained using an Agilent 7890A gas chromatograph equipped with a thermal conductivity detector and a high surface area carbon column. Instrumental parameters were used such that the retention time of oxygen was 1.2 min and CO_2 was 3.1 minutes.

In the glovebox, an Agilent 2 mL GC vial equipped with a silicone/ptfe septum cap was charged with $[K(18\text{-crown-}6)]_2[O_2{\subset}m\text{BDCA-5t-H}_6]$ (14 mg and 20 mg). A third vial was prepared which only contained the atmosphere of the glovebox as a control. The three vials were removed from

the glovebox. Once outside the glovebox, CO_2 (700 μL, 1 atm, 25 °C, *ca*. 3 eq) was injected into the two vials containing [K(18-crown-6)]$_2$[$O_2$$\subset$*m*BDCA-5t-$H_6$] with a gas tight syringe equipped with a ball valve. The control vial was injected with N_2 (700 μL). The vials were allowed to stand for four minutes and then 50 μL of the headspace of the corresponding vials was then injected into the GC. The GC traces (Figure S42) indicate that while [K(18-crown-6)]$_2$[$O_2$$\subset$*m*BDCA-5t-$H_6$] appears to react with CO_2 based on the differential amount of CO_2 left in the headspace, oxygen gas could not be detected, indicating that the product of the reaction is not oxygen.

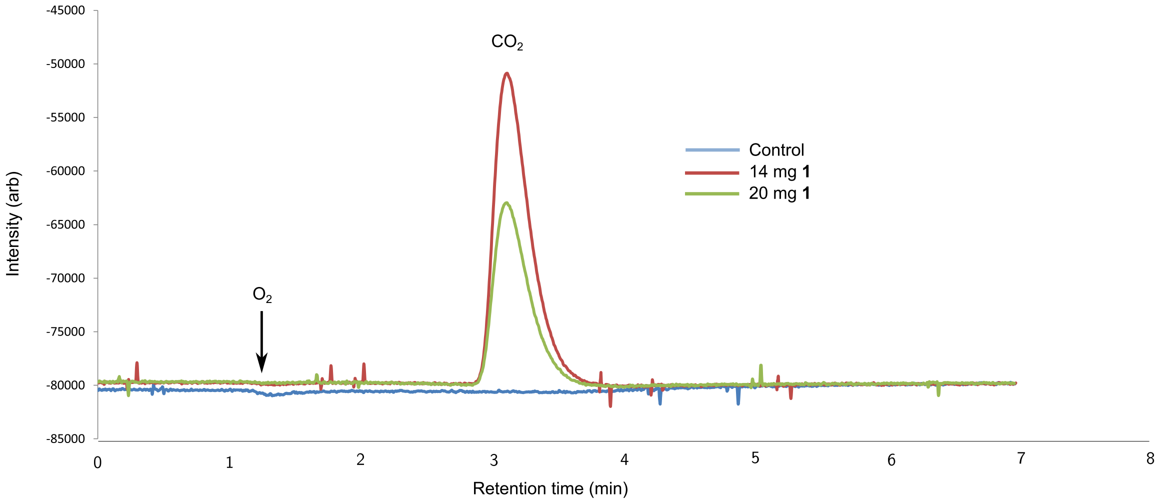

Figure S42: GC traces of the headspace of the reaction vessel after treatment of solid [K(18-crown-6)]$_2$[$O_2$$\subset$*m*BDCA-5t-$H_6$] with CO_2.

9 Ab initio calculation details

9.1 Computational Methodology

Geometry optimizations were performed using the program Gaussian 09[16] at the B3LYP/6-311G++(2d,2p)[17–21] level of theory. Frequency calculations were performed to ensure all structures were at global minima. Hydroperoxycarbonate, peroxycarbonate, symmetrical peroxydicarbonate (dianion and monoprotonated), and unsymmetrical peroxydicarbonate (dianion and monoprotonated) were considered as possible species to correspond to the ^{13}C NMR signals observed

during the course of the reaction of [K(18-crown-6)]$_2$[O_2⊂*m*BDCA-5t-H_6] with CO_2. The coordinates from the optimized structures of peroxycarbonate and peroxydicarbonate are deposited in the appendix of this supplementory material and were then used to perform an NMR calculation with the program Gaussian 09 using the GIAO method[22–26] with the same level of theory as the geometry optimizations. These calculations was performed using Gaussian 09 using keywords: nmr=giao rb3lyp/6-311++g(2d,2p) scrf=(iefpcm,solvent=dmso). A plot of experimental *vs*. calculated absolute chemical shielding was constructed using experimentally known compounds (DMF, DMSO, CO, CO_2, CO_3^{2-}) as calibration standards.

9.2 ^{13}C NMR calculation

To provide an accurate NMR prediction, an experimental chemical shift (δ) *vs*. calculated absolute chemical shielding (σ) plot (Figure S43) was generated using known carbon containing compounds as calibration standards in order to minimize errors due to basis set effects.

Table S11: Calculated absolute chemical shielding σ *vs*. experimental ^{13}C NMR chemical shift δ (ppm).

Entry	Calculated σ	Predicted δ	Observed δ
CO	-11.12	X	185.41
CO_2	51.42	X	124.21
DMF (carbonyl)	11.85	X	162.29
DMF (methyl)	144.57	X	35.73
DMF (methyl)	146.40	X	30.73
DMSO	137.48	X	39.52
CO_3^{2-} (from carbonate cryptate)	1.21	X	172.2
Peroxycarbonate	5.89	173.1	X
Hydroperoxycarbonate	13.52	165.25	157.4
Peroxydicarbonate (symmetrical)	19.07	159.54	156.9
Peroxydicarbonate (unsymmetrical)	13.92	164.85	X
Peroxydicarbonate (unsymmetrical)	27.90	150.45	X

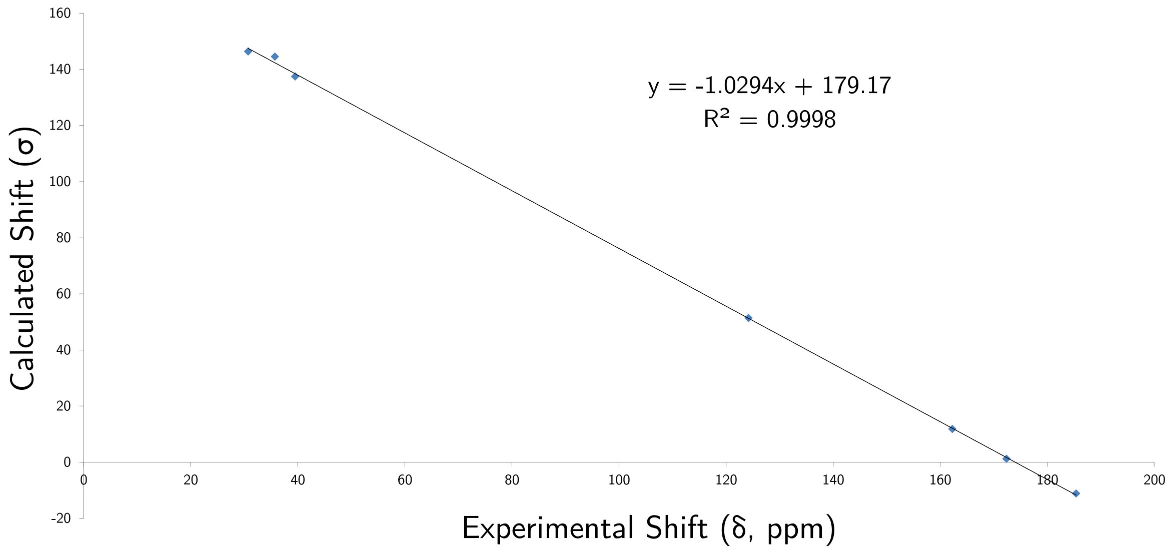

Figure S43: Experimental ^{13}C NMR chemical shifts (δ) *vs*. calculated absolute chemical shielding (σ).

9.3 ^{17}O NMR calculation

To provide an accurate NMR prediction, an experimental chemical shift (δ) *vs*. calculated absolute chemical shielding (σ) plot (Figure S44) was generated using known oxygen containing compounds as calibration standards in order to minimize errors due to basis set effects.

Table S12: Calculated absolute chemical shielding σ *vs*. experimental ^{17}O NMR chemical shift δ (ppm).

Entry	Calculated σ	Predicted δ	Observed δ
H_2O	330.8	X	0
CO_2	51.4	X	77.5
H_2O_2	109.7	X	204.6
DMF	-22.3	X	324.3
THF	276.4	X	17.83
Methyl Acetate	326.3	X	360.5
Methanol	326.3	X	-30.1
Peroxydicarbonate $O_2C\mathbf{OO}CO_2$	-58.6	351.9	X
Peroxydicarbonate $\mathbf{O_2}COOC\mathbf{O_2}$	112.9	188.8	X
Hydroperoxycarbonate $H\mathbf{O}OCO_2$	-2.0	298.0	278.7
Hydroperoxycarbonate $HO\mathbf{O}CO_2$	28.4	269.1	264.0
Hydroperoxycarbonate $HOOC\mathbf{O_2}$	117.3	184.6	X

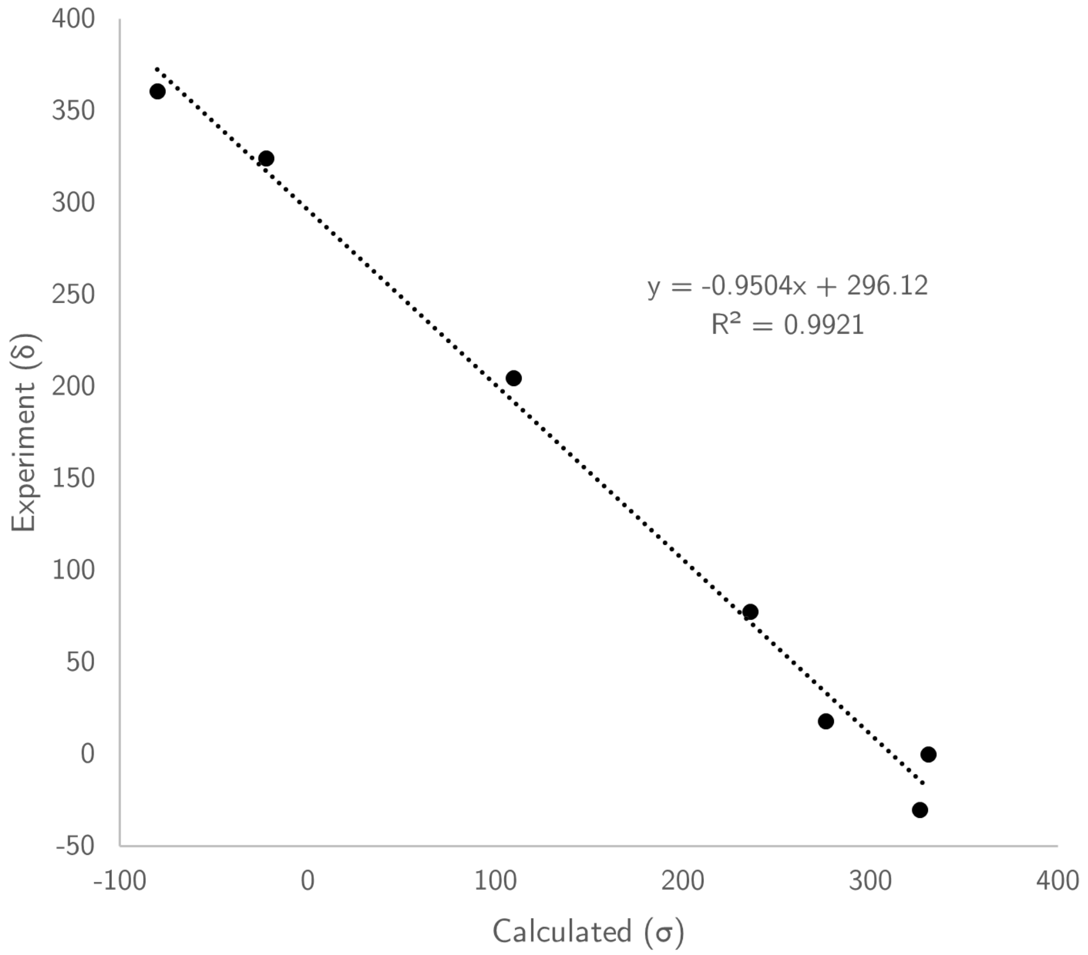

Figure S44: Experimental ^{17}O NMR chemical shifts (δ) *vs*. calculated absolute chemical shielding (σ).

9.4 Determination of the O–O Bond Dissociation Enthalpy (BDE) in $^{-}O_2COOCO_2^{-}$

Having established that the symmetrical peroxydicarbonate is present in the reaction mixture resulting from the treatment of $[K(18\text{-crown-6})]_2[O_2{\subset}m\text{BDCA-5t-H}_6]$ with CO_2 at low temperatures, we sought to understand the decomposition pathway(s) of this anion that has not previously been characterized in aprotic media. A common intramolecular decomposition pathway of dicarbonates is through thermally induced O–O bond homolysis. The O–O bond BDE of peroxydicar-

bonate is not known, however a study predicts that the dimerization of carbonate radical anions to peroxydicarbonate is highly unfavorable in aprotic media.[27] To determine the bond dissociation energy of symmetrical peroxydicarbonate, the optimized equilibrium structures of peroxydicarbonate dianion and carbonate radical anion were determined and frequency calculations were performed to determine both calculated structures corresponded to local minima using the program Gaussian 09 at the MP2/6-311G++(2d,2p)[28] level of theory. An IEFPCM solvation model (DMSO) was used.[29,30] The coordinates of the optimized structures are deposited in the appendix of this supplementary material. The homolytic BDE, defined as the enthalpy change of equation 7 at 298 K depicted below and includes the electronic energy and zero-point correction:

$$C_2O_6{}^{2-} \xrightarrow[298\ \mathrm{K}]{} 2CO_3{\cdot}^{-} \qquad (7)$$

The O–O BDE ($\Delta H_{298\mathrm{K}}$) of symmetrical peroxydicarbonate was found to be 15.9 kcal/mol. This value would place it among the weakest O–O bonds known, particularly for anionic compounds, with the BDE (experimental) of peroxydisulfate being ~30 kcal/mol.[31]

10 Appendix

10.1 Coordinates of peroxycarbonate optimized at the B3LYP/6-311G++(2d,2p) level of theory used for GIAO NMR calculations

ATOM	X	Y	Z
C	0.00000000	0.55012600	0.00000000
O	1.24759800	0.62982600	0.00000000
O	-0.83143800	1.50583300	0.00000000
O	-0.63464400	-0.67350100	0.00000000
O	0.21848400	-1.87475300	0.00000000

10.2 Coordinates of hydroperoxycarbonate optimized at the B3LYP/6-311G++(2d,2p) level of theory used for GIAO NMR calculations

ATOM	X	Y	Z
C	0.00000000	0.55012600	0.00000000
O	1.24759800	0.62982600	0.00000000
O	-0.83143800	1.50583300	0.00000000
O	-0.63464400	-0.67350100	0.00000000
O	0.21848400	-1.87475300	0.00000000
H	1.17848400	-1.87475300	0.00000000

10.3 Coordinates of symmetrical peroxydicarbonate optimized at the B3LYP/6-311G++(2d,2p) level of theory used for GIAO NMR calculations

ATOM	X	Y	Z
C	1.33054900	1.25199000	0.00000000
O	2.28691200	0.47029500	0.00000000
O	1.27731900	2.49729200	0.00000000
O	0.00000000	0.73552300	0.00000000
O	0.00005700	-0.73561100	0.00000000
C	-1.33057800	-1.25204800	0.00000000
O	-1.27738300	-2.49731600	0.00000000
O	-2.28688300	-0.47013900	0.00000000

10.4 Coordinates of unsymmetrical peroxydicarbonate optimized at the B3LYP/6-311G++(2d,2p) level of theory used for GIAO NMR calculations

ATOM	X	Y	Z
C	0.55424900	-0.52359400	0.00000000
O	-0.01268000	-1.59423100	0.00000000
O	0.00000000	0.71519200	0.00000000
O	1.86973600	-0.31480000	0.00000000
O	2.68731400	-1.52532500	0.00000000
C	-1.46455600	1.02640900	0.00000000
O	-2.25488900	0.08998700	0.00000000
O	-1.60675100	2.25206500	0.00000000

10.5 Coordinates of symmetrical peroxydicarbonate optimized at the MP2/6-311G++(2d,2p) level of theory used for BDE calculations

ATOM	X	Y	Z
C	-1.60694500	-0.14319700	-0.08188900
O	-1.33221100	-1.13257200	-0.76552100
O	-2.63838000	0.54859400	0.00606100
O	-0.61817000	0.37861000	0.82089800
O	0.61824500	-0.37875700	0.82080400
C	1.60690800	0.14318800	-0.08185500
O	1.33216900	1.13269300	-0.76541000
O	2.63837500	-0.54855900	0.00597500

10.6 Coordinates of the carbonate radical anion optimized at the MP2/6-311G++(2d,2p) level of theory used for BDE calculations

ATOM	X	Y	Z
C	0.00067700	0.00003900	0.00000200
O	-0.65514500	-1.08684300	0.00000000
O	1.26941900	-0.02329100	0.00000000
O	-0.61478300	1.11010500	0.00000000

References

(1) Hoult, D. *J. Magn. Reson.* **1976**, *21*, 337.

(2) Albers, A. E.; Okreglak, V. S.; Chang, C. J. *J. Am. Chem. Soc.* **2006**, *128*, 9640–9641.

(3) Dönges, E. In *Handbook of Preparative Inorganic Chemistry, 2nd Ed.*; Brauer, G., Ed.; Academic Press, 1963; Chapter Lithium and Sodium Peroxides, p 979.

(4) Jones, M. L. M. Ph.D. thesis. 1994.

(5) Spek, A. L. *Acta Crystallogr. D* **2009**, *65*, 148–155.

(6) Krause, L.; Herbst-Irmer, R.; Sheldrick, G. M.; Stalke, D. *J. Appl. Crystallogr.* **2015**, *48*, 3–10.

(7) Sheldrick, G. M. *Acta Crystallogr. A* **2015**, *71*, 3–8.

(8) Sheldrick, G. M. *Acta Crystallogr. C* **2015**, *71*, 3–8.

(9) Müller, P. *Crystallogr. Rev.* **2009**, *15*, 57–83.

(10) van der Sluis, P.; Spek, A. L. *Acta Crystallogr. A* **1990**, *46*, 194–201.

(11) Stoll, S.; Schweiger, A. *J. Magn. Reson.* **2006**, *178*, 42–55.

(12) Keszler, A.; Hogg, N. *Biol. Magn. Reson.* **2005**, *23*, 111–123.

(13) Dinnebier, R. E.; Vensky, S.; Stephens, P. W.; Jansen, M. *Angew. Chem. Int. Ed.* **2002**, *41*, 1922–1924.

(14) Roberts, J. L.; Calderwood, T. S.; Sawyer, D. T. *J. Am. Chem. Soc.* **1984**, *106*, 4667–4670.

(15) Hill, G. S.; Holah, D. G.; Kinrade, S. D.; Sloan, T. A.; Magnuson, V. R.; Polyakov, V. *Can. J. Chem.* **1997**, *75*, 46–51.

(16) Frisch, M. J. et al. Gaussian09 Revision D.01. Gaussian Inc. Wallingford CT 2009.

(17) Lee, C.; Yang, W.; Parr, R. G. *Phys. Rev. B* **1988**, *37*, 785–789.

(18) Perdew, J. P.; Burke, K.; Ernzerhof, M. *Phys. Rev. Lett.* **1996**, *77*, 3865–3868.

(19) McLean, A. D.; Chandler, G. S. *J. Chem. Phys.* **1980**, *72*, 5639–5648.

(20) Krishnan, R.; Binkley, J. S.; Seeger, R.; Pople, J. A. *J. Chem. Phys.* **1980**, *72*, 650–654.

(21) Clark, T.; Chandrasekhar, J.; Spitznagel, G. W.; Schleyer, P. V. R. *J. Comp. Chem.* **1983**, *4*, 294–301.

(22) Cheeseman, J. R.; Trucks, G. W.; Keith, T. A.; Frisch, M. J. *J. Chem. Phys.* **1996**, *104*, 5497–5509.

(23) Wolinski, K.; Hinton, J. F.; Pulay, P. *J. Am. Chem. Soc.* **1990**, *112*, 8251–8260.

(24) Ditchfield, R. *Mol. Phys.* **1974**, *27*, 789–807.

(25) McWeeny, R. *Phys. Rev.* **1962**, *126*, 1028–1034.

(26) London, F. *J. Phys. Radium* **1937**, *8*, 397–409.

(27) Armstrong, D. A.; Waltz, W. L.; Rauk, A. *Can. J. Chem.* **2006**, *84*, 1614–1619.

(28) Head-Gordon, M.; Pople, J. A.; Frisch, M. J. *Chem. Phys. Lett.* **1988**, *153*, 503–506.

(29) Miertuš, S.; Scrocco, E.; Tomasi, J. *Chem. Phys.* **1981**, *55*, 117–129.

(30) Miertuš, S.; Tomasi, J. *Chem. Phys.* **1982**, *65*, 239–245.

(31) Sanderson, R. T. *Chemical Bonds and Bond Energy (Physical Chemistry), 2nd Ed.*; Academic Press Inc., 1971.
